# Prognostic Factors for Adulthood Psychosis in Adolescent Psychiatry Services: A Longitudinal Total Birth Cohort Study

**DOI:** 10.1093/schbul/sbag031

**Published:** 2026-06-04

**Authors:** Ulla Lång, Kirstie O’Hare, Fiona Boland, Johanna Metsälä, Anna Pulakka, Juha Veijola, Eero Kajantie, Colm Healy, Ian Kelleher

**Affiliations:** Institute for Neuroscience and Cardiovascular Research, University of Edinburgh, Edinburgh EH16 4SB, United Kingdom; Department of Public Health and Welfare, Finnish Institute for Health and Welfare, 00271 Helsinki, Finland; Research Unit of Clinical Medicine, University of Oulu, 90014 Oulu, Finland; Institute for Neuroscience and Cardiovascular Research, University of Edinburgh, Edinburgh EH16 4SB, United Kingdom; Discipline of Psychiatry and Mental Health, University of New South Wales, NSW 2031 Sydney, Australia; Data Science Centre, School of Population Health, RCSI University of Medicine and Health Sciences, D02 Dublin, Ireland; Institute for Neuroscience and Cardiovascular Research, University of Edinburgh, Edinburgh EH16 4SB, United Kingdom; Department of Public Health and Welfare, Finnish Institute for Health and Welfare, 00271 Helsinki, Finland; Research Unit of Clinical Medicine, University of Oulu, 90014 Oulu, Finland; Research Unit of Population Health, University of Oulu, 90014 Oulu, Finland; Research Unit of Clinical Medicine, University of Oulu, 90014 Oulu, Finland; Department of Psychiatry, Oulu University Hospital, 90230 Oulu, Finland; Medical Research Center Oulu, Oulu University Hospital and University of Oulu, 90220 Oulu, Finland; Department of Public Health and Welfare, Finnish Institute for Health and Welfare, 00271 Helsinki, Finland; Research Unit of Clinical Medicine, University of Oulu, 90014 Oulu, Finland; Department of Clinical and Molecular Medicine, Norwegian University of Science and Technology, 7491 Trondheim Norway; Institute for Neuroscience and Cardiovascular Research, University of Edinburgh, Edinburgh EH16 4SB, United Kingdom; School of Medicine, University College Dublin, D04 Dublin, Ireland; Institute for Neuroscience and Cardiovascular Research, University of Edinburgh, Edinburgh EH16 4SB, United Kingdom; Research Unit of Clinical Medicine, University of Oulu, 90014 Oulu, Finland; School of Medicine, University College Dublin, D04 Dublin, Ireland; St John of God Hospitaller Services Group, A94 FH92 Stillorgan, Dublin, Ireland

**Keywords:** psychosis risk, young people, register based study

## Abstract

**Background and Hypothesis:**

As many as half of all psychotic disorders diagnosed by age 28 in the population emerge in individuals who attended child and adolescent psychiatry services, highlighting important opportunities for psychosis prediction and prevention. An important next step is to identify prognostic factors for later psychotic disorders within this clinical population. We assessed a large number of potential prognostic factors for adult-onset psychosis within adolescent psychiatry services.

**Study Design:**

Linked population-wide register data for all individuals born in Finland 1987-1992. Using survival analyses, we assessed a range of clinical, service use, and sociodemographic characteristics in adolescent psychiatry patients (ie, individuals who were diagnosed with a mental disorder in specialist-level services in adolescence [ages 13-17 years]) as prognostic factors for adult-onset psychotic disorders (ages 18-30 years; individuals diagnosed with psychosis before age 18 [*n* = 2276] were excluded).

**Study Results:**

Among adolescent psychiatry patients (*n* = 27 626), cumulative risk of psychosis between ages 18 and 30 was 8.5%. Within-service significant prognostic factors for psychosis included: total number of mental disorder diagnoses received in adolescence, young maternal age, premature birth, older age at first adolescent psychiatry contact, history of child psychiatry contact, psychiatric inpatient admission in adolescence, parental history of psychosis, and parental history of inpatient psychiatric admission.

**Conclusions:**

Our findings demonstrate that a number of clinical, service use, and sociodemographic factors have prognostic significance for the development of adulthood psychosis among adolescent psychiatry patients.

## Introduction

Psychotic disorders such as schizophrenia cause a major burden on individuals, families, and society.^1–6^ Early detection of psychosis risk is a key research priority in psychiatric research.^7–10^ One approach to identifying young people at risk of psychosis is to look within child and adolescent psychiatry services.^11^ Recent longitudinal research has shown that as many as half of individuals diagnosed with a psychotic disorder by age 28 had attended specialist psychiatry services in childhood and adolescence.^12^^,^^13^ This finding highlights important opportunities for early psychosis prediction and, ultimately, prevention within these services.

An important next step for research is to investigate whether particular prognostic factors help to identify subgroups of adolescent psychiatry patients with particularly elevated risk of psychosis. A wealth of epidemiological data has demonstrated numerous prognostic factors for psychosis in the general population (eg, sex, season of birth, immigrant background, non-psychotic mental disorders).^14–24^ Whether these factors are also prospectively associated with adult-onset psychotic disorders when applied within adolescent mental health services, where baseline psychosis risk is elevated, is unknown. This is an important clinical and research question for advancing psychosis prediction research.

We used linked register data on a total population birth cohort born in Finland between 1987 and 1992, followed up to a maximum age 30 years, to assess a range of clinical, service-use, and sociodemographic prognostic factors for adult-onset psychotic disorders (ie, onset after 18th birthday) in adolescent psychiatry patients (ie, individuals who were diagnosed with a mental disorder in specialist-level services in adolescence).

## Methods

We conducted a longitudinal, register-based total birth cohort study using data from the Nordic Children and Adolescents Born Preterm (NORDCAP) study, governed by the Finnish Institute for Health and Welfare. NORDCAP study data were linked from different national registers and cover all individuals born in Finland during years 1987-2016 (~1 800 000 individuals) followed from birth to December 31, 2016.

The current study used data recorded in the following registers: Medical Birth Register (for birth records, including place of birth, gestational age at birth, and birthweight); Care Register for Health Care (for diagnosis codes and dates, information on admissions, parental history of hospitalizations for mental disorders); Digital Population Data Services Agency (emigrations, deaths); Statistics Finland (parental socioeconomic status [SES] and education level). Data from registers were linked using anonymized personal identity codes. Diagnosis data from the Care Register for Health Care cover diagnoses assigned during secondary health care visits, covering all outpatient visits as well as inpatient admissions for the study period. These data have been used extensively in epidemiological research,^25^^,^^26^ and the recorded diagnoses have been found to identify mental disorders with high validity, including psychotic disorders.^27^^,^^28^

The current study and the NORDCAP project have been approved by the Institutional review board of the Finnish Institute for Health and Welfare (THL 4859/6.02.01/2023) and the relevant register keepers. According to Finnish legislation, informed consent is not required for the use of pseudonymized register data for research purposes. The study was conducted following the Strengthening the Reporting of Observational Studies in Epidemiology guidelines (see Supplementary material).

### Study Sample and Design

The initial sample comprised 384 507 individuals who were born in Finland in 1987-1992 (aged 25-30 years by the study end point on December 31, 2016) and did not have a recorded diagnosis of moderate to profound intellectual disability (International Classification of Diagnoses [ICD], version 10 codes F71.X-F79.X). We then limited the sample to adolescent psychiatry patients, defined as having a recorded diagnosis of any non-organic mental disorder in specialist-level services (ICD-10 codes: F1X, F3-F6X; F84.0, F84.1 & F84.5; F9X) between ages 13 and 17 years (inclusive). The dataset did not include individuals who attended services but did not receive a diagnosis. The final sample included individuals who, on their 18th birthday, were alive and living in Finland and did not have a recorded diagnosis of an outcome (psychotic) disorder. Individuals were followed from their 18th birthday until outcome diagnosis, death, emigration, or December 31, 2016, whichever occurred first (Figure S1).

### Outcome Disorders

We used primary and secondary diagnoses recorded in Care Register for Health Care to identify individuals with any non-organic psychotic disorder after 18th birthday. We also identified a subgroup of individuals diagnosed with schizophrenia-spectrum disorder after 18th birthday. Specific ICD-10 diagnoses considered as outcomes are listed in Table S1. We considered diagnoses assigned during both specialist outpatient visits and inpatient admissions.

### Prognostic Factors for Psychosis

#### Adolescent Mental Disorder Diagnoses

We considered the following mental disorder diagnoses (both primary and secondary) that were recorded in either outpatient or inpatient settings between age 13 and 17 years, grouped according to the categories of the mental disorder diagnoses in ICD-10:

- ***Substance use disorders*** (F1X Mental and behavioral disorders due to psychoactive substance use)
- ***Mood disorders*** (F3X Mood disorders)
- ***Anxiety disorders*** (F4X Neurotic, stress-related and somatoform disorders)
  ▪ ***Obsessive compulsive disorder** (F42.X)*
- ***Eating disorders*** (F5X Behavioral syndromes associated with physiological disturbances and physical factors)
- ***Personality disorders*** (F6X Disorders of adult personality and behavior; F21.X)
- ***Autism spectrum disorders*** (F84.0 Childhood autism, F84.1 Atypical autism, F84.5 Asperger syndrome)
- ***Childhood and adolescence onset disorders*** (F9X Behavioral and emotional disorders with onset usually occurring in childhood and adolescence)
  ▪ ***ADHD** (F90.X Hyperkinetic disorders)*
  ▪ ***Conduct disorders** (F91.X Conduct disorder, F92.X Mixed disorders of conduct and emotions)*
  ▪ ***Tic-disorders** (F95.X)*
  ▪ ***Other neurodevelopmental disorders** (F98.X Other behavioral and emotional disorders with onset usually occurring in childhood and adolescence)*

For each individual, we also calculated the total number of different recorded mental disorder diagnoses (on 3 cypher level) assigned between ages 13 and 17. Diagnoses with specification “Unspecified” (eg, “F32.9 Depressive episode, unspecified”) were not considered when calculating the total number of recorded diagnoses.

#### Sociodemographic Prognostic Factors

Sociodemographic prognostic factors considered included *sex* (recorded at birth) and *birth season* (winter [born in December–February]; spring [March–May]; summer [June–August]; autumn [September–November]). *Parents’ country of birth* (Finland; country other than Finland) was defined separately for mothers and fathers. *Parents’ highest attained education* was assessed for the year when the subject was born, again separately for mothers and fathers. This was categorized as either low (corresponding to International Standard Classification of Education version 4 classes 0-2), intermediate (classes 3-5), or high (classes 6-8). We also assessed occupation based SES of both mothers and fathers for the year when the subject was born. This was categorized as high, intermediate, low, or self-employed, using classification by Statistics Finland.

*Urbanicity of the birthplace* was defined following the Statistics Finland categorization of municipalities to urban, semi-rural, and rural (based on the proportion of population living in urban settlements and the population of the largest urban settlement).^29^  *Gestational age at birth* (in full weeks) was categorized as extremely preterm (<28 weeks), very preterm (28-31), late preterm (34-36), early term (37-38), term (39-41), post term (42-44). *Low birth weight* was defined as gestational age specific birthweight z-score (calculated using Maršál estimated fetal weight reference^30^^,^^31^) of less than −2. *Mother’s and father’s age* when the child was born was categorized as follows: <20 years, 20-24 years, 25-29 years, 30-34 years, 35-39 years, 40 years or older.

For sociodemographic prognostic factors that were associated with psychosis in univariate analyses, we investigated the risk of psychosis associated with accumulating number of prognostic factors (1, 2, 3, or 4+).

#### Clinical and Service-Use-Related Prognostic Factors

We used the Care Register for Health Care for records on *psychiatric inpatient admissions in adolescence* and to calculate *age at first presentation* to specialist psychiatric services in adolescence. We also identified individuals with adolescent psychiatry contacts whose *contact with specialist psychiatry services began in childhood*, defined as having recorded diagnosis of non-organic mental disorder in specialist level services between ages 0 and 12 years (ICD-10 codes: F1X, F3F6X; F84.0, F84.1 & F84.5; F9X; Finnish national modification of ICD-9 codes: 3150-3159, 3170-3199).

*Family history of serious mental disorder* was defined as having either mother or father with a Care Register for Health Care record indicating inpatient psychiatric treatment (as defined in Lahti et al,^32^  Table S2) by the time the subject turned 13 years. Similarly, the subject’s *family history of psychosis* was determined as having a parent with a recorded diagnosis of schizophrenia-spectrum disorder within the inpatient records by the time the subject turned 13 years old.

For clinical and service-use-related prognostic factors that were associated with psychosis in univariate analyses, we investigated the risk of psychosis associated with accumulating number of prognostic factors (1, 2, 3, or 4+).

### Statistical Analyses

Analyses were conducted using Stata version 18.0.^33^ We assessed the prevalence of different prognostic factors using frequencies and proportions. We calculated median (inter-quartile range) time from 18th birthday to outcome disorder. We describe the risk of outcome disorders from 18th birthday onwards with Kaplan–Meier cumulative risk estimates and 95% CIs. We used univariable and multivariable Cox regression models to assess the hazard of outcome disorders associated with the prognostic factors. Proportional hazards assumption was examined by testing of Schoenfeld residuals. Missing data were not imputed. For mental disorder diagnoses, sociodemographic prognostic factors, and clinical and service-use-related prognostic factors separately, we also investigated the univariable association between the total number of different prognostic factors (ie, the number of prognostic factors that the individual is characterized by), and the risk of outcome disorders from 18th birthday onwards.

We also report the sensitivity of the prognostic factors for identifying adulthood psychosis cases among adolescent psychiatry patients. Specifically, we calculated the proportion of psychosis cases occurring between ages 18 and 30 years that were preceded by each prognostic factor. For these analyses we limited the sample to adolescent psychiatry patients born in the year 1987 (*n* = 3815) to ensure sufficient and consistent follow-up period within the sample.

As sensitivity analyses, we examined the associations among males and females separately, and limited the outcome to schizophrenia-spectrum disorders.

## Results

### Sample Characteristics

The initial sample (*n* = 384 507) included 29 971 adolescent psychiatry patients (ie, individuals diagnosed with a mental disorder in specialist-level services in adolescence) (Figure S1). Of them, 7.6% (*n* = 2276) had been diagnosed with a psychotic disorder before age 18 and were excluded from further analyses. Individuals who had died or emigrated (*n* = 69) before age 18 were also excluded, resulting in an analytical sample of 27 626 adolescent psychiatry patients (61.0% females) followed for in total 229 111 person-years. Cumulative risk of psychosis between ages 18 and 30 years among adolescent psychiatry patients was 8.5% (95%CI, 8.0-9.0; 2003 psychosis cases). In comparison, cumulative risk of psychosis among individuals who were not adolescent psychiatry patients was 1.8% (95%CI, 1.7-1.9; 4882 psychosis cases) (Figure S1). The median (inter-quartile range) time from 18th birthday to first psychosis diagnosis among adolescent psychiatry patients was 3.0 (1.3-5.4) years, as compared to 4.6 (2.4-6.4) years among individuals who were not adolescent psychiatry patients. Sample characteristics are described in Table S3.

### Adolescent Mental Disorder Diagnoses and Psychosis Risk in Adulthood

The cumulative risk of psychosis associated with each adolescent mental disorder diagnosis category is described in Table 1. Cumulative risk of psychosis was elevated across all adolescent mental disorder diagnoses, ranging from 7.9% (4.3%-14.2%) for Tic disorders to 12.2% (9.9%-15.1%) for Personality disorders (Figures S2-S14).

**Table 1:** Prognostic Factors and Cumulative Risk of Psychosis in Adulthood Among Adolescent Psychiatry Patients (Prognostic Factor Categories: Mental Disorder Diagnoses Assigned in Adolescence; Sociodemographic Prognostic Factors; Clinical and Service-Use-Related Prognostic Factors)

**Prognostic factors**			***N***	**%**	**% Risk of psychotic disorder** ^**a**^		**HR (95% CI)** ^**b**^		**HR (95% CI), adjusted** ^**c**^ ^**,**^ ^**e**^	
**Mental disorder diagnoses in adolescence**										
Substance use disorders										
		No	24 636	89.2	8.4	(7.8-8.9)	Ref.		Ref.	
		Yes	2990	10.8	9.7	(8.6-11.0)	**1.21**	**(1.06-1.38)**	**1.52**	**(1.33-1.74)**
Mood disorders										
		No	16 898	61.2	7.0	(6.4-7.7)	Ref.		Ref.	
		Yes	10 728	38.8	10.9	(10.2-11.6)	**1.73**	**(1.58-1.89)** ^**d**^	**1.98**	**(1.81-2.16)**
Anxiety disorders										
		No	17 738	64.2	7.9	(7.5-8.4)	Ref.		Ref.	
		Yes	9888	35.8	9.5	(8.5-10.6)	**1.19**	**(1.09-1.30)** ^**d**^	**1.46**	**(1.33-1.60)**
	Obsessive-compulsive disorder									
		No	26 930	97.5	8.4	(8.0-8.9)	Ref.			
		Yes	696	2.5	10.9	(8.6-13.8)	**1.44**	**(1.13-1.83)**	-	-
Eating disorders										
		No	24 201	87.6	8.6	(8.0-9.0)	Ref.		Ref.	
		Yes	3425	12.4	8.5	(7.3-9.9)	1.02	(0.90-1.17)^**d**^	**1.27**	**(1.11-1.45)**
Personality disorders										
		No	26 838	97.1	8.4	(7.9-8.9)	Ref.		Ref.	
		Yes	788	2.9	12.2	(9.9-15.1)	**1.57**	**(1.26-1.95)** ^**d**^	**1.56**	**(1.26-1.94)**
Autism spectrum disorders										
		No	26 705	96.7	8.4	(7.9-8.9)	Ref.		Ref.	
		Yes	921	3.3	11.4	(8.5-15.2)	1.21	(0.97-1.52)	**1.73**	**(1.38-2.18)**
Childhood and adolescence onset disorders										
		No	17 331	62.7	7.7	(7.3-8.3)	Ref.		Ref.	
		Yes	10 295	37.3	9.8	(8.9-10.9)	**1.22**	**(1.11-1.33)**	**1.48**	**(1.35-1.63)**
	ADHD									
		No	25 957	94.0	8.5	(8.0-9.0)	Ref.			
		Yes	1669	6.0	8.4	(6.7-10.5)	0.88	(0.73-1.08)^**d**^	-	-
	Conduct disorders									
		No	23 233	84.1	8.1	(7.6-8.7)	Ref.			
		Yes	4393	15.9	10.5	(9.5-11.6)	**1.37**	**(1.23-1.53)** ^**d**^	-	-
	Tic disorders									
		No	27 309	98.9	8.5	(8.0-9.0)	Ref.			
		Yes	317	1.1	7.9	(4.3-14.2)	0.72	(0.45-1.16)^**d**^	-	-
	Other Neurodevelopmental disorders									
		No	26 857	97.2	8.4	(7.9-8.9)	Ref.			
		Yes	769	2.8	10.9	(8.7-13.6)	**1.37**	**(1.09-1.73)**	-	-
**Sociodemographic prognostic factors**										
Sex										
		Female	16 839	61.0	7.9	(7.2-8.5)	Ref.		Ref.	
		Male	10 787	39.0	9.6	(9.0-10.3)	**1.25**	**(1.15-1.37)** ^**d**^	**1.20**	**(1.09-1.32)**
Birth season										
		Winter	6434	23.3	8.6	(7.7-9.5)	Ref.		Ref.	
		Spring	7186	26.0	8.2	(7.5-9.1)	0.97	(0.85-1.10)	0.94	(0.82-1.08)
		Summer	7172	26.0	8.3	(7.6-9.1)	0.97	(0.86-1.10)	0.93	(0.81-1.07)
		Autumn	6834	24.7	8.7	(7.9-9.6)	1.00	(0.88-1.14)	1.00	(0.87-1.15)
Father’s birth country										
		Finland	26 274	97.6	8.4	(7.9-8.9)	Ref.		Ref.	
		Not in Finland	640	2.4	9.5	(7.2-12.4)	1.22	(0.93-1.6)	1.11	(0.76-1.61)
Mother’s birth country										
		Finland	26 915	98.3	8.5	(8.0-9.0)	Ref.		Ref.	
		Not in Finland	478	1.7	7.7	(5.5-10.8)	1.02	(0.73-1.44)	0.96	(0.61-1.51)
Father’s education level										
		Low	7958	29.6	8.2	(7.5-8.9)	Ref.		Ref.	
		Middle	15 623	58.1	8.5	(7.8-9.3)	1.03	(0.93-1.13)	1.07	(0.95-1.20)
		High	3297	12.3	8.5	(7.3-9.9)	1.01	(0.87-1.18)	1.14	(0.92-1.41)
Mother’s education level										
		Low	7789	28.4	8.5	(7.7-9.3)	Ref.		Ref.	
		Middle	17 010	62.1	8.5	(7.9-9.2)	0.99	(0.89-1.09)	1.04	(0.92-1.17)
		High	2594	9.5	8.0	(6.6-9.7)	0.89	(0.75-1.05)	1.05	(0.83-1.33)
Father’s SES (occupation based)										
		High	3597	14.2	8.6	(7.4-10.0)	Ref.		Ref.	
		Intermediate	4195	16.6	8.0	(7.1-9.0)	0.98	(0.83-1.15)	1.01	(0.82-1.25)
		Self-employed	2356	9.3	7.2	(6.0-8.7)	0.82	(0.67-1.01)	0.89	(0.68-1.15)
		Low	15 130	59.9	8.6	(7.9-9.4)	1.00	(0.87-1.14)	1.01	(0.83-1.23)
Mother’s SES (occupation based)										
		High	2891	11.3	7.4	(6.1-9.1)	Ref.		Ref.	
		Intermediate	10 206	39.7	8.2	(7.6-8.9)	1.17	(0.99-1.38)	1.24	(1.00-1.54)
		Self-employed	1290	5.0	7.5	(5.8-9.7)	1.01	(0.77-1.32)	1.24	(0.90-1.70)
		Low	11 300	44.0	9.0	(8.2-10.0)	**1.26**	**(1.07-1.48)**	**1.35**	**(1.08-1.68)**
Urbanicity of the birthplace										
		Urban	17 391	63.0	8.7	(8.2-9.2)	Ref.		Ref.	
		Semi-rural	3900	14.1	9.0	(7.9-10.2)	1.02	(0.90-1.16)	1.06	(0.93-1.21)
		Rural	6298	22.8	7.7	(6.5-9.1)	**0.81**	**(0.73-0.91)**	**0.82**	**(0.73-0.93)**
Low birth weight										
		No	26 953	98.9	8.5	(8.0-9.0)	Ref.		Ref.	
		Yes	307	1.1	9.4	(6.2-14.1)	**1.12**	**(0.76-1.66)**	0.81	(0.48-1.34)
Gestational age at birth (weeks)										
		Extremely preterm (−27)	58	0.2	16.9	(9.1-30.2)	**2.25**	**(1.17-4.33)**	**2.19**	**(1.04-4.61)**
		Very preterm (28-31)	154	0.6	13.7	(8.6-21.3)	**1.62**	**(1.02-2.58)**	1.64	(0.99-2.73)
		Moderately preterm (32-33)	174	0.6	5.2	(2.7-9.7)	0.73	(0.38-1.4)	0.70	(0.33-1.47)
		Late preterm (34-36)	1111	4.1	8.3	(6.5-10.5)	1.00	(0.8-1.25)	1.06	(0.83-1.35)
		Early term (37-38)	4860	17.8	8.4	(7.5-9.4)	0.99	(0.88-1.12)	1.03	(0.91-1.17)
		Term (39-41)	19 678	72.0	8.6	(8.0-9.2)	Ref.		Ref.	
		Post term (42-44)	1286	4.7	7.9	(6.5-9.7)	1.02	(0.83-1.25)	1.00	(0.79-1.26)
										
Maternal age (years)										
		<20	1376	5.0	12.9	(9.5-17.3)	Ref.		Ref.	
		20-24	6316	22.9	7.9	(7.2-8.6)	**0.70**	**(0.58-0.85)**	**0.69**	**(0.53-0.88)**
		25-29	9472	34.3	7.9	(7.3-8.6)	**0.68**	**(0.57-0.82)**	**0.67**	**(0.51-0.88)**
		30-34	6729	24.4	8.9	(8.0-9.9)	**0.74**	**(0.62-0.90)**	**0.70**	**(0.53-0.93)**
		35-39	2984	10.8	8.6	(7.5-10)	**0.73**	**(0.59-0.90)**	**0.70**	**(0.51-0.96)**
		40-	749	2.7	7.4	(5.5-9.8)	**0.65**	**(0.47-0.90)**	**0.65**	**(0.43-0.98)**
Paternal age (years)										
		<20	372	1.4	9.8	(6.8-14.0)	Ref.		Ref.	
		20-24	3732	13.9	9.5	(7.6-11.7)	0.92	(0.63-1.33)	1.22	(0.76-1.94)
		25-29	8362	31.1	8.0	(7.3-8.8)	0.84	(0.59-1.21)	1.21	(0.75-1.94)
		30-34	7908	29.4	8.6	(7.8-9.4)	0.88	(0.62-1.27)	1.28	(0.79-2.08)
		35-39	4147	15.4	8.2	(7.3-9.3)	0.85	(0.58-1.23)	1.29	(0.79-2.12)
		40-	2394	8.9	7.9	(6.7-9.3)	0.83	(0.57-1.22)	1.28	(0.76-2.14)
**Clinical and service-use-related prognostic factors**										
Age at first adolescent psychiatry contact (years)										
		13	6749	24.4	8.6	(7.7-9.6)	Ref.		Ref.	
		14	5264	19.1	7.2	(6.4-8.0)	0.88	(0.77-1.02)	0.96	(0.83-1.11)
		15	5425	19.6	8.5	(7.6-9.5)	1.01	(0.89-1.16)	**1.17**	**(1.01-1.35)**
		16	5237	19.0	7.9	(7.1-8.8)	0.99	(0.86-1.13)	**1.24**	**(1.07-1.44)**
		17	4951	17.9	10.4	(8.7-12.4)	**1.25**	**(1.09-1.42)** ^**d**^	**1.64**	**(1.42-1.90)**
Child psychiatry contact										
		No	22 306	80.7	8.2	(7.7-8.7)	Ref.		Ref.	
		Yes	5320	19.3	10.0	(8.8-11.3)	**1.16**	**(1.04-1.29)**	**1.28**	**(1.13-1.45)**
Psychiatric inpatient admission in adolescence										
		No	20 099	72.8	7.0	(6.5-7.6)	Ref.		Ref.	
		Yes	7527	27.2	12.5	(11.6-13.4)	**1.98**	**(1.81-2.16)** ^**d**^	**2.08**	**(1.90-2.27)**
Family history of psychosis										
		No	26 887	97.3	8.3	(7.9-8.8)	Ref.		Ref.	
		Yes	739	2.7	14.5	(11.7-17.9)	**1.82**	**(1.47-2.24)**	**1.46**	**(1.16-1.83)**
Family history of serious mental illness										
		No	22 248	80.5	8.0	(7.4-8.5)	Ref.		Ref.	
		Yes	5378	19.5	10.8	(9.8-11.8)	**1.42**	**(1.28-1.57)**	**1.26**	**(1.13-1.40)**

Abbreviation: HR = hazard ratio. Bold font indicates statistically significant association (p < 0.05).

a Cumulative risk by the end of the follow-up.

b Univariable Cox regression models; only including one prognostic factor at a time.

c Multivariable Cox regression model; including all prognostic factors in the prognostic factor category (in the prognostic factor category “Mental disorder diagnoses,” the subcategory diagnoses are not included in the model).

d Statistically significant non-proportionality of hazards was observed for the univariable Cox regression model. The estimate should be interpreted as the average effect over the follow-up period. For cumulative risk curves, please see Supplementary materials.

e Statistically significant non-proportionality of hazards was observed for the following multivariable Cox regression models: model including all mental disorder diagnoses, model including all clinical and service-use-related prognostic factors. The estimates should be interpreted as the average effect over the follow-up period.

**Figure 1:**
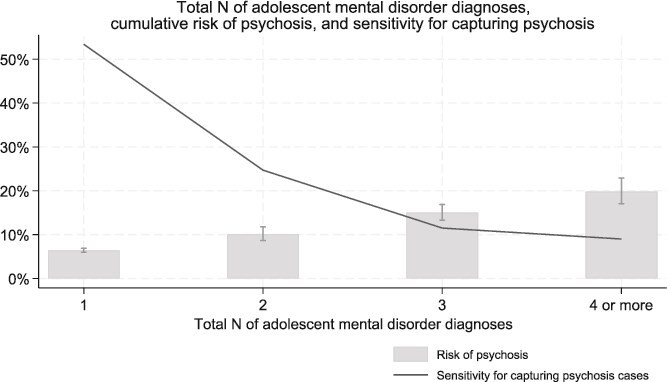
Total Number of Different Mental Disorder Diagnoses Assigned in Adolescence, Cumulative Risk of Adult-Onset Psychosis and Sensitivity for Capturing Psychosis Among Adolescent Psychiatry Patients (ie, Proportion of Psychosis Cases That Were Preceded by Certain N of Different Adolescent Mental Disorders).

The total number of different adolescent mental disorder diagnoses was associated with later psychosis, and the cumulative risk of psychosis increased with the number of adolescent diagnoses (Table 2). The cumulative risk of psychotic disorder was 6.5% (6.0%-6.9%) for individuals diagnosed with one mental disorder by age 18, compared to 10.1% (8.6%-11.8%) for 2 diagnoses, 15.0% (13.3%-16.9%) for 3 diagnoses and 19.8% (17.1%-22.9%) for individuals with 4 or more different mental disorder diagnoses by age 18 (Table 2, Figure 1, Figure S15).

**Table 2:** Total Number of Different Adolescent Mental Disorder Diagnoses, Cumulative Risk of Psychosis, and Sensitivity for Capturing Psychosis Cases (ie, Proportion of Psychosis Cases That Were Preceded by Total *N* of Different Adolescent Mental Disorders)

					**Risk of psychosis**				**Sensitivity for capturing psychosis % (*n*)** ^**c**^	
**Total *N* of diagnoses**			***N***	**%**	**% Cumulative risk of psychosis** ^**a**^ **(95%CI)**		**HR (95%CI)** ^**b**^			
Any									100 (279)
									
1			17 866	64.7	6.5	(6.0-6.9)	Ref.		54.8 (153)
2			6317	22.9	10.1	(8.6-11.8)	**1.54**	**(1.38-1.71)**	24.7 (69)
3			2254	8.2	15.0	(13.3-16.9)	**2.52**	**(2.21-2.87)**	11.5 (32)
4 or more			1189	4.3	19.8	(17.1-22.9)	**3.41**	**(2.93-3.97)**	9.0 (25)

Abbreviation: HR: hazard ratio. Bold font indicates statistically significant association (p < 0.05).

For the analyses on sensitivity for capturing psychosis cases, the sample is restricted to individuals born in year 1987, excluding individuals who died or emigrated before the end of the follow-up, or who were diagnosed with psychosis before age 18.

Cochran–Armitage test for trend *P* < .001.

Departure from linear trend *P* < .05.

a Cumulative risk by the end of the follow-up

b Statistically significant non-proportionality of hazards was observed. The estimates should be interpreted as the average effect over the follow-up period. For cumulative risk curves, please see Figure S15.

c Total *N* of psychosis cases occurring in adolescent psychiatry patients = 279.

Sensitivity of specific adolescent psychiatry diagnoses for capturing adulthood psychosis is described in Table S4.

### Sociodemographic Prognostic Factors and Psychosis Risk in Adulthood

**Figure 2:**
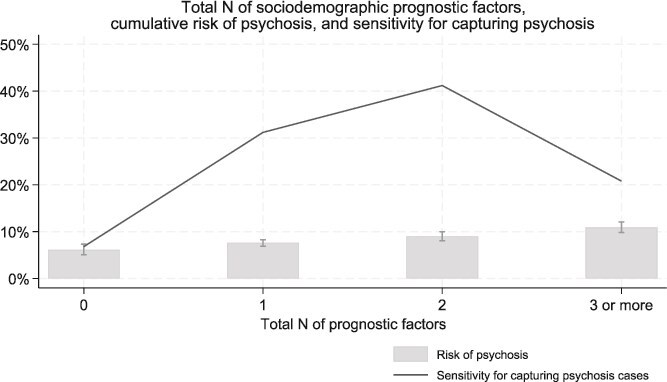
Total number of sociodemographic prognostic factors, cumulative risk of adult-onset psychosis and sensitivity for capturing psychosis among adolescent psychiatry patients (ie, proportion of psychosis cases that were preceded by certain total *N* of prognostic factors). Prognostic factors considered: male sex; mother’s low SES; born in an urban or semi-rural area; mother aged <20 years; low birth weight; extreme or very preterm birth.

The cumulative risk of psychosis was widely dispersed across the levels of sociodemographic prognostic factors for psychosis (Table 1 and Figures S16-S28). The highest cumulative risk was observed for maternal age <20 years (12.9%; 9.5%-17.3%) and extremely preterm and very preterm birth (16.9% [9.1%-30.2%] and 13.7% [8.6%-21.3%], respectively). There was a linear association between total number of different sociodemographic prognostic factors and psychosis risk (Table S5, Figure 2, Figure S29). The sensitivity of sociodemographic prognostic factors for capturing adulthood psychosis is described in Table S6.

### Clinical and Service-Use-Related Prognostic Factors and Psychosis Risk in Adulthood

The cumulative risk of psychosis associated with different clinical and service-use-related psychosis prognostic factors is described in Table 1. All clinical and service-use-related factors were associated with an increased hazard of psychosis, with cumulative risk ranging from 10.4% (8.7%-12.4%) among those who first presented to adolescent psychiatry services when aged 17 years, to risk a of 14.5% (11.7%-17.9%) associated with having family history of psychotic disorder (Figures S30-S34).

**Figure 3:**
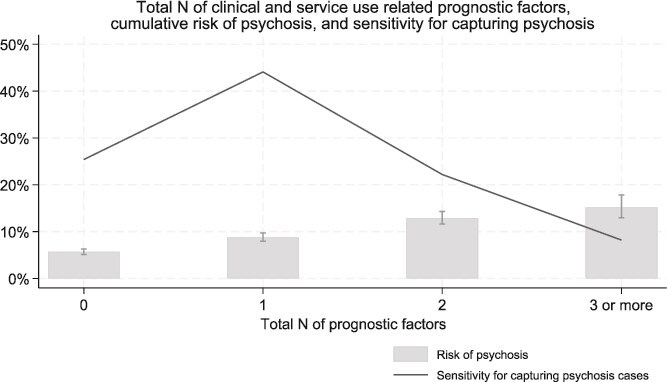
Total number of clinical and service-use-related prognostic factors, cumulative risk of adult-onset psychosis and sensitivity for capturing psychosis among adolescent psychiatry patients (ie, proportion of psychosis cases that were preceded by certain total *N* of prognostic factors). Prognostic factors considered: attended adolescent psychiatry services first time when aged 17 years; child psychiatry contact; adolescent psychiatry inpatient admission; parental history of psychosis; parental history of serious mental disorder.

There was an association between total number of different clinical and service-use-related prognostic factors and later psychosis, with cumulative risk ranging from 5.7% (5.1%-6.3%) for adolescent psychiatry patients with none of the prognostic factors, to 15.2% (12.9%-17.8%) for individuals with 3 or more of the prognostic factors. (Table S7, Figure 3, Figure S35).

Sensitivity of clinical and service-use-related prognostic factors for capturing adulthood psychosis is described in Table S8

### Sensitivity Analyses

Formal testing of Schoenfeld residuals suggested statistically significant non-proportionality of hazards in models indicated in Tables 1 and 2, and Table S7, and the estimates should be interpreted as the average effect over the study period. Natural incidence of cases across the study groups over the study period (risk curves) are presented in Figures S3-S35; visual inspection suggested weakening of the effect over time for some factors (eg, anxiety disorders), whereas strengthening of the effect was observed for other factors (eg, conduct disorders).

As compared to the main analyses, the analyses using an outcome of schizophrenia-spectrum disorder yielded similar results. These results are included in the Supplementary material. Results from analyses stratified by sex are also included in the Supplementary material.

## Discussion

Up to half of all psychotic disorders diagnosed in the population emerge in individuals who had attended adolescent psychiatry services,^12^^,^^13^ highlighting important opportunities for psychosis prediction and prevention within this clinical population. With this in mind, we assessed, for the first time, a range of potential prognostic factors for adult-onset psychosis in a total birth cohort comprised of all individuals born in Finland in 1987-1992 who were diagnosed with a mental disorder in specialist-level services in adolescence (adolescent psychiatry patients). In keeping with previous research,^12^ adolescent psychiatry patients had a substantial risk of being diagnosed with a psychotic disorder in adulthood: between ages 18 and 30, the cumulative risk of psychosis was 9%.

We found that the cumulative risk of psychosis was widely dispersed across adolescent mental disorder categories, ranging from 8% for adolescents diagnosed with a tic disorder to 12% for adolescents diagnosed with a personality disorder. Risk for psychosis increased, however, in line with the overall number of diagnoses accumulated over the course of adolescence. Of all individuals who received, by age 18, a single mental disorder diagnosis, 7% went on to be diagnosed with a psychotic disorder by age 30. For individuals who had, by age 18, received 2, 3, or (at least) 4 mental disorder diagnoses, on the other hand, the risk of later psychotic disorder increased to 10%, 15%, and 20%, respectively. As with all prediction-related research, there is an inevitable trade-off between sensitivity and precision. While the presence of 2 or more mental disorders can identify subgroups at particularly elevated risk, it is also the case that following up only these groups will reduce the sensitivity of the approach.

An increased risk of psychotic disorder was also associated with inpatient psychiatric admission in adolescence. In total, 13% of all adolescents with an inpatient admission went on to be diagnosed with a psychotic disorder between ages 18 and 30. Age of presentation to psychiatric services was also associated with psychosis risk. First presentation at ages 15, 16, or 17 was associated with a small but significantly higher risk of psychosis compared to first presentation at ages 13 or 14, potentially reflecting factors such as barriers to timely access to care for some high-risk individuals, and sharp increase in specific risk factors such as substance use in late-adolescence. Conversely, individuals who had a history of also presenting to child psychiatry services (ages 0-12) in addition to their adolescent psychiatry presentation had an increased psychosis risk compared to adolescent psychiatry patients who had not presented to child psychiatry services.

Family history was also a prognostic factor for later psychosis: both parental history of psychosis and parental history of inpatient psychiatric admission for any reason were independently associated with later psychotic disorder in adolescent psychiatry patients.

A number of sociodemographic factors were also significant markers of increased psychosis risk, including male sex, urban (vs rural) place of birth, and maternal (but not paternal) SES, young maternal age (defined as age < 20 when the child was born), being born very or extremely prematurely (before week 32 of pregnancy), as well as low weight for gestational age at birth.

Interestingly, a number of prognostic factors that have been shown to be associated with psychosis at a population level were not associated with adulthood psychosis among adolescent psychiatry patients. This included season of birth,^17^ parental education,^14–16^ immigration,^19^ and father’s age.^20^ Conversely, we observed significant associations for factors that were not found to be associated with psychosis among youth at ultra-high risk for psychosis.^34^ This highlights that prognostic factors derived from general population research or from other clinical samples do not necessarily apply to adolescent psychiatry patients. While methodological differences prevent direct comparisons, our findings are consistent with previous studies that have suggested that the risk of developing a psychotic disorder is not captured by any single non-psychotic mental disorder.^22–24^

This is the first nationwide, total birth cohort study to examine the extent to which known prognostic factors for psychosis are associated with later psychosis among adolescent psychiatry patients. The findings of this study help us to better understand the high psychosis risk within this clinical population, and will inform future studies on the development of risk prediction models, demonstrating variables that might be useful for such models. The results can also inform studies aiming to identify underlying mechanisms that may explain or influence the observed associations.

Further research will also be necessary to investigate what interventions may impact on the risk of adult-onset psychosis within adolescent psychiatry cohorts. One recent study used quasi-experimental methods to investigate whether antidepressant medication treatment in adolescent psychiatry patients impacted on subsequent psychosis risk.^35^ This so-called “intervention as prevention” hypothesis was not supported, with antidepressant treatment not demonstrating a causal effect on the risk of later psychotic disorders. Research on anti-inflammatory treatments in adolescent psychiatry patients, on the other hand, suggests a potential protective effect of the antibiotic medication, doxycycline. In an emulated target trial of adolescent psychiatry patients treated with doxycycline compared to other (non-doxycycline) antibiotics, the doxycycline-exposed group had a significantly lower risk of going on to be diagnosed with schizophrenia.^36^

Strengths of the study include the long follow-up period, covering the age of peak onset of psychotic disorders, and avoiding the attrition that usually occurs in typical cohort studies. Moreover, the coverage of the dataset is excellent, including all individuals born in Finland years 1987-1992, and covering information on prognostic factors from birth to age 18 years. However, it is worth noting that the results might not directly generalize to other settings, eg, to people born in other countries who subsequently move to Finland, or to other regions and birth cohorts. The study focused on adult-onset psychosis, enabling consideration of prognostic factors during the entire adolescent age-period, and the extent to which the findings generalize to psychosis cases occurring before age 18 is unknown. While we do not have a reason to believe that these prognostic factors would not also apply to early-onset psychosis, further research specific to psychosis onset age <18 will be valuable to confirm or refute this. In addition, unlike many other studies examining parental characteristics in relation to psychosis risk, we were able to use sociodemographic data on fathers as well as mothers. It is worth noting that non-proportionality of hazards was observed for some of the Cox regression models, and the hazard ratio estimates from these models should be interpreted as the average effect over the follow-up. For these models, the assessment of the risk curves showing the natural incidence of cases across the study groups can be particularly informative.

As we used real-world patient records, our approach has high ecological validity in terms of its application to mental health services. The register-based diagnoses have been found to capture psychotic disorders with high validity.^25–28^ These studies have been based on inpatient diagnoses, however, so the extent to which this applies to outpatient diagnoses is less certain. A large majority of individuals diagnosed with psychosis in Finland, however, have an inpatient admission.^37^ Finally, our approach, based on utilization of adolescent psychiatry services, captures a greater proportion of females who go on to develop psychosis than males. This is in keeping with the fact that more females present to child and adolescent psychiatry services.

## Conclusions

We evaluated a wide range of clinical, service use, and sociodemographic variables as potential prognostic factors for adult-onset psychotic disorders within general adolescent psychiatry clinics, a group known to be at elevated risk of psychosis. Our findings demonstrate that routinely collected data can help differentiate levels of psychosis risk within real-world adolescent psychiatry services, and they can inform future studies, including development of formal prediction models. Our findings underscore the value of integrating clinical, service use, and sociodemographic factors alongside other potential markers of psychosis risk, such as neurocognitive, neuroimaging, genetic, proteomic, behavioral, and digital phenotyping data. This comprehensive approach could enhance early identification of psychosis risk within real-world adolescent psychiatry services.

## Supplementary Material

Supplementary_materials_sbag031
