# Supplementary material for: Prognostic Factors for Adulthood Psychosis in Adolescent Psychiatry Services: A Longitudinal Total Birth Cohort Study
Source: Schizophr Bull. 2026 Jun 4;52(3):sbag031. doi: 10.1093/schbul/sbag031 (PMC13235725; doi:10.1093/schbul/sbag031)
Supplement: Supplementary_materials_sbag031 [file supplementary_materials_sbag031.zip › Supplementary_materials_sbag031.pdf]

Supplementary Materials

Title: 'Prognostic factors for adulthood psychosis in adolescent psychiatry services: a longitudinal total birth cohort study'

SUPPELEMENTARY TABLES ---------- 3

Table S1. Outcome ICD-10 diagnoses---------- 3

Table S2. Diagnosis codes used to identify parents with a history of a) any serious mental disorder and b) schizophrenia-spectrum disorder. ---------- 4

Table S3. Sample characteristics---------- 5

Table S4. Proportion of psychosis cases that were preceded by different adolescent mental disorders (sensitivity). -- 8

Table S5. Total number of different sociodemographic prognostic factors, cumulative risk of psychosis and sensitivity for capturing psychosis---------- 9

Table S6. Proportion of psychosis cases that were preceded by different sociodemographic prognostic factors (sensitivity). ----------10

Table S7. Total number of different clinical and service use related prognostic factors, cumulative risk of psychosis and sensitivity for capturing psychosis----------11

Table S8. Proportion of psychosis cases that were preceded by different clinical and service use related prognostic factors (sensitivity). ----------12

Table 9. Sensitivity analysis: Prognostic factors and cumulative risk of psychosis in adulthood among FEMALE adolescent psychiatry patients. ----------13

Table 10. Sensitivity analysis: Prognostic factors and cumulative risk of psychosis in adulthood among MALE adolescent psychiatry patients. ----------15

Table 11. Sensitivity analysis: Prognostic factors and cumulative risk of schizophrenia-spectrum psychosis in adulthood among adolescent psychiatry patients. ----------17

SUPPLEMENTARY FIGURES ----------19

Figure S1. Study flowchart----------19

Figure S2. Cumulative risk of psychosis in adulthood in individuals with vs. without contact with adolescent psychiatry services ----------20

Figure S3. Cumulative risk of psychosis among individuals with and without an adolescent diagnosis of Substance use disorders. ----------21

Figure S4. Cumulative risk of psychosis among individuals with and without an adolescent diagnosis of Mood disorders. ----------22

Figure S5. Cumulative risk of psychosis among individuals with and without an adolescent diagnosis of Anxiety disorders. ----------23

Figure S6. Cumulative risk of psychosis among individuals with and without an adolescent diagnosis of Eating disorders. ----------24

Figure S7. Cumulative risk of psychosis among individuals with and without an adolescent diagnosis of Personality disorders. ----------25

Figure S8. Cumulative risk of psychosis among individuals with and without an adolescent diagnosis of Autism spectrum disorders.----------26

Figure S9. Cumulative risk of psychosis among individuals with and without an adolescent diagnosis of Childhood and adolescence onset disorders. ----------27

Figure S10. Cumulative risk of psychosis among individuals with and without an adolescent diagnosis of OCD. ------28

Figure S11. Cumulative risk of psychosis among individuals with and without an adolescent diagnosis of ADHD. ----29

Figure S12. Cumulative risk of psychosis among individuals with and without an adolescent diagnosis of Conduct disorders. ----30

Figure S13. Cumulative risk of psychosis among individuals with and without an adolescent diagnosis of Tic disorders. ----31

Figure S14. Cumulative risk of psychosis among individuals with and without an adolescent diagnosis of Other neurodevelopmental disorders. ----32

Figure S15. **Total number of different mental disorder diagnoses assigned in adolescence and cumulative risk of psychosis in adulthood. ----33**

Figure S16. Cumulative risk of psychosis among male and female adolescent psychiatry patients. ----34

Figure S17. Cumulative risk of psychosis among adolescent psychiatry patients by birth season. ----35

Figure S18. Cumulative risk of psychosis among adolescent psychiatry patients by father's birth country. ----36

Figure S19. Cumulative risk of psychosis among adolescent psychiatry patients by mother's birth country. ----37

Figure S20. Cumulative risk of psychosis among adolescent psychiatry patients by father's education level. ----38

Figure S21. Cumulative risk of psychosis among adolescent psychiatry patients by mother's education level. ----39

Figure S22. Cumulative risk of psychosis among adolescent psychiatry patients by father's SES. ----40

Figure S23. Cumulative risk of psychosis among adolescent psychiatry patients by mother's SES. ----41

Figure S24. Cumulative risk of psychosis among adolescent psychiatry patients by urbanicity of the birth municipality. ----42

Figure S25. Cumulative risk of psychosis among adolescent psychiatry patients by birth weight (low vs. normal/high for gestational age). ----43

Figure S26. Cumulative risk of psychosis among adolescent psychiatry patients by gestational age at birth (full weeks) ----44

Figure S27. Cumulative risk of psychosis among adolescent psychiatry patients by maternal age (years, when the child was born). ----45

Figure S28. Cumulative risk of psychosis among adolescent psychiatry patients by paternal age (years, when the child was born) ----46

Figure S29. Total number of different sociodemographic prognostic factors and risk of psychosis in adulthood ----47

Figure S30. Cumulative risk of psychosis among adolescent psychiatry patients by age at first adolescent psychiatry contact. ----48

Figure S31. Cumulative risk of psychosis among adolescent psychiatry patients by child psychiatry contact. ----49

Figure S32. Cumulative risk of psychosis among adolescent psychiatry patients by having a psychiatric inpatient admission in adolescence. ----50

Figure S33. Cumulative risk of psychosis among adolescent psychiatry patients by parental history of serious mental disorder. ----51

Figure S34. Cumulative risk of psychosis among adolescent psychiatry patients by parental history of psychotic disorder. ----52

Figure S35. Total number of different clinical and service use related prognostic factors and risk of psychosis in adulthood ----53

SUPPELEMENTARY TABLES

Table S1. Outcome ICD-10 diagnoses

**Any psychotic disorder**			**Schizophrenia-spectrum disorder**	
F20.X *(excluding F20.4)*	Schizophrenia		F20.X *(excluding F20.4)*	Schizophrenia
F22.X	Persistent delusional disorders		F22.X	Persistent delusional disorders
F23.X	Acute and transient psychotic disorders		F23.X	Acute and transient psychotic disorders
F24.X	Induced delusional disorder		F24.X	Induced delusional disorder
F25.X	Schizoaffective disorders		F25.X	Schizoaffective disorders
F28.X	Other nonorganic psychotic disorders		F28.X	Other nonorganic psychotic disorders
F29.X	Unspecified nonorganic psychosis		F29.X	Unspecified nonorganic psychosis
F1X.5	Substance use induced psychosis		F1X.5	Substance use induced psychosis
F30.2	Mania with psychotic symptoms			
F31.2	Bipolar affective disorder, current episode manic with psychotic symptoms			
F31.5	Bipolar affective disorder, current episode severe depression with psychotic symptoms			
F32.3	Severe depressive episode with psychotic symptoms			
F33.3	Recurrent depressive disorder, current episode severe with psychotic symptoms			

Table S2. Diagnosis codes used to identify parents with a history of a) any serious mental disorder and b) schizophrenia-spectrum psychosis.

**Diagnosis classification system**		**Any serious mental disorder**		**Schizophrenia-spectrum disorder**
ICD-8		291, 295, 296–305, 306.4–306.5, 306.8, 306.98, 307; E95		295, 297, 298.10-299.99
ICD-9		291–292, 295–298, 300–304, 305, 3071A, 3074, 3075A–3075B, 3078A, 3079X, 3090A, 3092C–3099X, 312; E95		295, 297-298
ICD-10		F1–F6, R45.8, X60–X84; Y87.0		F20-F29

Table S3. Sample characteristics

Mental disorder diagnoses in adolescence		Total N	Total %	Diagnosis of psychotic disorder in adulthood: No N	No %	Yes N	Yes %
Substance use disorders							
	No	24,636	89.2 %	22,888	89.3 %	1,748	87.3 %
	Yes	2,990	10.8 %	2,735	10.7 %	255	12.7 %
Mood disorders							
	No	16,898	61.2 %	15,928	62.2 %	970	48.4 %
	Yes	10,728	38.8 %	9,695	37.8 %	1,033	51.6 %
Anxiety disorders							
	No	17,738	32.5 %	16,526	32.6 %	1,212	30.8 %
	Yes	9,888	18.1 %	9,097	18.0 %	791	20.1 %
Obsessive-compulsive disorder							
	No	26,930	49.4 %	24,996	49.4 %	1,934	49.1 %
	Yes	696	1.3 %	627	1.3 %	69	1.8 %
Eating disorders							
	No	24,201	87.6 %	22,450	87.6 %	1,751	87.4 %
	Yes	3,425	12.4 %	3,173	12.4 %	252	12.6 %
Personality disorders							
	No	26,838	49.4 %	24,921	49.4 %	1,917	48.8 %
	Yes	788	1.5 %	702	1.4 %	86	2.2 %
Autism spectrum disorders							
	No	26,705	59.4 %	24,780	59.3 %	1,925	60.7 %
	Yes	921	2.0 %	843	2.0 %	78	2.5 %
Childhood and adolescence onset disorders							
	No	17,331	32.3 %	16,162	32.5 %	1,169	30.0 %
	Yes	10,295	19.2 %	9,461	19.0 %	834	21.4 %
ADHD							
	No	25,957	51.0 %	24,059	50.9 %	1,898	52.8 %
	Yes	1,669	3.3 %	1,564	3.3 %	105	2.9 %
Conduct disorders							
	No	23,233	42.3 %	21,640	42.5 %	1,593	39.9 %
	Yes	4,393	8.0 %	3,983	7.8 %	410	10.3 %
Tic disorders							
	No	27,309	50.1 %	25,323	50.1 %	1,986	50.5 %
	Yes	317	0.6 %	300	0.6 %	17	0.4 %
Other Neurodevelopmental disorders							
	No	26,857	97.2 %	24,931	97.3 %	1,926	96.2 %
	Yes	769	2.8 %	692	2.7 %	77	3.8 %

Note: Missing data reported only for variables with missing data.

Table S3 (continued). Sample characteristics

Sociodemographic prognostic factors		Total		Diagnosis of psychotic disorder in adulthood			
				No		Yes	
		N	%	N	%	N	%
Sex							
	Female	16,839	61.0 %	15,722	61.4 %	1,117	55.8 %
	Male	10,787	39.0 %	9,901	38.6 %	886	44.2 %
Birth season							
	Winter	6,434	23.3 %	5,959	23.3 %	475	23.7 %
	Spring	7,186	26.0 %	6,667	26.0 %	519	25.9 %
	Summer	7,172	26.0 %	6,658	26.0 %	514	25.7 %
	Autumn	6,834	24.7 %	6,339	24.7 %	495	24.7 %
Father's birth country							
	Finland	26,274	95.1 %	24,394	95.2 %	1,880	93.9 %
	Not in Finland	640	2.3 %	586	2.3 %	54	2.7 %
	Missing	712	2.6 %	643	2.5 %	69	3.4 %
Mother's birth country							
	Finland	26,915	97.4 %	24,972	97.5 %	1,943	97.0 %
	Not in Finland	478	1.7 %	444	1.7 %	34	1.7 %
	Missing	233	0.8 %	207	0.8 %	26	1.3 %
Father's education level							
	Low	7,958	28.8 %	7,395	28.9 %	563	28.1 %
	Middle	15,623	56.6 %	14,496	56.6 %	1,127	56.3 %
	High	3,297	11.9 %	3,062	12.0 %	235	11.7 %
	Missing	748	2.7 %	670	2.6 %	78	3.9 %
Mother's education level							
	Low	7,789	28.2 %	7,213	28.2 %	576	28.8 %
	Middle	17,010	61.6 %	15,779	61.6 %	1,231	61.5 %
	High	2,594	9.4 %	2,424	9.5 %	170	8.5 %
	Missing	233	0.8 %	207	0.8 %	26	1.3 %
Father's SES (occupation based)							
	Low	3,597	13.0 %	3,337	12.1 %	260	13.0 %
	Middle	4,195	15.2 %	3,897	14.1 %	298	14.9 %
	High	2,356	8.5 %	2,215	8.0 %	141	7.0 %
	Self-employed	15,130	54.8 %	14,026	50.8 %	1,104	55.1 %
	Missing	2,348	8.5 %	2,148	7.8 %	200	10.0 %
Mother's SES (occupation based)							
	High	2,891	10.5 %	2,716	10.6 %	175	8.7 %
	Intermediate	10,206	36.9 %	9,481	37.0 %	725	36.2 %
	Self-employed	1,290	4.7 %	1,211	4.7 %	79	3.9 %
	Low	11,300	40.9 %	10,429	40.7 %	871	43.5 %
	Missing	1,939	7.0 %	1,786	7.0 %	153	7.6 %
Urbanicity of the birthplace							
	Urban	17,391	63.0 %	16,082	62.8 %	1,309	65.5 %
	Semi-rural	3,900	14.1 %	3,602	14.1 %	298	14.9 %
	Rural	6,298	22.8 %	5,905	23.1 %	393	19.7 %
	Missing	37	0.1 %	-	-	<5	-
Low birth weight							
	No	26,953	97.6 %	24,999	97.6 %	1,954	97.6 %
	Yes	307	1.1 %	282	1.1 %	25	1.2 %
	Missing	366	1.3 %	342	1.3 %	24	1.2 %
Gestational age at birth (weeks)							
	Extremely preterm (-27)	58	0.2 %	49	0.2 %	9	0.4 %
	Very preterm (28-31)	154	0.6 %	136	0.5 %	18	0.9 %
	Moderately preterm (32-33)	174	0.6 %	165	0.6 %	9	0.4 %
	Late preterm (34-36)	1,111	4.0 %	1,031	4.0 %	80	4.0 %
	Early term (37-38)	4,860	17.6 %	4,510	17.6 %	350	17.5 %
	Term (39-41)	19,678	71.2 %	18,252	71.2 %	1,426	71.2 %
	Post term (42-44)	1,286	4.7 %	1,192	4.7 %	94	4.7 %
	Missing	305	1.1 %	288	1.1 %	17	0.8 %
Maternal age (years)							
	<20	1,376	5.0 %	1,238	4.8 %	138	6.9 %
	20-24	6,316	22.9 %	5,867	22.9 %	449	22.4 %
	25-29	9,472	34.3 %	8,825	34.4 %	647	32.3 %
	30-34	6,729	24.4 %	6,227	24.3 %	502	25.1 %
	35-39	2,984	10.8 %	2,766	10.8 %	218	10.9 %
	40-	749	2.7 %	700	2.7 %	49	2.4 %
Paternal age (years)							
	<20	372	1.3 %	341	1.3 %	31	1.5 %
	20-24	3,732	13.5 %	3,446	13.4 %	286	14.3 %
	25-29	8,362	30.3 %	7,778	30.4 %	584	29.2 %
	30-34	7,908	28.6 %	7,331	28.6 %	577	28.8 %
	35-39	4,147	15.0 %	3,856	15.0 %	291	14.5 %
	40-	2,394	8.7 %	2,229	8.7 %	165	8.2 %
	Missing	711	2.6 %	642	2.5 %	69	3.4 %

Note: Missing data reported only for variables with missing data.

Table S3 (continued). Sample characteristics.

Clinical and service use related prognostic factors	Total		Diagnosis of psychotic disorder in adulthood			
			No		Yes	
	N	%	N	%	N	%
Age at first adolescent psychiatry contact (years)						
13	6,749	24.4 %	6,270	24.5 %	479	23.9 %
14	5,264	19.1 %	4,930	19.2 %	334	16.7 %
15	5,425	19.6 %	5,033	19.6 %	392	19.6 %
16	5,237	19.0 %	4,870	19.0 %	367	18.3 %
17	4,951	17.9 %	4,520	17.6 %	431	21.5 %
Child psychiatry contact						
No	22,306	80.7 %	20,729	80.9 %	1,577	78.7 %
Yes	5,320	19.3 %	4,894	19.1 %	426	21.3 %
Psychiatric inpatient admission in adolescence						
No	20,099	72.8 %	18,933	73.9 %	1,166	58.2 %
Yes	7,527	27.2 %	6,690	26.1 %	837	41.8 %
Family history of psychosis						
No	26,887	97.3 %	24,976	97.5 %	1,911	95.4 %
Yes	739	2.7 %	647	2.5 %	92	4.6 %
Family history of serious mental illness						
No	22,248	80.5 %	20,750	81.0 %	1,498	74.8 %
Yes	5,378	19.5 %	4,873	19.0 %	505	25.2 %

Note: Missing data reported only for variables with missing data.

Table S4. Proportion of psychosis cases that were preceded by different adolescent mental disorders (sensitivity).

Mental disorder diagnosis in adolescence	Sensitivity for capturing psychosis % (n) [a]
Any adolescent mental disorder	100% (279)
Substance use disorders	10.8 % (30)
Mood disorders	49.1 % (137)
Anxiety disorders	35.8 % (100)
Eating disorders	12.5 % (35)
Personality disorders	6.1 % (17)
Autism spectrum disorders	2.2 % (6)
Childhood and adolescence onset disorders	40.1 % (112)

Note:
a: Total N of psychosis cases in adulthood occurring in adolescent psychiatry patients = 279

The sample restricted to individuals born in year 1987, excluding individuals who died or emigrated before the end of the follow-up, or who were diagnosed with psychosis before age 18.

Table S5. Total number of different sociodemographic prognostic factors, cumulative risk of psychosis and sensitivity for capturing psychosis (i.e. proportion of psychosis cases that were preceded by different total N of prognostic factors)

			Risk of psychosis				Sensitivity for capturing psychosis % (n) [b]
**Total N of prognostic factors**	**N**	**%**	**Cumulative risk of psychosis [a] (95%CI)**		**HR (95%CI)**		
Any							100% (279)
0	2,137	7.7 %	6.1%	(5.1%-7.3%)	Ref.		6.8 % (19)
1	10,491	38.0 %	7.6%	(6.9%-8.3%)	1.18	(0.97-1.44)	31.2 % (87)
2	10,626	38.5 %	9.0%	(8.1%-10%)	**1.40**	**(1.15-1.7)**	41.2 % (115)
3 or more	4,372	15.8 %	10.9%	(9.8%-12.1%)	**1.76**	**(1.44-2.16)**	20.8 % (58)

Note :
HR: Hazard ratio, CI: confidence interval

a: Cumulative risk by the end of the follow-up
b: Total N of psychosis cases occurring in adulthood in adolescent psychiatry patients = 279

Prognostic factors included: male sex; mother's low SES; born in an urban or semi-rural area; mother aged <20 years; low birth weight (for gestational age); born extremely and very prematurely.

For the analyses on sensitivity for capturing psychosis, the sample is restricted to individuals born in year 1987, excluding individuals who died or emigrated before the end of the follow-up, or who were diagnosed with psychosis before age 18. The proportional hazards assumption was tested using Schoenfeld residuals.

Cochran–Armitage test for trend p<.001
Departure from linear trend p=.40

Bold font indicates statistically significant association.

Table S6. Proportion of psychosis cases that were preceded by different sociodemographic prognostic factors (sensitivity).

Prognostic factor		Sensitivity for capturing psychosis % (n) [a]
Sex		
	Female	55.6% (155)
	Male	44.4% (124)
Birth season		
	Winter	24% (67)
	Spring	27.6% (77)
	Summer	24.7% (69)
	Autumn	23.7% (66)
Father's birth country		
	Finland	93.5% (261)
	Not in Finland	2.2% (6)
Mother's birth country		
	Finland	97.1% (271)
	Not in Finland	1.8% (5)
Father's education level		
	Low	32.3% (90)
	Middle	52% (145)
	High	11.1% (31)
Mother's education level		
	Low	33.3% (93)
	Middle	55.9% (156)
	High	9.7% (27)
Father's SES (occupation based)		
	Low	9.7% (27)
	Middle	14.3% (40)
	High	8.2% (23)
	Self-employed	57% (159)
Mother's SES (occupation based)		
	Low	8.2% (23)
	Middle	36.2% (101)
	High	4.7% (13)
	Self-employed	41.6% (116)
Urbanicity of the birth place		
	Urban	66.7% (186)
	Semi-rural	12.2% (34)
	Rural	21.1% (59)
Low birth weight		
	No	97.8% (273)
	Yes	1.8% (5)
Gestational age at birth (weeks)		
	Extremely preterm (<28)	- (<5)
	Very preterm (28-31)	2.2% (6)
	Moderately preterm (32-33)	- (<5)
	Late preterm (34-36)	5% (14)
	Early term (37-38)	18.6% (52)
	Term (39-41)	67.7% (189)
	Post term (42-44)	5.7% (16)
Maternal age		
	<20	11.1% (31)
	20-24	24.4% (68)
	25-29	25.4% (71)
	30-34	29.7% (83)
	35-39	7.5% (21)
	40-	1.8% (5)
Paternal age		
	<20	2.9% (8)
	20-24	16.5% (46)
	25-29	27.6% (77)
	30-34	26.2% (73)
	35-39	16.1% (45)
	40-	6.5% (18)

Note:
a: Total N of psychosis cases in adulthood occurring in adolescent psychiatry patients= 279

The sample restricted to individuals born in year 1987, excluding individuals who died or emigrated before the end of the follow-up, or who were diagnosed with psychosis before age 18.

Table S7. Total number of different clinical and service use related prognostic factors, cumulative risk of psychosis and sensitivity for capturing psychosis (i.e. proportion of psychosis cases that were preceded by different total N of prognostic factors)

			Risk of psychosis				Sensitivity for capturing psychosis % (n) [c]
**Total N of prognostic factors**	**N**	**%**	**Cumulative risk of psychosis [a] (95%CI)**		**HR (95%CI) [b]**		
Any							100% (279)
0	10,187	36.9 %	5.7%	(5.1%-6.3%)	Ref.		25.4 % (71)
1	12,141	43.9 %	8.8%	(8.0%-9.7%)	**1.58**	**(1.41-1.76)**	44.1 % (123)
2	4,237	15.3 %	12.9%	(11.6%-14.3%)	**2.43**	**(2.14-2.76)**	22.2 % (62)
3 or more	1,061	3.8 %	15.2%	(12.9%-17.8%)	**3.03**	**(2.52-3.65)**	8.2 % (23)

Note:
HR: Hazard ratio, CI: confidence interval

a: Cumulative risk by the end of the follow-up
b: Statistically significant non-proportionality of hazards was observed. The estimates should be interpreted as the average effect over the follow-up period. For cumulative risk curves, please see Supplementary Figure S35.
c: Total N of psychosis cases occurring in adulthood in adolescent psychiatry patients = 279

Prognostic factors included: attended adolescent psychiatry services first time when aged 17 years; child psychiatry visit; psychiatric inpatient admission in adolescence; family history of serious mental illness; family history of psychosis.

For the analyses on sensitivity for capturing psychosis, the sample is restricted to individuals born in year 1987, excluding individuals who died or emigrated before the end of the follow-up, or who were diagnosed with psychosis before age 18.

Cochran–Armitage test for trend p<.001
Departure from linear trend p=.25

Bold font indicates statistically significant association.

Table S8. Proportion of psychosis cases that were preceded by different clinical and service use related prognostic factors (sensitivity).

Prognostic factor		Sensitivity for capturing psychosis % (n) [a]
Age at first adolescent psychiatry contact (years)		
	13	24.7% (69)
	14	17.2% (48)
	15	22.2% (62)
	16	16.8% (47)
	17	19% (53)
Child psychiatry contact		
	No	82.1% (229)
	Yes	17.9% (50)
Psychiatric inpatient admission in adolescence		
	No	53.4% (149)
	Yes	46.6% (130)
Family history of psychosis		
	No	95% (265)
	Yes	5% (14)
Family history of serious mental illness		
	No	73.8% (206)
	Yes	26.2% (73)

Note:
a: Total N of psychosis cases occurring in adulthood in adolescent psychiatry patients= 279

The sample restricted to individuals born in year 1987, excluding individuals who died or emigrated before the end of the follow-up, or who were diagnosed with psychosis before age 18.

Table 9. Sensitivity analysis: Prognostic factors and cumulative risk of **psychosis in adulthood among FEMALE adolescent psychiatry patients** (Prognostic factor categories: Mental disorder diagnoses assigned in adolescence; Sociodemographic prognostic factors; Clinical and service use related prognostic factors).

Prognostic factors		N	%	% Risk of psychotic disorder [a]		HR (95% CI) [b]		HR (95% CI), adjusted [c]	
**Mental disorder diagnoses in adolescence**									
Substance use disorders									
	No	15,308	90.9 %	7.8 %	(7.1%-8.5%)	Ref.		Ref.	
	Yes	1,531	9.1 %	7.8 %	(6.4%-9.4%)	1.04	(0.85-1.27)	**1.24**	**(1.01-1.51)**
Mood disorders									
	No	9,021	53.6 %	5.9 %	(5.0%-6.9%)	Ref.		Ref.	
	Yes	7,818	46.4 %	9.9 %	(9.1%-10.8%)	**1.92**	**(1.71-2.17)**	**2.20**	**(1.94-2.48)**
Anxiety disorders									
	No	10,018	59.5 %	7.0 %	(6.4%-7.7%)	Ref.		Ref.	
	Yes	6,821	40.5 %	8.9 %	(7.7%-10.4%)	**1.23**	**(1.09-1.38)**	**1.54**	**(1.37-1.74)**
Obsessive-compulsive disorder									
	No	16,481	97.9 %	7.7 %	(7.1%-8.4%)	Ref.			
	Yes	358	2.1 %	10.8 %	(7.8%-14.7%)	**1.61**	**(1.15-2.24)**	-	-
Eating disorders									
	No	13,847	82.2 %	7.7 %	(7.0%-8.4%)	Ref.		Ref.	
	Yes	2,992	17.8 %	8.3 %	(7.0%-9.8%)	1.12	(0.97-1.30)	**1.44**	**(1.24-1.68)**
Personality disorders									
	No	16,388	97.3 %	7.6 %	(7.0%-8.3%)	Ref.		Ref.	
	Yes	451	2.7 %	13.6 %	(10.4%-17.7%)	**1.97**	**(1.51-2.59)**	**1.93**	**(1.47-2.53)**
Autism spectrum disorders									
	No	16,665	99.0 %	7.7 %	(7.1%-8.4%)	Ref.		Ref.	
	Yes	174	1.0 %	14.8 %	(8.6%-24.6%)	**1.95**	**(1.26-3.00)**	**2.51**	**(1.63-3.87)**
Childhood and adolescence onset disorders									
	No	11,914	70.8 %	7.1 %	(6.5%-7.7%)	Ref.		Ref.	
	Yes	4,925	29.2 %	9.4 %	(7.9%-11.3%)	**1.27**	**(1.12-1.44)**	**1.42**	**(1.25-1.61)**
ADHD									
	No	16,550	98.3 %	7.8 %	(7.1%-8.5%)	Ref.			
	Yes	289	1.7 %	8.7 %	(4.9%-15.4%)	0.97	(0.61-1.54)	-	-
Conduct disorders									
	No	14,927	88.6 %	7.7 %	(7.1%-8.5%)	Ref.			
	Yes	1,912	11.4 %	8.2 %	(7.0%-9.6%)	**1.20**	**(1.01-1.42)**	-	-
Tic disorders									
	No	16,768	99.6 %	7.8 %	(7.1%-8.4%)	Ref.			
	Yes	71	0.4 %	12.4 %	(4.0%-35%)	1.05	(0.43-2.52)	-	-
Other Neurodevelopmental disorders									
	No	16,341	97.0 %	7.7 %	(7.1%-8.4%)	Ref.			
	Yes	498	3.0 %	9.7 %	(7.1%-13.2%)	1.26	(0.92-1.71)	-	-
**Sociodemographic prognostic factors**									
Birth season									
	Winter	3,973	23.6 %	7.8 %	(6.7%-9.1%)	Ref.		Ref.	
	Spring	4,348	25.8 %	7.3 %	(6.4%-8.3%)	0.97	(0.82-1.15)	0.96	(0.80-1.15)
	Summer	4,353	25.9 %	7.9 %	(7.0%-9.0%)	1.02	(0.87-1.21)	1.04	(0.87-1.25)
	Autumn	4,165	24.7 %	7.8 %	(6.7%-8.9%)	1.01	(0.85-1.20)	1.01	(0.84-1.21)
Father's birth country									
	Finland	16,029	97.7 %	7.7 %	(7.1%-8.4%)	Ref.		Ref.	
	Not in Finland	380	2.3 %	7.7 %	(5.1%-11.6%)	1.02	(0.69-1.52)	0.82	(0.45-1.50)
Mother's birth country									
	Finland	16,382	98.2 %	7.8 %	(7.2%-8.5%)	Ref.		Ref.	
	Not in Finland	308	1.8 %	4.6 %	(2.7%-7.6%)	0.70	(0.42-1.19)	0.67	(0.33-1.35)
Father's education level									
	Low	4,737	28.9 %	6.9 %	(6.1%-7.8%)	Ref.		Ref.	
	Middle	9,553	58.3 %	8.1 %	(7.2%-9.2%)	1.12	(0.98-1.29)	1.18	(1.01-1.38)
	High	2,099	12.8 %	7.9 %	(6.3%-9.8%)	1.06	(0.87-1.30)	1.23	(0.92-1.64)
Mother's education level									
	Low	4,557	27.3 %	7.4 %	(6.5%-8.5%)	Ref.		Ref.	
	Middle	10,453	62.6 %	8.0 %	(7.2%-9.0%)	1.04	(0.91-1.20)	1.09	(0.93-1.29)
	High	1,680	10.1 %	7.1 %	(5.4%-9.2%)	0.91	(0.72-1.14)	0.98	(0.72-1.34)
Father's SES (occupation based)									
	High	2,247	14.5 %	8.0 %	(6.5%-9.9%)	Ref.		Ref.	
	Intermediate	2,648	17.1 %	7.5 %	(6.4%-8.7%)	1.00	(0.80-1.24)	1.03	(0.78-1.35)
	Self-employed	1,472	9.5 %	7.1 %	(5.6%-8.9%)	0.90	(0.70-1.18)	0.98	(0.70-1.37)
	Low	9,083	58.8 %	7.9 %	(6.9%-9.0%)	1.00	(0.84-1.20)	1.06	(0.82-1.38)
Mother's SES (occupation based)									
	High	1,833	11.7 %	7.3 %	(5.5%-9.6%)	Ref.		Ref.	
	Intermediate	6,313	40.3 %	7.4 %	(6.7%-8.2%)	1.11	(0.90-1.38)	1.16	(0.87-1.53)
	Self-employed	811	5.2 %	7.0 %	(5.1%-9.4%)	1.07	(0.76-1.50)	1.19	(0.79-1.79)
	Low	6,727	42.9 %	8.5 %	(7.3%-9.8%)	1.22	(0.99-1.51)	1.29	(0.96-1.71)

Table 9. (continued)

	N	%	% Risk of psychotic disorder [a]		HR (95% CI) [b]		HR (95% CI), adjusted [c]	
Urbanicity of the birthplace								
Urban	10,454	62.2 %	7.6 %	(7.0%-8.2%)	Ref.		Ref.	
Semi-rural	2,455	14.6 %	9.4 %	(8.1%-11.0%)	**1.21**	**(1.03-1.42)**	**1.22**	**(1.03-1.44)**
Rural	3,911	23.3 %	7.2 %	(5.6%-9.3%)	**0.84**	**(0.72-0.97)**	**0.84**	**(0.71-0.99)**
Low birth weight								
No	16,441	99.0 %	7.8 %	(7.2%-8.5%)	Ref.		Ref.	
Yes	171	1.0 %	10.0 %	(6.3%-15.6%)	1.51	(0.93-2.43)	1.11	(0.61-2.01)
Gestational age at birth (weeks)								
Extremely preterm (-27)	37	0.2 %	20.4 %	(10.1%-38.7%)	**3.12**	**(1.48-6.56)**	**4.17**	**(1.97-8.82)**
Very preterm (28-31)	85	0.5 %	13.2 %	(7.1%-24.0%)	**1.88**	**(1.01-3.50)**	**1.64**	**(0.78-3.45)**
Moderately preterm (32-33)	107	0.6 %	2.8 %	(0.9%-8.4%)	0.44	(0.14-1.36)	0.51	(0.16-1.58)
Late preterm (34-36)	642	3.9 %	8.5 %	(6.2%-11.4%)	1.15	(0.86-1.54)	1.27	(0.93-1.73)
Early term (37-38)	2,847	17.1 %	8.3 %	(7.2%-9.6%)	1.12	(0.96-1.30)	1.15	(0.97-1.35)
Term (39-41)	12,161	73.1 %	7.7 %	(6.9%-8.5%)	Ref.		Ref.	
Post term (42-44)	765	4.6 %	7.2 %	(5.5%-9.5%)	1.04	(0.79-1.39)	1.11	(0.82-1.50)
Maternal age (years)								
<20	787	4.7 %	12.3 %	(7.3%-20.5%)	Ref.		Ref.	
20-24	3,769	22.4 %	6.7 %	(5.8%-7.6%)	**0.72**	**(0.55-0.95)**	**0.65**	**(0.46-0.93)**
25-29	5,830	34.6 %	7.3 %	(6.5%-8.1%)	**0.77**	**(0.59-0.99)**	0.75	(0.52-1.09)
30-34	4,199	24.9 %	8.4 %	(7.2%-9.7%)	0.81	(0.62-1.05)	0.79	(0.53-1.17)
35-39	1,792	10.6 %	8.0 %	(6.6%-9.7%)	0.82	(0.61-1.10)	0.81	(0.52-1.24)
40-	462	2.7 %	7.0 %	(4.8%-10.1%)	0.74	(0.48-1.15)	0.72	(0.42-1.25)
Paternal age (years)								
<20	210	1.3 %	7.5 %	(4.6%-12.2%)	Ref.		Ref.	
20-24	2,247	13.7 %	9.1 %	(6.5%-12.8%)	0.98	(0.58-1.67)	1.26	(0.65-2.47)
25-29	5,077	30.9 %	7.0 %	(6.2%-8.0%)	0.88	(0.53-1.48)	1.15	(0.58-2.28)
30-34	4,883	29.8 %	7.9 %	(6.9%-8.9%)	0.95	(0.57-1.60)	1.24	(0.62-2.49)
35-39	2,590	15.8 %	7.4 %	(6.3%-8.7%)	0.92	(0.54-1.56)	1.21	(0.59-2.46)
40-	1,403	8.5 %	8.1 %	(6.5%-10.0%)	0.95	(0.55-1.64)	1.33	(0.64-2.77)
**Clinical and service use related prognostic factors**								
Age at first adolescent psychiatry contact (years)								
13	3,017	17.9 %	8.3 %	(7.0%-9.9%)	Ref.		Ref.	
14	3,271	19.4 %	6.6 %	(5.8%-7.7%)	0.86	(0.71-1.04)	1.00	(0.81-1.22)
15	3,675	21.8 %	8.1 %	(7.0%-9.5%)	0.98	(0.81-1.17)	**1.22**	**(1.00-1.49)**
16	3,578	21.2 %	6.7 %	(5.8%-7.7%)	0.87	(0.72-1.05)	1.22	(1.00-1.50)
17	3,298	19.6 %	9.3 %	(7.1%-12.1%)	1.07	(0.89-1.28)	**1.60**	**(1.30-1.95)**
Child psychiatry contact								
No	15,024	89.2 %	7.4 %	(6.8%-8.1%)	Ref.		Ref.	
Yes	1,815	10.8 %	10.9 %	(8.7%-13.8%)	**1.47**	**(1.25-1.74)**	**1.55**	**(1.29-1.86)**
Psychiatric inpatient admission in adolescence								
No	12,059	71.6 %	6.1 %	(5.4%-7.0%)	Ref.		Ref.	
Yes	4,780	28.4 %	11.9 %	(10.8%-13.1%)	**2.23**	**(1.98-2.51)**	**2.34**	**(2.07-2.64)**
Family history of psychosis								
No	16,405	97.4 %	7.6 %	(7.0%-8.3%)	Ref.		Ref.	
Yes	434	2.6 %	13.8 %	(10.4%-18.3%)	**1.92**	**(1.45-2.53)**	**1.57**	**(1.16-2.13)**
Family history of serious mental illness								
No	13,753	81.7 %	7.3 %	(6.6%-8.1%)	Ref.		Ref.	
Yes	3,086	18.3 %	9.8 %	(8.6%-11.1%)	**1.40**	**(1.22-1.61)**	**1.21**	**(1.04-1.41)**

Note:

HR: hazard ratio, CI: confidence interval

a: Cumulative risk by the end of the follow-up
b: Univariable Cox regression models; only including one prognostic factor at a time
c: Multivariable Cox regression model; including all prognostic factors in the prognostic factor category (in the prognostic factor category 'Mental disorder diagnoses', the subcategory diagnoses are not included in the model).

Bold font indicates statistically significant association.

Table 10. Sensitivity analysis: Prognostic factors and cumulative risk of **psychosis in adulthood among MALE adolescent psychiatry patients** (Prognostic factor categories: Mental disorder diagnoses assigned in adolescence; Sociodemographic prognostic factors; Clinical and service use related prognostic factors).

Prognostic factors		N	%	% Risk of psychotic disorder [a]		HR (95% CI) [b]		HR (95% CI), adjusted [c]	
**Mental disorder diagnoses in adolescence**									
Substance use disorders									
	No	9,328	86.5 %	9.3 %	(8.6%-10.1%)	Ref.		Ref.	
	Yes	1,459	13.5 %	11.7 %	(10.0%-13.7%)	**1.32**	**(1.10-1.57)**	**1.76**	**(1.46-2.11)**
Mood disorders									
	No	7,877	73.0 %	8.2 %	(7.5%-9.0%)	Ref.		Ref.	
	Yes	2,910	27.0 %	13.4 %	(12.1%-14.9%)	**1.78**	**(1.55-2.03)**	**2.04**	**(1.77-2.34)**
Anxiety disorders									
	No	7,720	71.6 %	9.1 %	(8.4%-10.0%)	Ref.		Ref.	
	Yes	3,067	28.4 %	10.8 %	(9.5%-12.3%)	**1.24**	**(1.07-1.42)**	**1.52**	**(1.31-1.76)**
Obsessive-compulsive disorder									
	No	10,449	96.9 %	9.6 %	(8.9%-10.3%)	Ref.			
	Yes	338	3.1 %	11.0 %	(7.8%-15.6%)	1.23	(0.87-1.74)	-	-
Eating disorders									
	No	10,354	96.0 %	9.6 %	(8.9%-10.3%)	Ref.		Ref.	
	Yes	433	4.0 %	10.3 %	(7.3%-14.4%)	1.01	(0.72-1.41)	1.22	(0.87-1.71)
Personality disorders									
	No	10,450	96.9 %	9.6 %	(8.9%-10.3%)	Ref.		Ref.	
	Yes	337	3.1 %	10.4 %	(7.3%-14.8%)	1.11	(0.78-1.59)	1.16	(0.81-1.67)
Autism spectrum disorders									
	No	10,040	93.1 %	9.6 %	(8.9%-10.3%)	Ref.		Ref.	
	Yes	747	6.9 %	10.6 %	(7.4%-15.1%)	0.94	(0.72-1.23)	**1.34**	**(1.02-1.76)**
Childhood and adolescence onset disorders									
	No	5,417	50.2 %	9.2 %	(8.3%-10.1%)	Ref.		Ref.	
	Yes	5,370	49.8 %	10.1 %	(9.1%-11.2%)	1.06	(0.93-1.21)	**1.45**	**(1.26-1.67)**
ADHD									
	No	9,407	87.2 %	9.8 %	(9.1%-10.6%)	Ref.			
	Yes	1,380	12.8 %	8.3 %	(6.5%-10.6%)	**0.76**	**(0.61-0.95)**	-	-
Conduct disorders									
	No	8,306	77.0 %	8.8 %	(8.1%-9.6%)	Ref.			
	Yes	2,481	23.0 %	12.3 %	(10.9%-13.8%)	**1.41**	**(1.22-1.63)**	-	-
Tic disorders									
	No	10,541	97.7 %	9.7 %	(9.0%-10.4%)	Ref.			
	Yes	246	2.3 %	6.9 %	(3.4%-13.7%)	0.57	(0.32-1.01)	-	-
Other Neurodevelopmental disorders									
	No	10,516	97.5 %	9.6 %	(8.9%-10.3%)	Ref.			
	Yes	271	2.5 %	13.2 %	(9.6%-17.8%)	**1.59**	**(1.13-2.23)**	-	-
**Sociodemographic prognostic factors**									
Birth season									
	Winter	2,461	22.8 %	9.9 %	(8.6%-11.3%)	Ref.		Ref.	
	Spring	2,838	26.3 %	9.7 %	(8.4%-11.2%)	0.96	(0.79-1.15)	0.92	(0.75-1.12)
	Summer	2,819	26.1 %	8.9 %	(7.7%-10.2%)	0.91	(0.75-1.10)	0.80	(0.65-0.99)
	Autumn	2,669	24.7 %	10.2 %	(8.8%-11.8%)	0.98	(0.82-1.19)	1.00	(0.82-1.23)
Father's birth country									
	Finland	10,245	97.5 %	9.4 %	(8.8%-10.2%)	Ref.		Ref.	
	Not in Finland	260	2.5 %	12.1 %	(8.5%-17.1%)	**1.46**	**(1.01-2.12)**	1.38	(0.85-2.24)
Mother's birth country									
	Finland	10,533	98.4 %	9.5 %	(8.8%-10.2%)	Ref.		Ref.	
	Not in Finland	170	1.6 %	13.0 %	(8.4%-19.9%)	1.52	(0.98-2.37)	1.40	(0.77-2.56)
Father's education level									
	Low	3,221	30.7 %	10.1 %	(8.8%-11.5%)	Ref.		Ref.	
	Middle	6,070	57.9 %	9.1 %	(8.3%-10.0%)	0.93	(0.80-1.08)	0.97	(0.82-1.15)
	High	1,198	11.4 %	9.6 %	(7.8%-11.8%)	0.98	(0.78-1.23)	1.06	(0.76-1.47)
Mother's education level									
	Low	3,232	30.2 %	10.0 %	(8.8%-11.3%)	Ref.		Ref.	
	Middle	6,557	61.3 %	9.3 %	(8.5%-10.2%)	0.93	(0.81-1.08)	0.97	(0.82-1.16)
	High	914	8.5 %	9.8 %	(7.6%-12.7%)	0.90	(0.70-1.17)	1.16	(0.81-1.65)
Father's SES (occupation based)									
	High	1,350	13.7 %	9.6 %	(7.9%-11.6%)	Ref.		Ref.	
	Intermediate	1,547	15.7 %	9.0 %	(7.4%-10.8%)	0.95	(0.74-1.23)	0.99	(0.71-1.36)
	Self-employed	884	9.0 %	7.4 %	(5.5%-10.0%)	**0.72**	**(0.52-0.99)**	0.75	(0.50-1.13)
	Low	6,047	61.5 %	9.7 %	(8.8%-10.7%)	0.98	(0.80-1.20)	0.93	(0.69-1.26)
Mother's SES (occupation based)									
	High	1,058	10.6 %	7.6 %	(5.8%-9.9%)	Ref.		Ref.	
	Intermediate	3,893	38.9 %	9.5 %	(8.4%-10.7%)	1.24	(0.95-1.61)	1.38	(0.99-1.93)
	Self-employed	479	4.8 %	8.6 %	(5.4%-13.6%)	0.92	(0.59-1.42)	1.32	(0.80-2.18)
	Low	4,573	45.7 %	9.9 %	(8.9%-11.0%)	**1.30**	**(1.01-1.68)**	**1.46**	**(1.04-2.05)**

Table 10. (continued)

				% Risk of psychotic disorder [a]		HR (95% CI) [b]		HR (95% CI), adjusted [c]	
Urbanicity of the birthplace									
	Urban	6,937	64.4 %	10.3 %	(9.5%-11.3%)	Ref.		Ref.	
	Semi-rural	1,445	13.4 %	8.3 %	(6.6%-10.3%)	**0.80**	**(0.65-0.99)**	0.85	(0.67-1.06)
	Rural	2,387	22.2 %	8.3 %	(7.1%-9.7%)	**0.80**	**(0.67-0.94)**	**0.80**	**(0.66-0.97)**
Low birth weight									
	No	10,512	98.7 %	9.6 %	(9.0%-10.4%)	Ref.		Ref.	
	Yes	136	1.3 %	8.9 %	(3.9%-19.3%)	0.71	(0.35-1.42)	0.47	(0.17-1.25)
Gestational age at birth (weeks)									
	Extremely preterm (-27)	21	0.2 %	10.9 %	(2.8%-37.3%)	1.13	(0.28-4.54)	-	-
	Very preterm (28-31)	69	0.6 %	13.9 %	(7.0%-26.5%)	1.31	(0.65-2.64)	1.67	(0.83-3.36)
	Moderately preterm (32-33)	67	0.6 %	9.0 %	(4.1%-18.9%)	1.09	(0.49-2.43)	0.96	(0.36-2.59)
	Late preterm (34-36)	469	4.4 %	8.0 %	(5.5%-11.7%)	0.82	(0.58-1.16)	0.84	(0.57-1.24)
	Early term (37-38)	2,013	18.9 %	8.6 %	(7.2%-10.2%)	0.84	(0.70-1.01)	0.89	(0.73-1.08)
	Term (39-41)	7,517	70.4 %	10.0 %	(9.2%-10.9%)	Ref.		Ref.	
	Post term (42-44)	521	4.9 %	9.0 %	(6.7%-12.1%)	0.97	(0.71-1.32)	0.85	(0.59-1.23)
Maternal age (years)									
	<20	589	5.5 %	13.7 %	(10.8%-17.2%)	Ref.		Ref.	
	20-24	2,547	23.6 %	9.7 %	(8.5%-11.1%)	**0.70**	**(0.53-0.91)**	0.74	(0.52-1.06)
	25-29	3,642	33.8 %	9.0 %	(7.8%-10.3%)	**0.61**	**(0.47-0.79)**	**0.60**	**(0.41-0.89)**
	30-34	2,530	23.5 %	9.7 %	(8.4%-11.2%)	**0.70**	**(0.54-0.92)**	**0.63**	**(0.42-0.95)**
	35-39	1,192	11.1 %	9.6 %	(7.7%-11.9%)	**0.64**	**(0.47-0.88)**	**0.62**	**(0.39-0.98)**
	40-	287	2.7 %	8.1 %	(5.1%-12.8%)	**0.56**	**(0.34-0.92)**	0.58	(0.31-1.12)
Paternal age (years)									
	<20	162	1.5 %	12.5 %	(7.5%-20.5%)	Ref.		Ref.	
	20-24	1,485	14.1 %	9.9 %	(8.3%-11.9%)	0.88	(0.52-1.47)	1.17	(0.61-2.25)
	25-29	3,285	31.3 %	9.5 %	(8.3%-10.9%)	0.82	(0.49-1.36)	1.26	(0.65-2.44)
	30-34	3,025	28.8 %	9.7 %	(8.5%-11.1%)	0.83	(0.50-1.38)	1.32	(0.67-2.59)
	35-39	1,557	14.8 %	9.6 %	(7.9%-11.6%)	0.80	(0.47-1.35)	1.36	(0.68-2.72)
	40-	991	9.4 %	7.7 %	(6.1%-9.6%)	0.72	(0.42-1.25)	1.17	(0.57-2.44)
**Clinical and service use related prognostic factors**									
Age at first adolescent psychiatry contact (years)									
	13	3,732	34.6 %	8.8 %	(7.7%-10.1%)	Ref.		Ref.	
	14	1,993	18.5 %	8.1 %	(6.8%-9.7%)	0.94	(0.77-1.16)	0.93	(0.75-1.16)
	15	1,750	16.2 %	9.2 %	(7.8%-10.9%)	1.12	(0.91-1.37)	1.13	(0.90-1.40)
	16	1,659	15.4 %	10.6 %	(8.9%-12.7%)	**1.27**	**(1.04-1.55)**	**1.34**	**(1.08-1.67)**
	17	1,653	15.3 %	12.7 %	(11.0%-14.6%)	**1.64**	**(1.36-1.98)**	**1.79**	**(1.46-2.21)**
Child psychiatry contact									
	No	7,282	67.5 %	9.8 %	(9.0%-10.6%)	Ref.		Ref.	
	Yes	3,505	32.5 %	9.4 %	(8.1%-10.9%)	0.88	(0.76-1.02)	1.01	(0.86-1.20)
Psychiatric inpatient admission in adolescence									
	No	8,040	74.5 %	8.3 %	(7.6%-9.1%)	Ref.		Ref.	
	Yes	2,747	25.5 %	13.5 %	(12.0%-15.0%)	**1.72**	**(1.50-1.97)**	**1.78**	**(1.55-2.04)**
Family history of psychosis									
	No	10,482	97.2 %	9.5 %	(8.8%-10.2%)	Ref.		Ref.	
	Yes	305	2.8 %	15.4 %	(11.2%-21.0%)	**1.68**	**(1.23-2.31)**	1.35	(0.96-1.89)
Family history of serious mental illness									
	No	8,495	78.8 %	8.9 %	(8.2%-9.7%)	Ref.		Ref.	
	Yes	2,292	21.2 %	12.1 %	(10.6%-13.8%)	**1.41**	**(1.21-1.63)**	**1.29**	**(1.10-1.51)**

Note:

HR: hazard ratio, CI: confidence interval

a: Cumulative risk by the end of the follow-up
b: Univariable Cox regression models; only including one prognostic factor at a time
c: Multivariable Cox regression model; including all prognostic factors in the prognostic factor category (in the prognostic factor category 'Mental disorder diagnoses', the subcategory diagnoses are not included in the model).

Bold font indicates statistically significant association.

Table 11. Sensitivity analysis: Prognostic factors and cumulative risk of **schizophrenia-spectrum disorder** in adulthood among adolescent psychiatry patients (Prognostic factor categories: Mental disorder diagnoses assigned in adolescence; Sociodemographic prognostic factors; Clinical and service use related prognostic factors).

Prognostic factors		N	%	% Risk of psychotic disorder [a]		HR (95% CI) [b]		HR (95% CI), adjusted [c]	
**Mental disorder diagnoses in adolescence**									
Substance use disorders									
	No	25,206	89.2 %	6.8 %	(6.4%-7.3%)	Ref.		Ref.	
	Yes	3,050	10.8 %	8.8 %	(7.7%-10.0%)	**1.38**	**(1.20-1.58)**	**1.67**	**(1.46-1.93)**
Mood disorders									
	No	16,906	59.8 %	6.1 %	(5.5%-6.7%)	Ref.		Ref.	
	Yes	11,350	40.2 %	8.5 %	(7.9%-9.2%)	**1.52**	**(1.38-1.68)**	**1.73**	**(1.57-1.91)**
Anxiety disorders									
	No	18,172	64.3 %	6.7 %	(6.3%-7.2%)	Ref.		Ref.	
	Yes	10,084	35.7 %	7.7 %	(6.7%-8.7%)	**1.15**	**(1.04-1.27)**	**1.40**	**(1.26-1.54)**
Obsessive-compulsive disorder									
	No	27,546	97.5 %	7.0 %	(6.5%-7.5%)	Ref.			
	Yes	710	2.5 %	9.5 %	(7.4%-12.1%)	**1.59**	**(1.24-2.05)**	-	-
Eating disorders									
	No	24,752	87.6 %	7.1 %	(6.7%-7.7%)	Ref.		Ref.	
	Yes	3,504	12.4 %	6.4 %	(5.3%-7.7%)	0.91	(0.78-1.06)	**1.12**	**(0.96-1.30)**
Personality disorders									
	No	27,360	96.8 %	6.9 %	(6.5%-7.4%)	Ref.		Ref.	
	Yes	896	3.2 %	10.9 %	(8.8%-13.3%)	**1.78**	**(1.44-2.20)**	**1.74**	**(1.40-2.15)**
Autism spectrum disorders									
	No	27,315	96.7 %	7.0 %	(6.5%-7.5%)	Ref.		Ref.	
	Yes	941	3.3 %	8.8 %	(6.4%-12.2%)	1.22	(0.95-1.56)	**1.66**	**(1.29-2.13)**
Childhood and adolescence onset disorders									
	No	17,751	62.8 %	6.3 %	(5.9%-6.8%)	Ref.		Ref.	
	Yes	10,505	37.2 %	8.3 %	(7.4%-9.4%)	**1.27**	**(1.15-1.40)**	**1.49**	**(1.35-1.64)**
ADHD									
	No	26,565	94.0 %	7.0 %	(6.6%-7.5%)	Ref.			
	Yes	1,691	6.0 %	8.1 %	(6.1%-10.7%)	0.97	(0.79-1.20)	-	-
Conduct disorders									
	No	23,794	84.2 %	6.6 %	(6.1%-7.2%)	Ref.			
	Yes	4,462	15.8 %	9.4 %	(8.4%-10.4%)	**1.53**	**(1.37-1.72)**	-	-
Tic disorders									
	No	27,937	98.9 %	7.0 %	(6.6%-7.5%)	Ref.			
	Yes	319	1.1 %	7.7 %	(4.2%-13.8%)	0.89	(0.55-1.43)	-	-
Other Neurodevelopmental disorders									
	No	27,458	97.2 %	7.0 %	(6.5%-7.5%)	Ref.			
	Yes	798	2.8 %	9.0 %	(7.1%-11.3%)	**1.41**	**(1.10-1.80)**	-	-
**Sociodemographic prognostic factors**									
Sex									
	Female	17,291	61.2 %	6.1 %	(5.5%-6.7%)	Ref.		Ref.	
	Male	10,965	38.8 %	8.6 %	(8.0%-9.3%)	**1.48**	**(1.35-1.63)**	**1.42**	**(1.28-1.58)**
Birth season									
	Winter	6,588	23.3 %	7.0 %	(6.2%-7.9%)	Ref.		Ref.	
	Spring	7,354	26.0 %	6.8 %	(6.1%-7.5%)	0.99	(0.86-1.14)	0.95	(0.82-1.11)
	Summer	7,315	25.9 %	7.0 %	(6.3%-7.8%)	1.01	(0.88-1.15)	0.97	(0.83-1.12)
	Autumn	6,999	24.8 %	7.2 %	(6.4%-8.1%)	1.03	(0.90-1.18)	1.04	(0.90-1.21)
Father's birth country									
	Finland	26,883	97.6 %	6.9 %	(6.5%-7.4%)	Ref.		Ref.	
	Not in Finland	651	2.4 %	7.7 %	(5.7%-10.4%)	1.21	(0.90-1.63)	1.03	(0.67-1.57)
Mother's birth country									
	Finland	27,529	98.3 %	7.0 %	(6.6%-7.5%)	Ref.		Ref.	
	Not in Finland	488	1.7 %	6.1 %	(4.1%-8.9%)	0.98	(0.67-1.44)	1.09	(0.68-1.73)
Father's education level									
	Low	8,127	29.6 %	6.7 %	(6.1%-7.4%)	Ref.		Ref.	
	Middle	15,983	58.1 %	7.0 %	(6.4%-7.7%)	1.00	(0.90-1.12)	1.05	(0.93-1.19)
	High	3,388	12.3 %	7.1 %	(5.9%-8.4%)	1.01	(0.86-1.20)	1.22	(0.97-1.55)
Mother's education level									
	Low	7,930	28.3 %	7.2 %	(6.5%-7.9%)	Ref.		Ref.	
	Middle	17,421	62.2 %	7.0 %	(6.4%-7.7%)	0.95	(0.85-1.06)	1.02	(0.89-1.16)
	High	2,666	9.5 %	6.7 %	(5.4%-8.4%)	0.87	(0.72-1.04)	0.97	(0.75-1.25)
Father's SES (occupation based)									
	High	3,693	14.3 %	6.9 %	(5.8%-8.1%)	Ref.		Ref.	
	Intermediate	4,292	16.6 %	6.4 %	(5.6%-7.3%)	0.95	(0.79-1.15)	1.05	(0.83-1.33)
	Self-employed	2,412	9.3 %	6.4 %	(5.2%-7.9%)	0.87	(0.70-1.09)	0.99	(0.75-1.32)
	Low	15,473	59.8 %	7.2 %	(6.5%-7.9%)	1.03	(0.89-1.20)	1.11	(0.89-1.38)
Mother's SES (occupation based)									
	High	2,968	11.3 %	6.5 %	(5.2%-8.1%)	Ref.		Ref.	
	Intermediate	10,433	39.7 %	6.7 %	(6.2%-7.3%)	1.10	(0.92-1.32)	1.11	(0.88-1.40)
	Self-employed	1,318	5.0 %	6.6 %	(5.0%-8.7%)	1.01	(0.76-1.34)	1.19	(0.85-1.67)
	Low	11,560	44.0 %	7.5 %	(6.7%-8.4%)	1.19	(1.00-1.41)	1.18	(0.93-1.49)

Table 11. (continued)

			% Risk of psychotic disorder [a]		HR (95% CI) [b]		HR (95% CI), adjusted [c]	
Urbanicity of the birthplace								
Urban	17,769	63.0 %	7.3 %	(6.9%-7.8%)	Ref.		Ref.	
Semi-rural	3,984	14.1 %	7.3 %	(6.3%-8.3%)	0.99	(0.86-1.14)	1.04	(0.90-1.21)
Rural	6,464	22.9 %	6.1 %	(5.0%-7.5%)	**0.75**	**(0.66-0.85)**	**0.76**	**(0.66-0.87)**
Low birth weight								
No	27,563	98.9 %	7.0 %	(6.6%-7.5%)	Ref.		Ref.	
Yes	314	1.1 %	9.2 %	(6.1%-13.9%)	**1.35**	**(0.91-2.01)**	0.98	(0.59-1.63)
Gestational age at birth (weeks)								
Extremely preterm (-27)	61	0.2 %	19.7 %	(10.2%-36.1%)	**2.62**	**(1.36-5.05)**	**2.59**	**(1.23-5.44)**
Very preterm (28-31)	159	0.6 %	9.3 %	(5.6%-15.3%)	1.51	(0.89-2.56)	1.56	(0.88-2.76)
Moderately preterm (32-33)	179	0.6 %	6.1 %	(3.5%-10.8%)	1.10	(0.60-1.98)	0.99	(0.49-1.99)
Late preterm (34-36)	1,136	4.1 %	6.3 %	(4.7%-8.4%)	0.86	(0.66-1.12)	0.90	(0.68-1.20)
Early term (37-38)	4,972	17.8 %	7.1 %	(6.3%-8.0%)	1.04	(0.92-1.18)	1.06	(0.93-1.22)
Term (39-41)	20,115	72.0 %	7.1 %	(6.5%-7.7%)	Ref.		Ref.	
Post term (42-44)	1,317	4.7 %	7.0 %	(5.6%-8.6%)	1.11	(0.89-1.38)	1.10	(0.86-1.41)
Maternal age (years)								
<20	1,404	5.0 %	10.5 %	(7.4%-14.9%)	Ref.		Ref.	
20-24	6,451	22.8 %	6.8 %	(6.2%-7.6%)	**0.76**	**(0.61-0.94)**	**0.76**	**(0.58-1.00)**
25-29	9,693	34.3 %	6.4 %	(5.8%-7.0%)	**0.69**	**(0.56-0.84)**	**0.68**	**(0.51-0.91)**
30-34	6,883	24.4 %	7.4 %	(6.5%-8.3%)	**0.77**	**(0.62-0.94)**	**0.71**	**(0.52-0.97)**
35-39	3,059	10.8 %	7.0 %	(5.9%-8.3%)	**0.73**	**(0.58-0.93)**	**0.69**	**(0.49-0.98)**
40-	766	2.7 %	6.1 %	(4.4%-8.4%)	**0.65**	**(0.45-0.93)**	**0.63**	**(0.39-0.99)**
Paternal age (years)								
<20	380	1.4 %	6.6 %	(4.3%-10.3%)	Ref.		Ref.	
20-24	3,825	13.9 %	8.1 %	(6.4%-10.3%)	1.14	(0.73-1.76)	1.44	(0.83-2.48)
25-29	8,542	31.0 %	6.5 %	(5.9%-7.3%)	0.99	(0.65-1.52)	1.40	(0.80-2.44)
30-34	8,089	29.4 %	7.1 %	(6.3%-7.9%)	1.03	(0.67-1.57)	1.53	(0.87-2.69)
35-39	4,243	15.4 %	6.7 %	(5.9%-7.7%)	0.99	(0.64-1.53)	1.55	(0.87-2.76)
40-	2,456	8.9 %	6.5 %	(5.4%-7.7%)	0.99	(0.63-1.55)	1.58	(0.88-2.87)
**Clinical and service use related prognostic factors**								
Age at first adolescent psychiatry contact (years)								
13	6,877	24.3 %	7.5 %	(6.6%-8.5%)	Ref.		Ref.	
14	5,403	19.1 %	5.9 %	(5.2%-6.7%)	0.83	(0.71-0.97)	0.93	(0.79-1.10)
15	5,568	19.7 %	7.0 %	(6.1%-8.0%)	0.96	(0.83-1.11)	**1.15**	**(0.98-1.34)**
16	5,356	19.0 %	6.1 %	(5.4%-6.8%)	0.91	(0.78-1.05)	1.18	(1.00-1.39)
17	5,052	17.9 %	8.8 %	(7.2%-10.8%)	**1.20**	**(1.05-1.39)**	**1.65**	**(1.41-1.94)**
Child psychiatry contact								
No	22,849	80.9 %	6.7 %	(6.2%-7.2%)	Ref.		Ref.	
Yes	5,407	19.1 %	8.7 %	(7.6%-10.0%)	**1.26**	**(1.12-1.41)**	**1.39**	**(1.21-1.58)**
Psychiatric inpatient admission in adolescence								
No	20,220	71.6 %	5.7 %	(5.2%-6.3%)	Ref.		Ref.	
Yes	8,036	28.4 %	10.5 %	(9.6%-11.3%)	**2.02**	**(1.83-2.22)**	**2.11**	**(1.91-2.33)**
Family history of psychosis								
No	27,492	97.3 %	6.9 %	(6.4%-7.4%)	Ref.		Ref.	
Yes	764	2.7 %	13.1 %	(10.4%-16.2%)	**2.03**	**(1.63-2.52)**	**1.57**	**(1.24-1.99)**
Family history of serious mental illness								
No	22,728	80.4 %	6.5 %	(6%-7.1%)	Ref.		Ref.	
Yes	5,528	19.6 %	9.1 %	(8.3%-10.1%)	**1.49**	**(1.33-1.66)**	**1.29**	**(1.14-1.45)**

Note:

HR: hazard ratio, CI: confidence interval

a: Cumulative risk by the end of the follow-up
b: Univariable Cox regression models; only including one prognostic factor at a time
c: Multivariable Cox regression model; including all prognostic factors in the prognostic factor category (in the prognostic factor category 'Mental disorder diagnoses', the subcategory diagnoses are not included in the model).

Bold font indicates statistically significant association.

SUPPLEMENTARY FIGURES

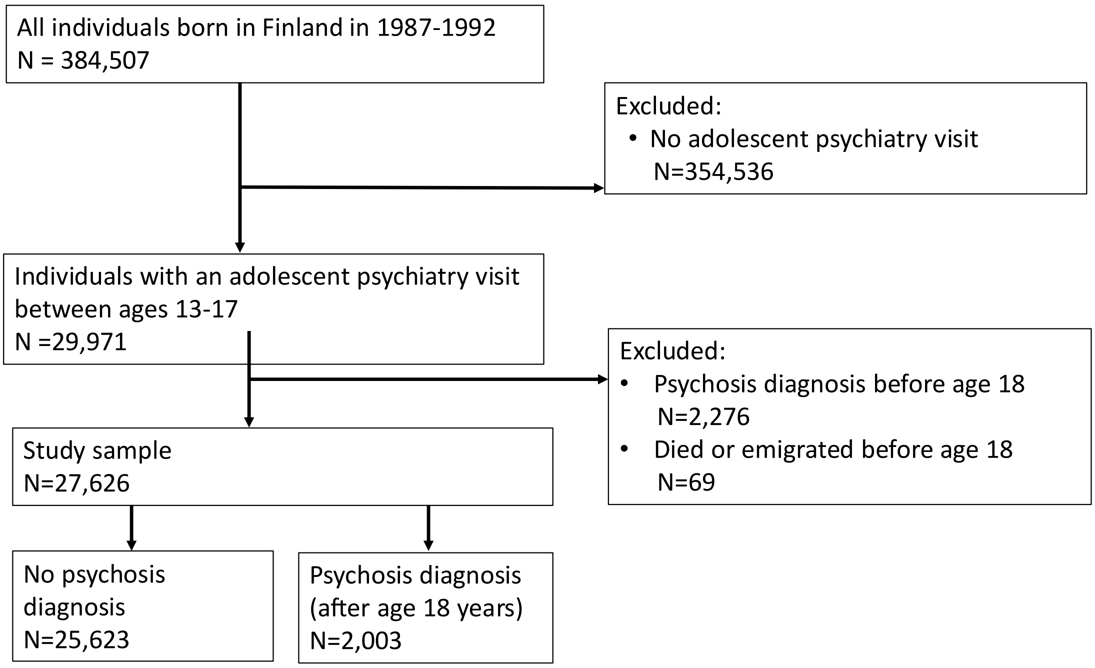

Figure S1. Study flowchart

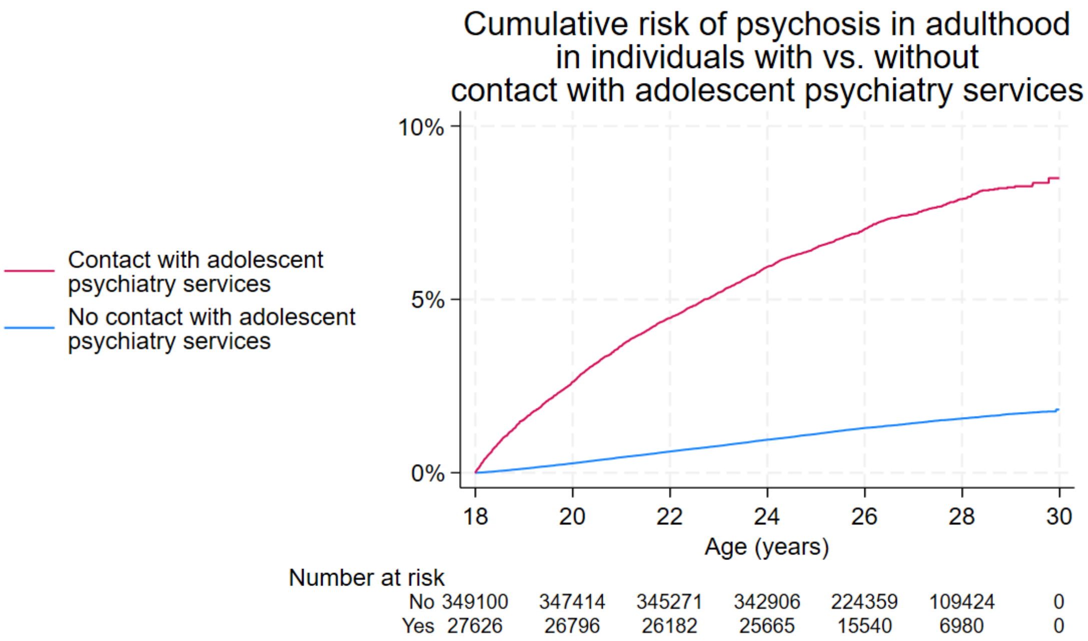

Figure S2. Cumulative risk of psychosis in adulthood in individuals with vs. without contact with adolescent psychiatry services; sample excluding individuals who died, emigrated or were diagnosed with psychotic disorder before age 18

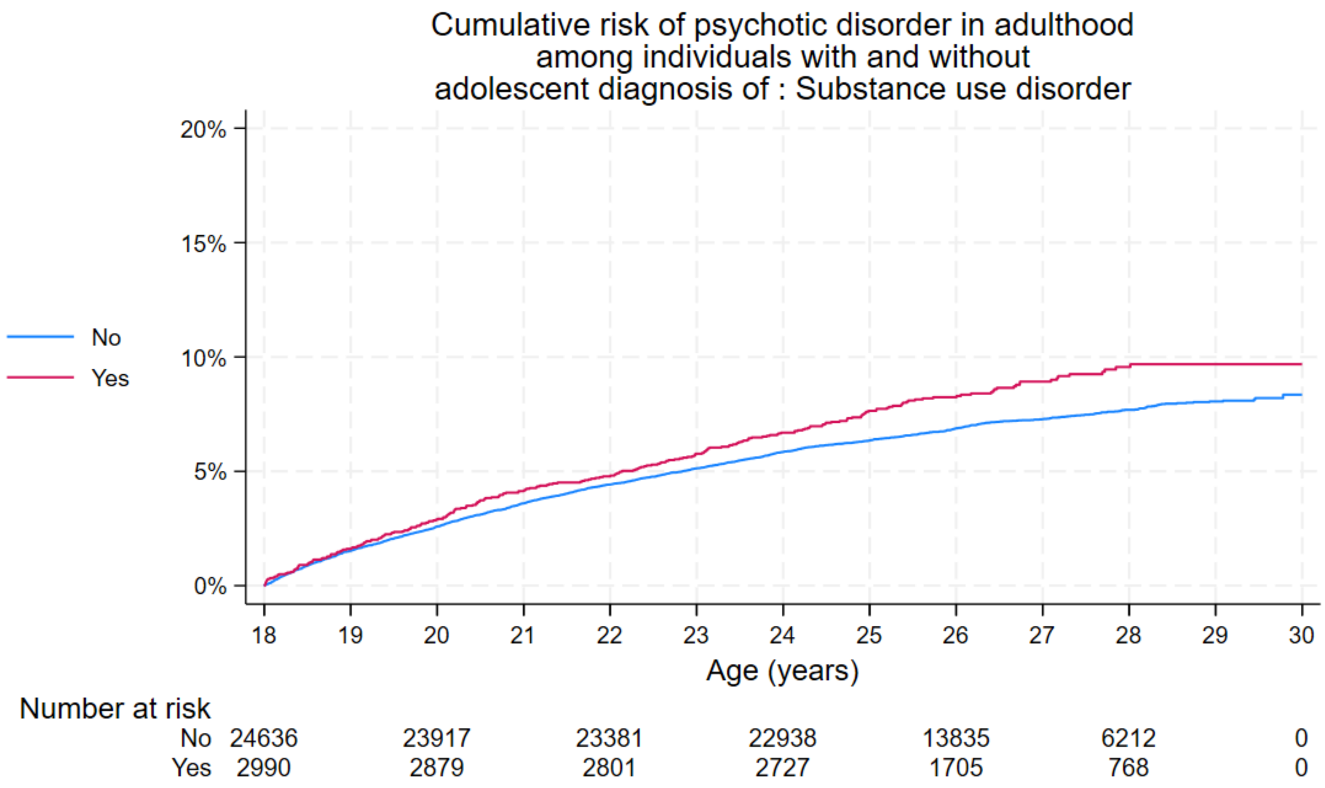

Figure S3. Cumulative risk of psychosis among individuals with and without an adolescent diagnosis of Substance use disorders.

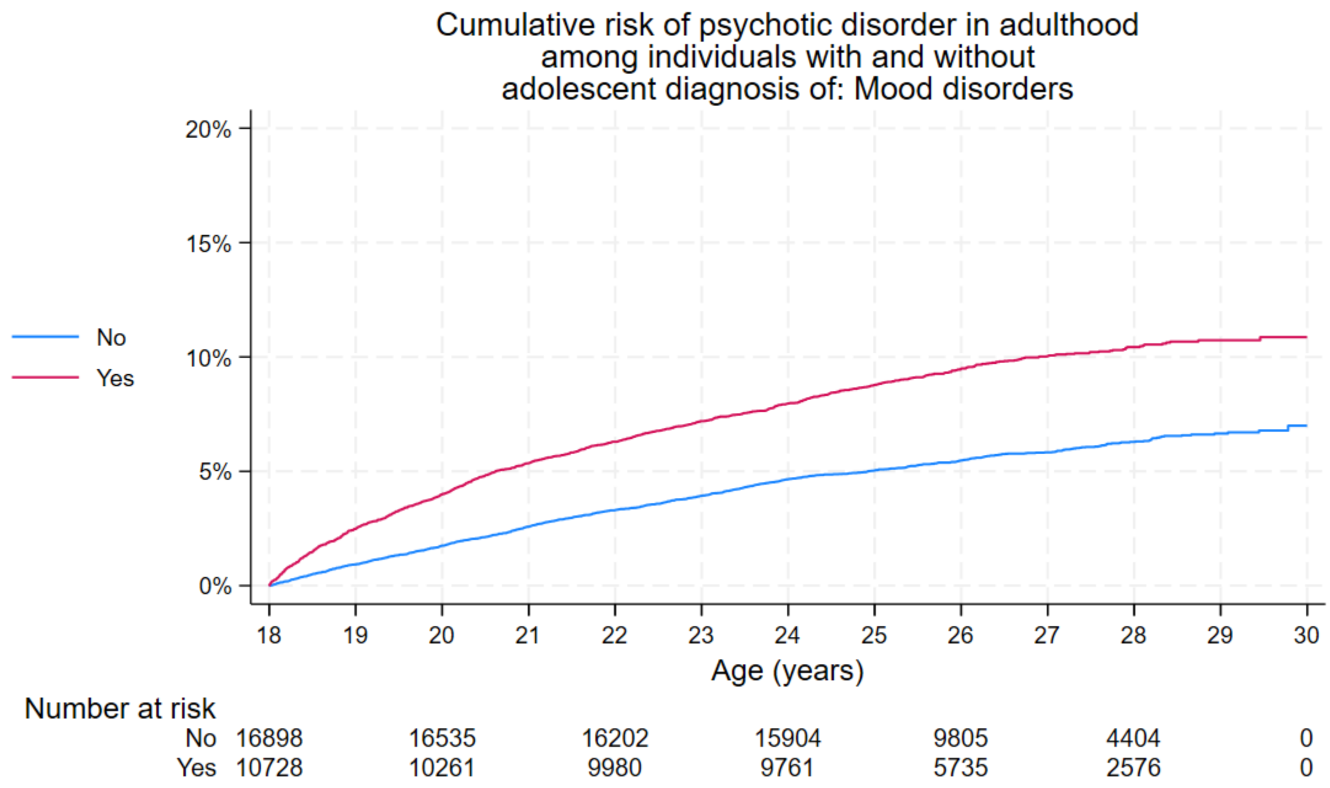

Figure S4. Cumulative risk of psychosis among individuals with and without an adolescent diagnosis of Mood disorders.

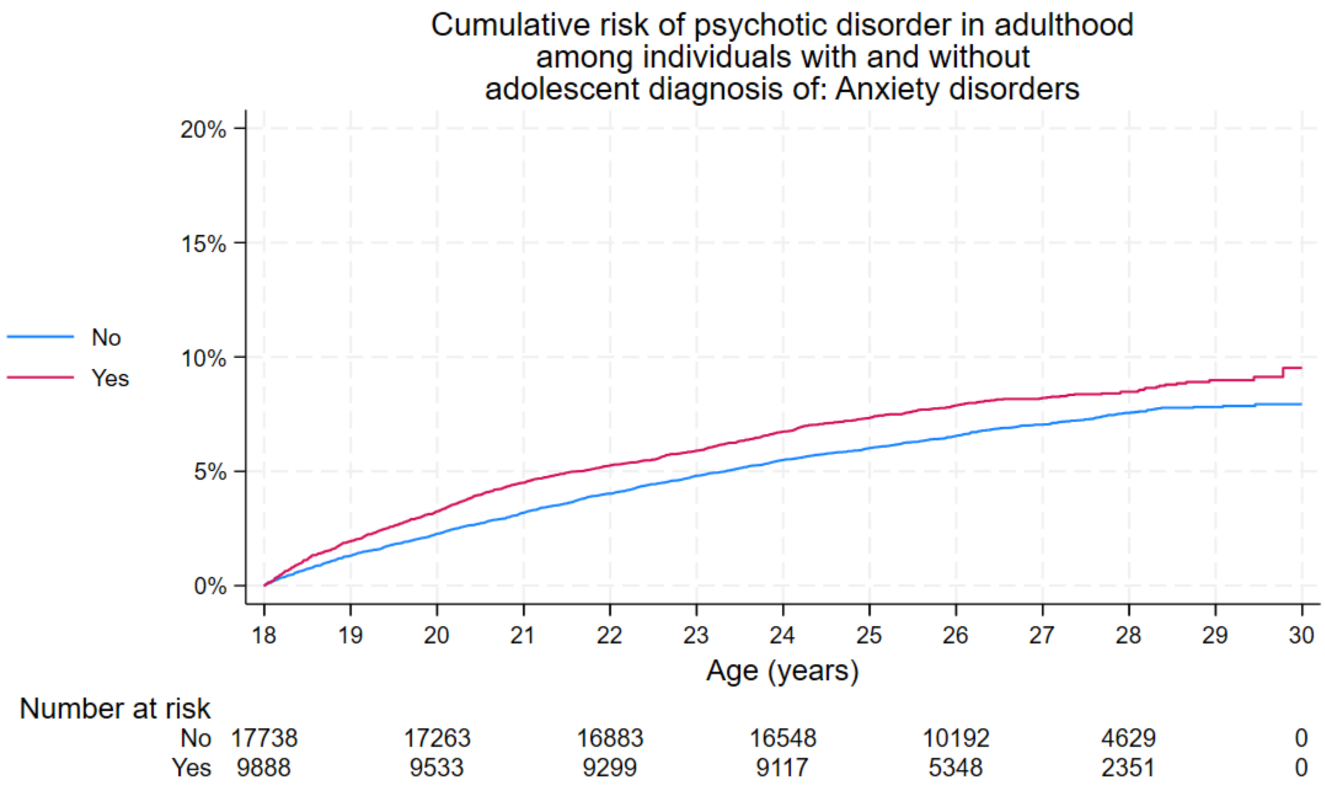

Figure S5. Cumulative risk of psychosis among individuals with and without an adolescent diagnosis of Anxiety disorders.

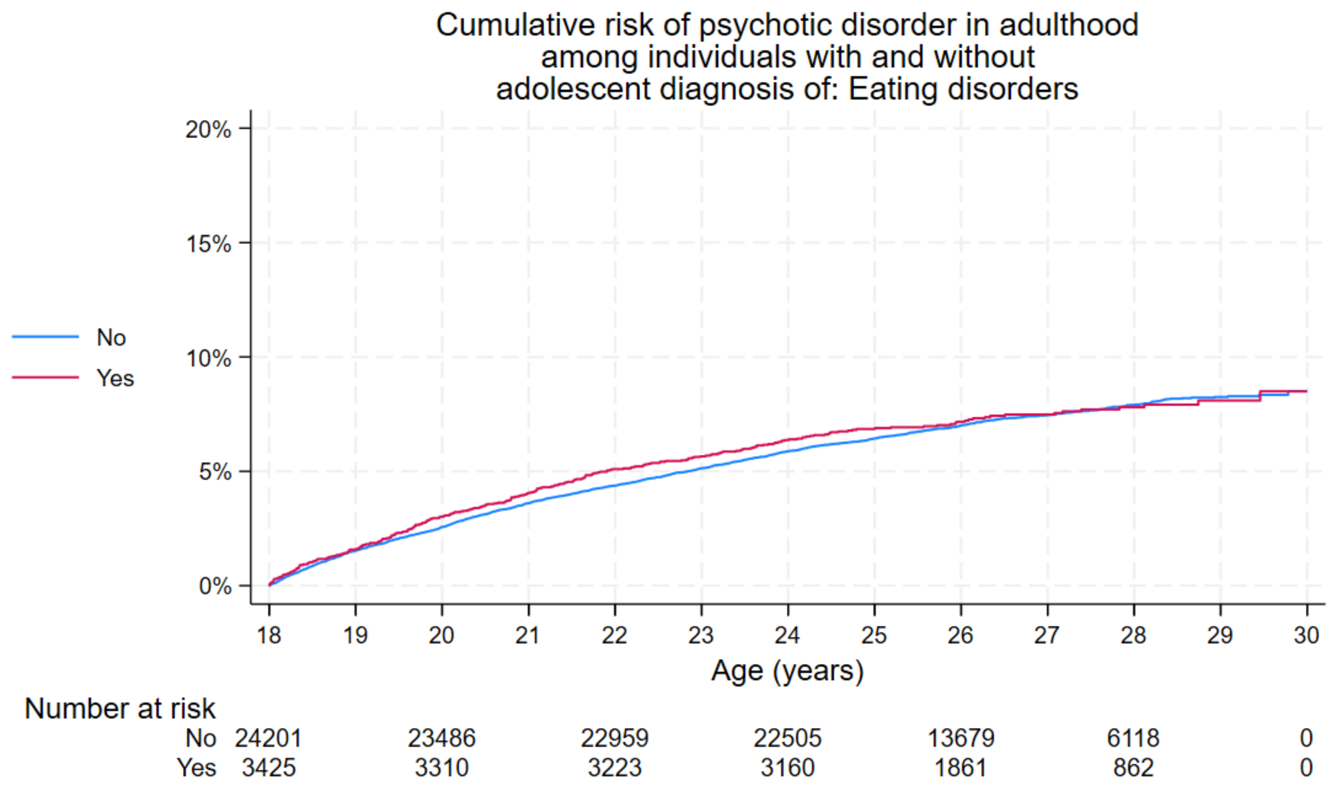

Figure S6. Cumulative risk of psychosis among individuals with and without an adolescent diagnosis of Eating disorders.

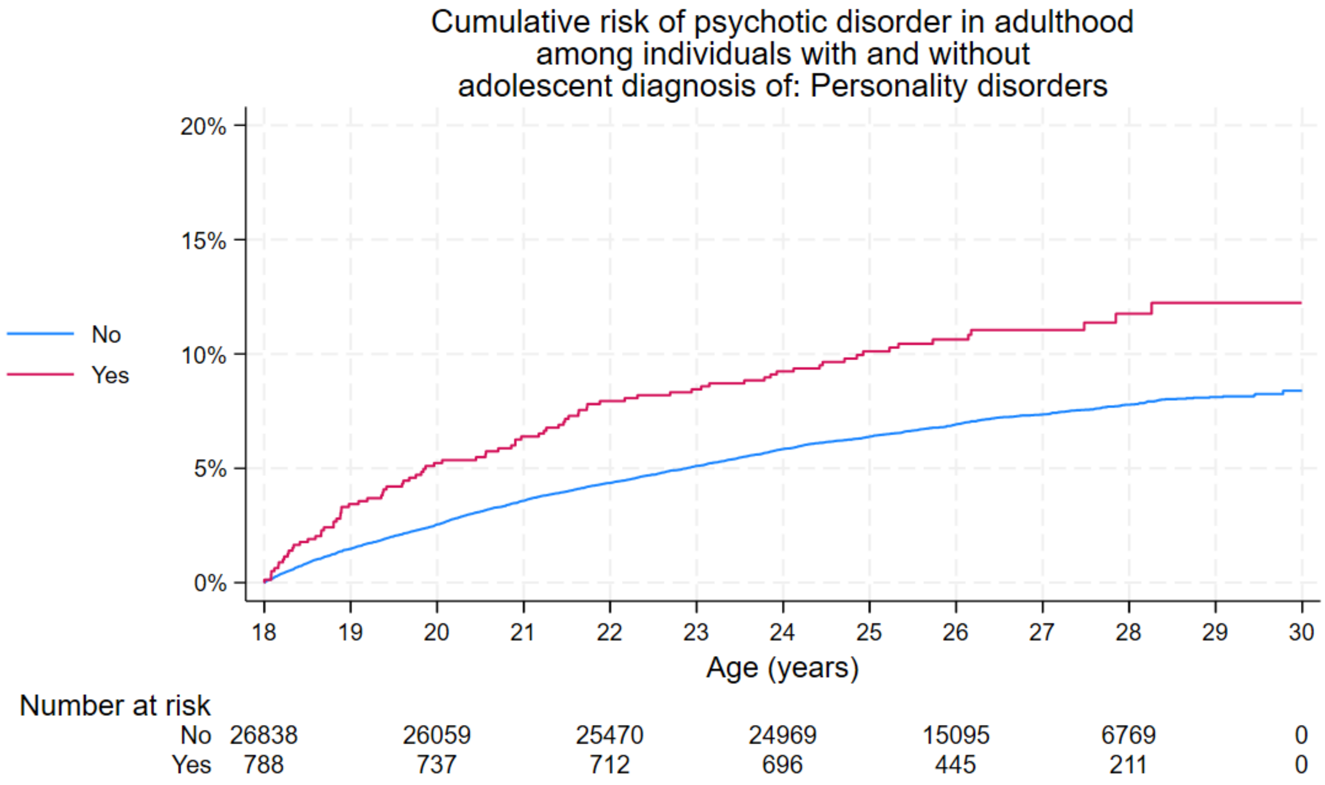

Figure S7. Cumulative risk of psychosis among individuals with and without an adolescent diagnosis of Personality disorders.

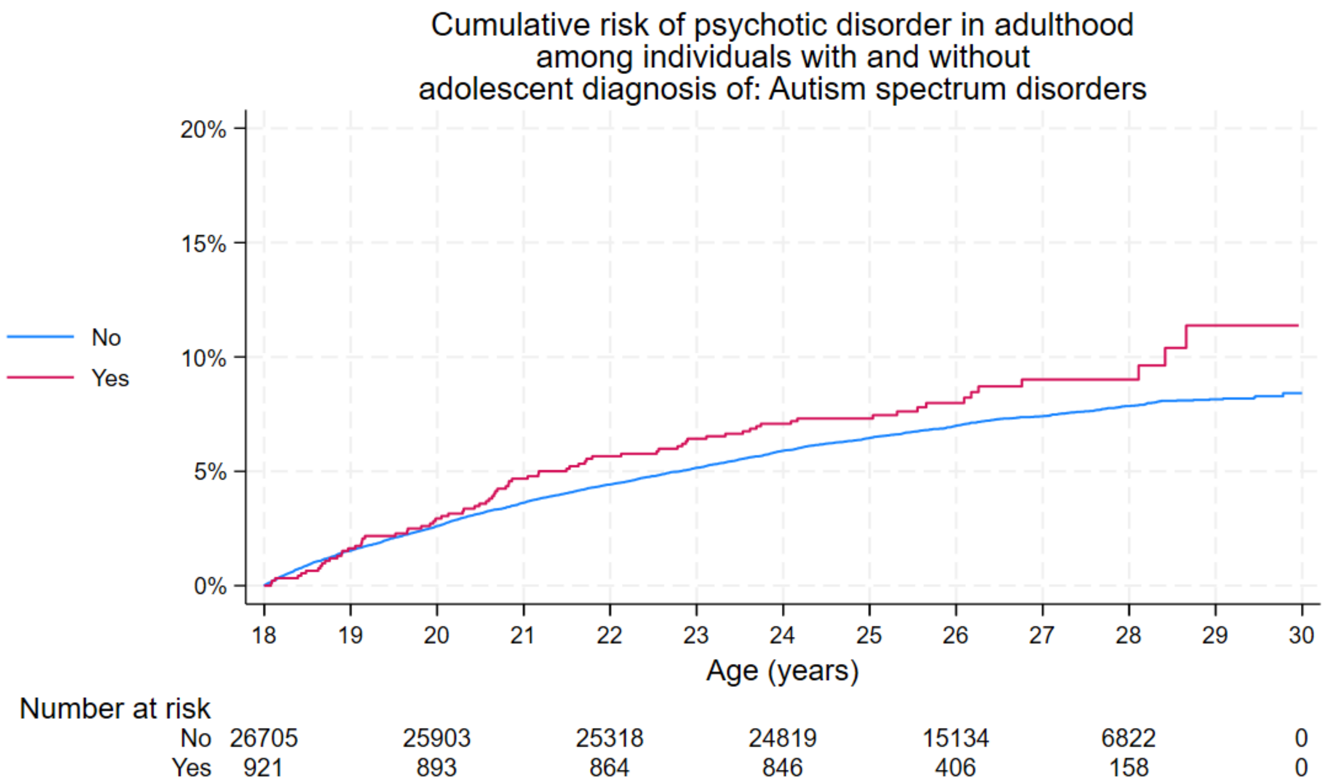

Figure S8. Cumulative risk of psychosis among individuals with and without an adolescent diagnosis of Autism spectrum disorders.

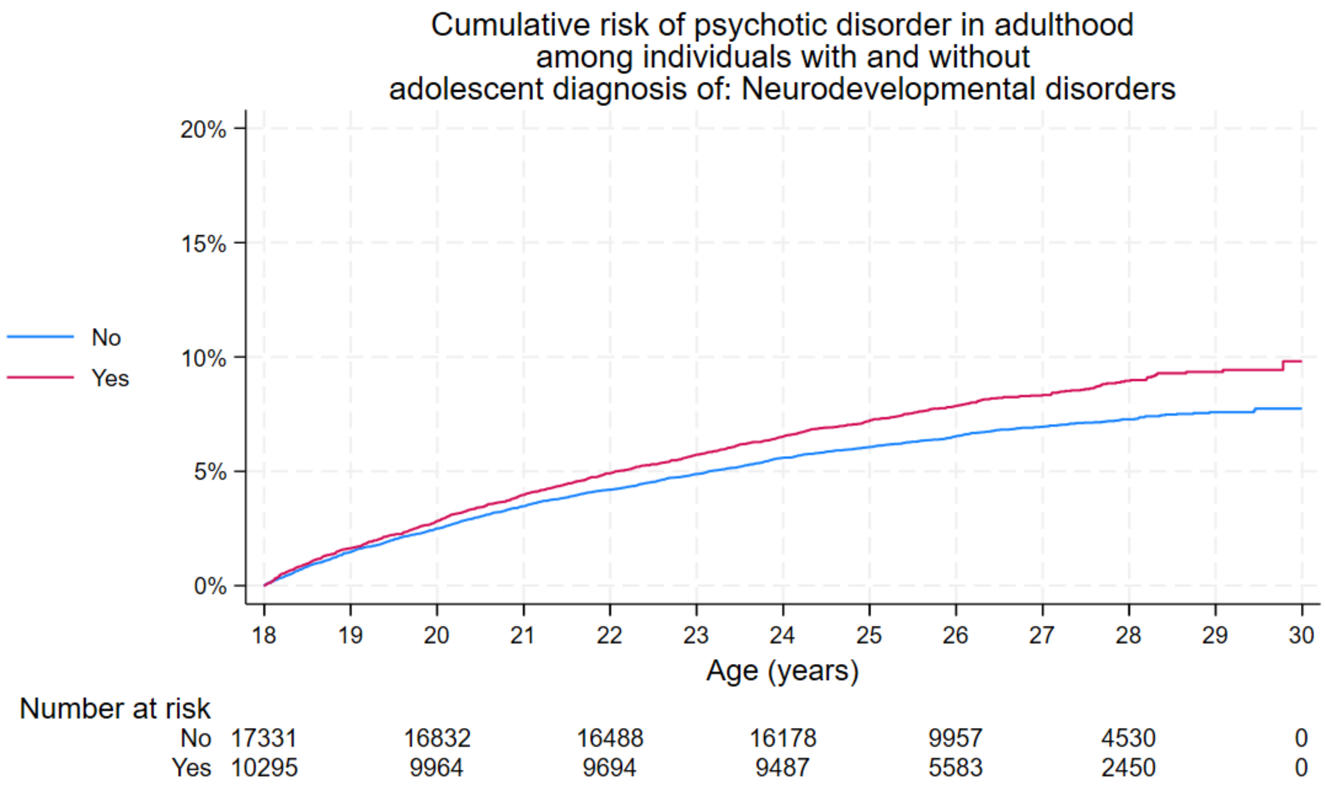

Figure S9. Cumulative risk of psychosis among individuals with and without an adolescent diagnosis of Childhood and adolescence onset disorders.

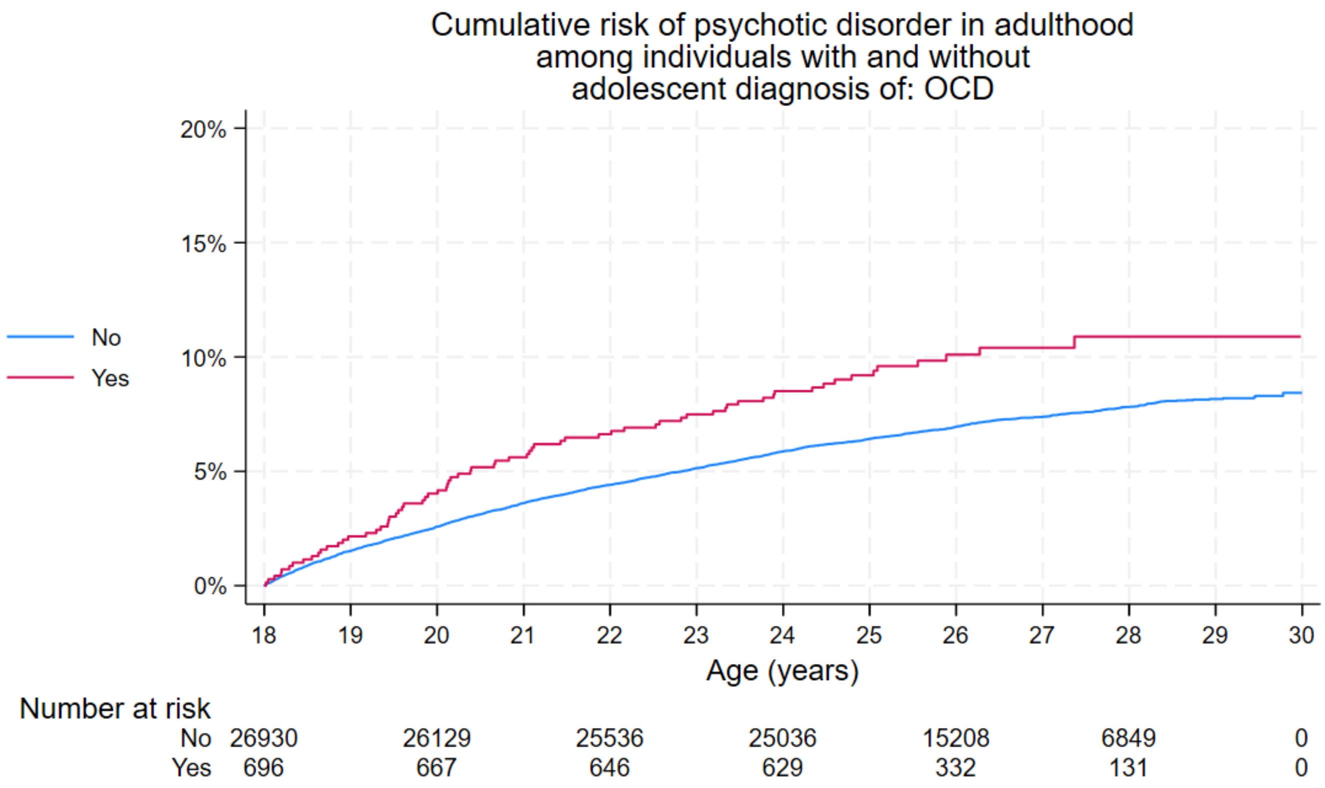

Figure S10. Cumulative risk of psychosis among individuals with and without an adolescent diagnosis of OCD.

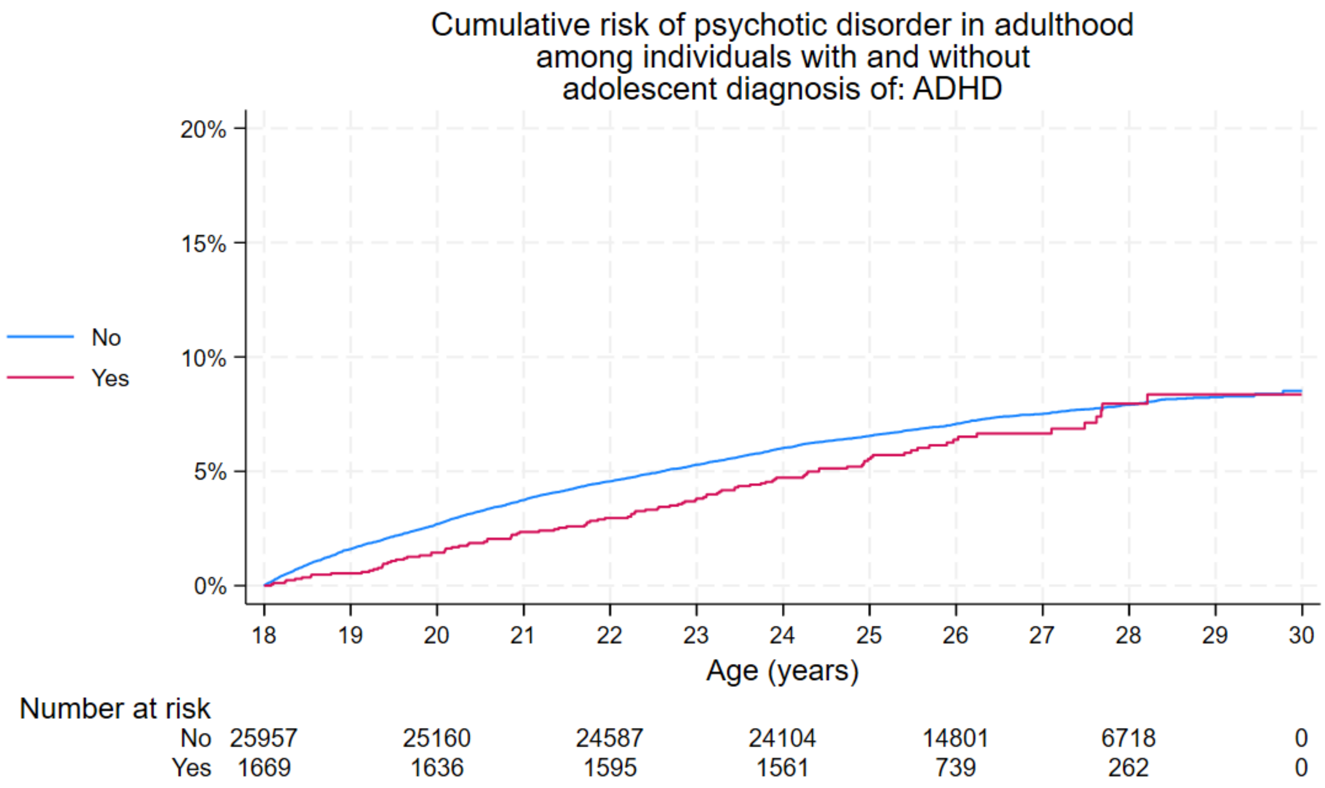

Figure S11. Cumulative risk of psychosis among individuals with and without an adolescent diagnosis of ADHD.

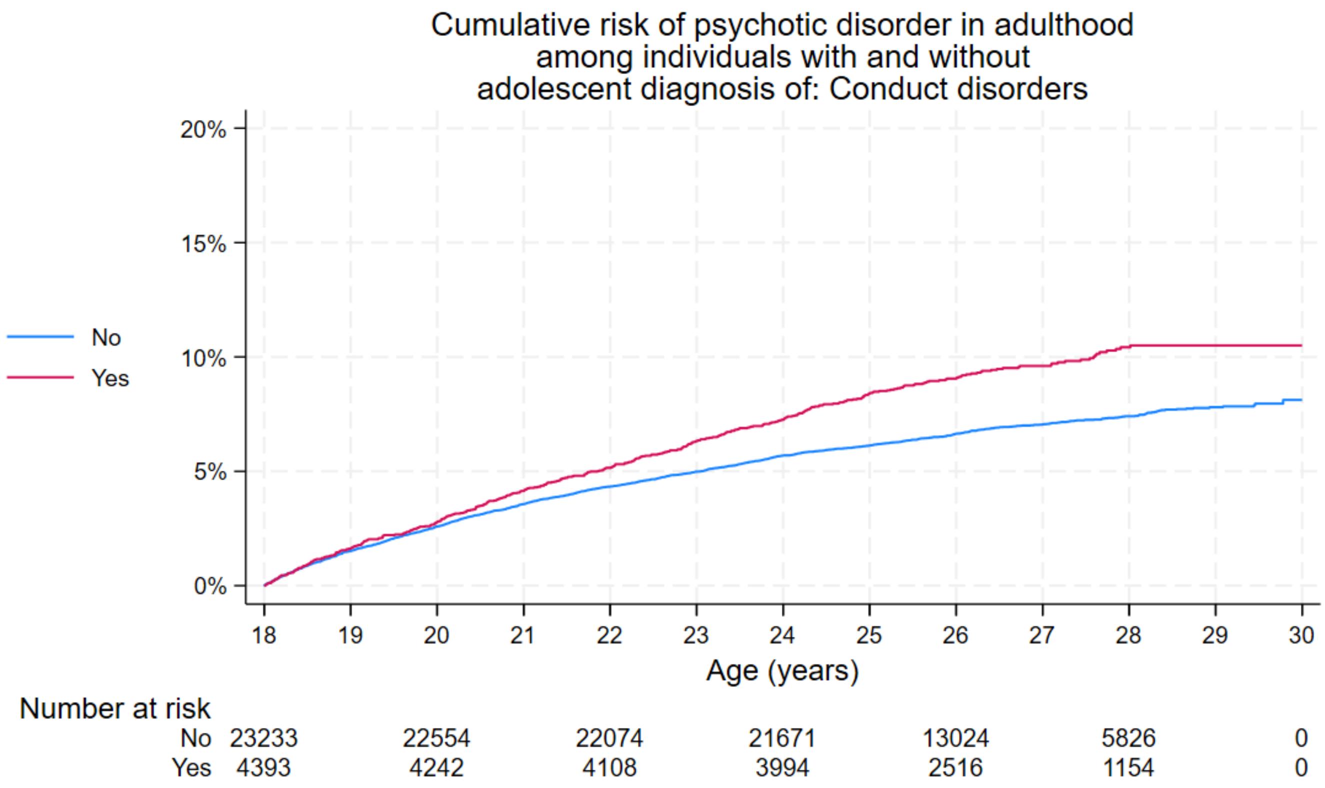

Figure S12. Cumulative risk of psychosis among individuals with and without an adolescent diagnosis of Conduct disorders.

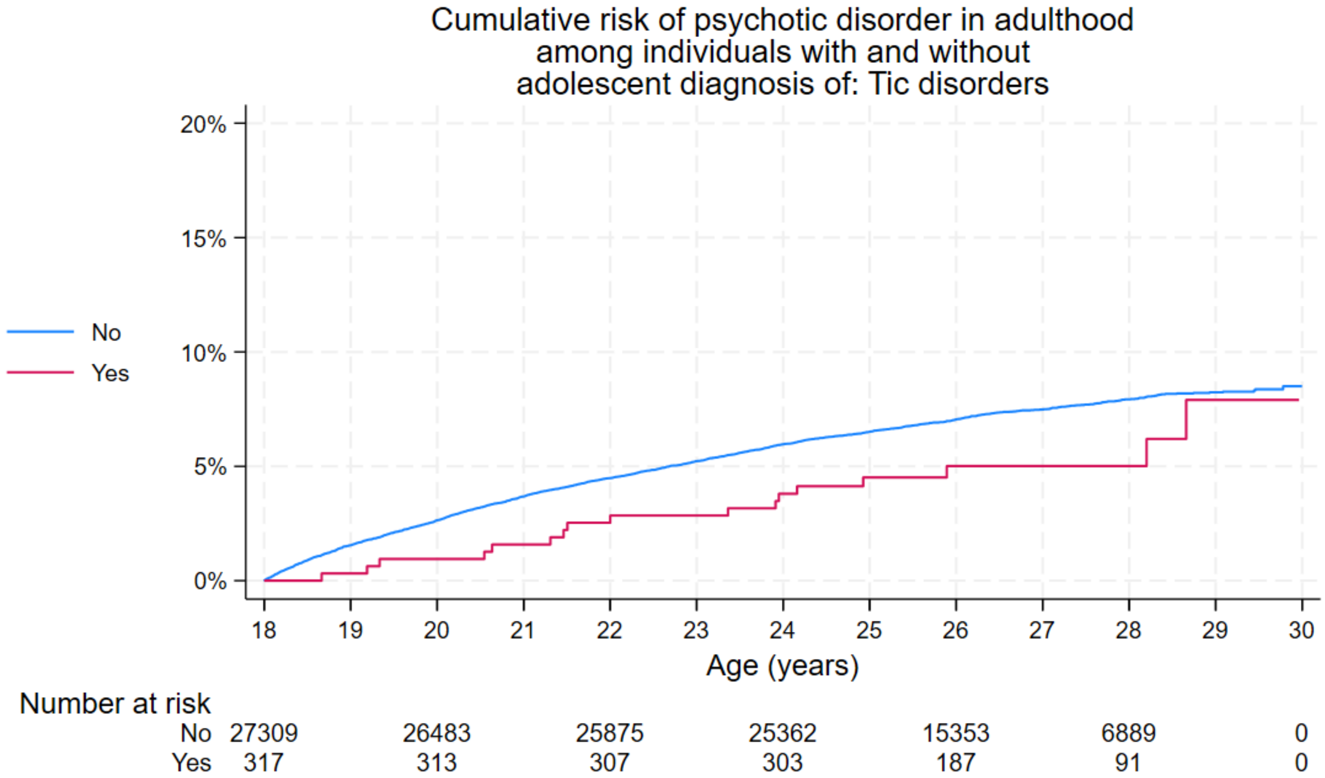

Figure S13. Cumulative risk of psychosis among individuals with and without an adolescent diagnosis of Tic disorders.

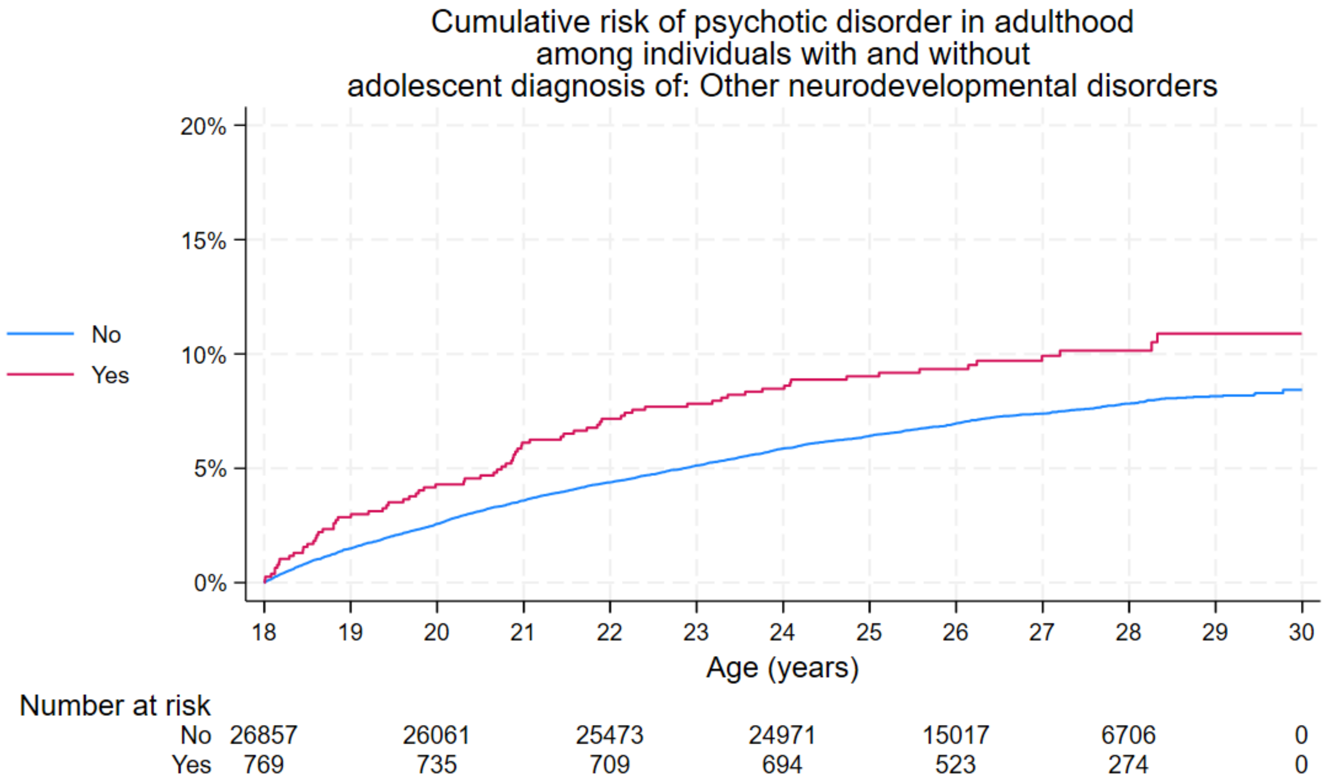

Figure S14. Cumulative risk of psychosis among individuals with and without an adolescent diagnosis of Other neurodevelopmental disorders.

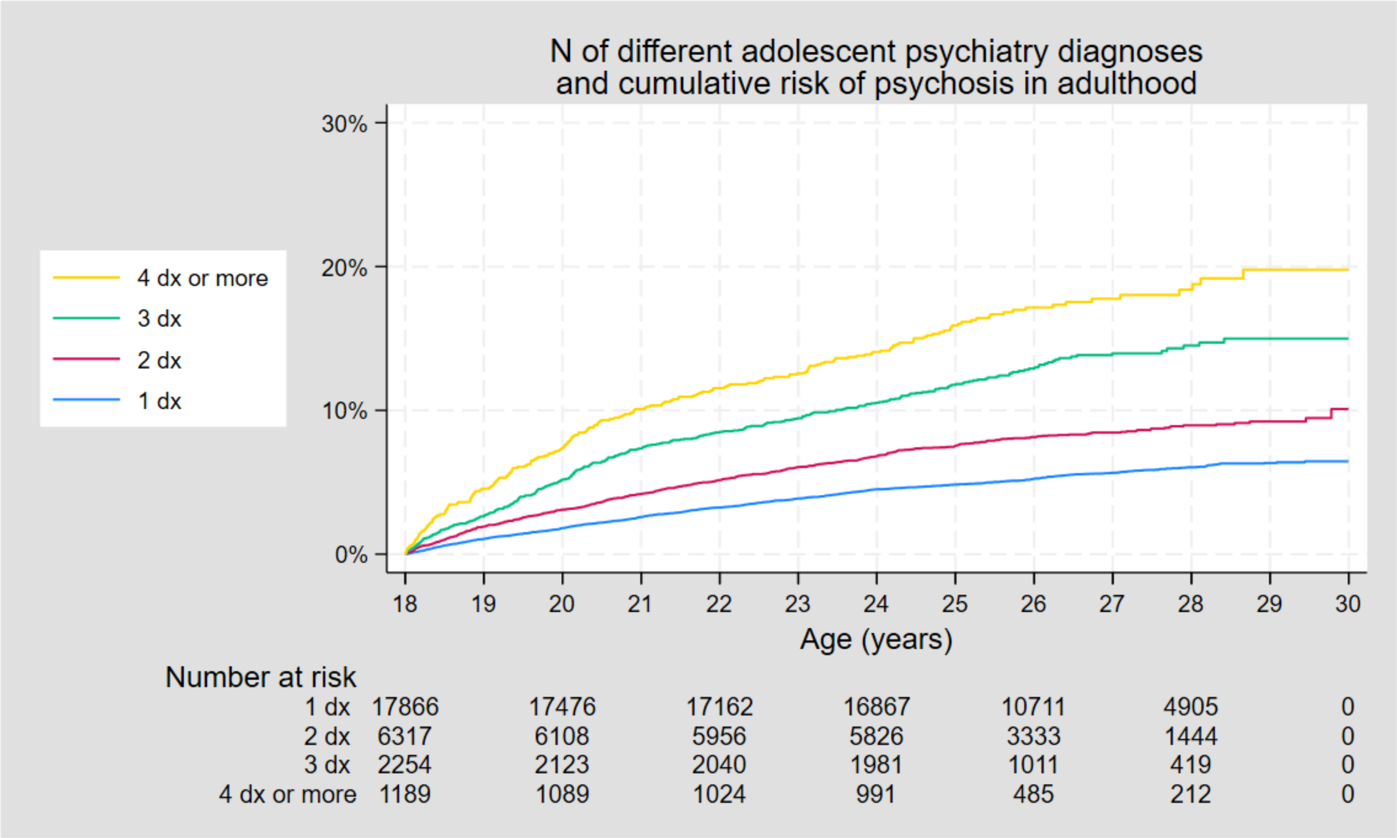

Figure S15. Total number of different mental disorder diagnoses assigned in adolescence and cumulative risk of psychosis in adulthood.

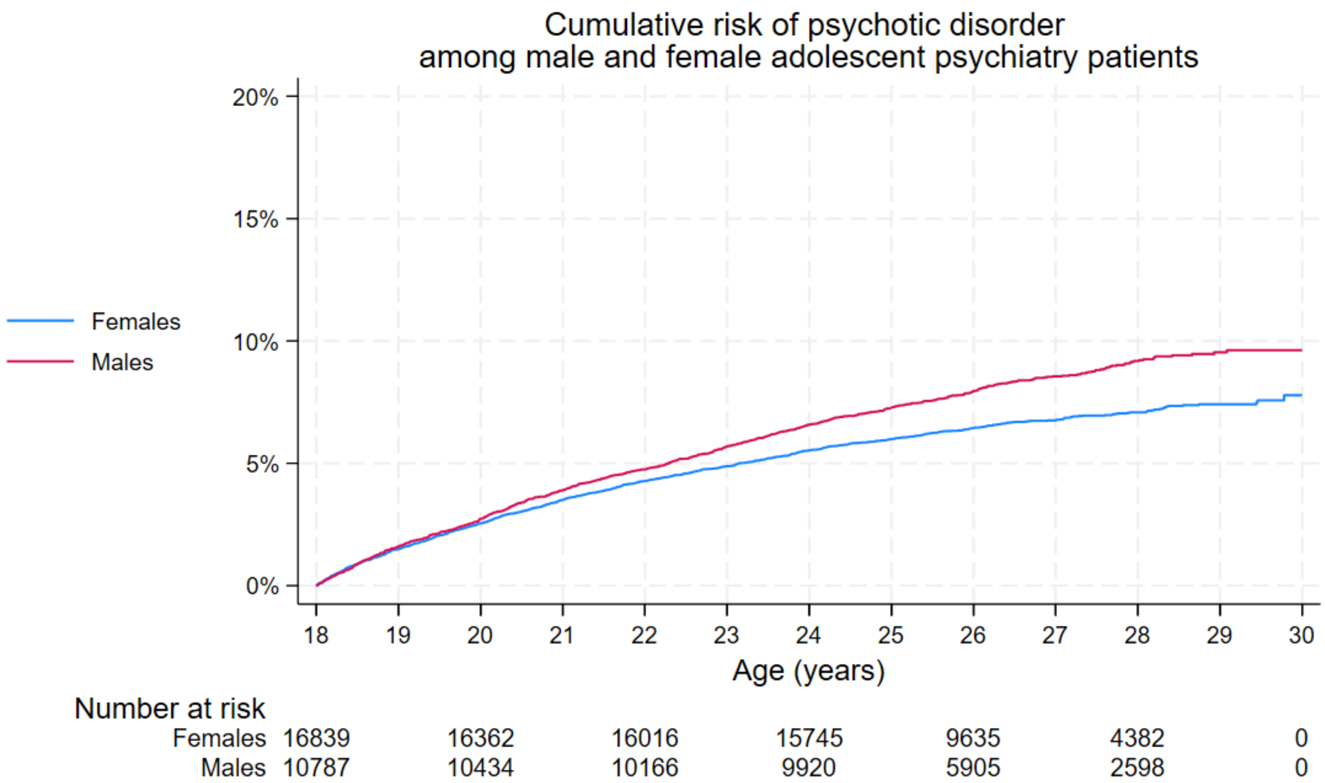

Figure S16. Cumulative risk of psychosis among male and female adolescent psychiatry patients.

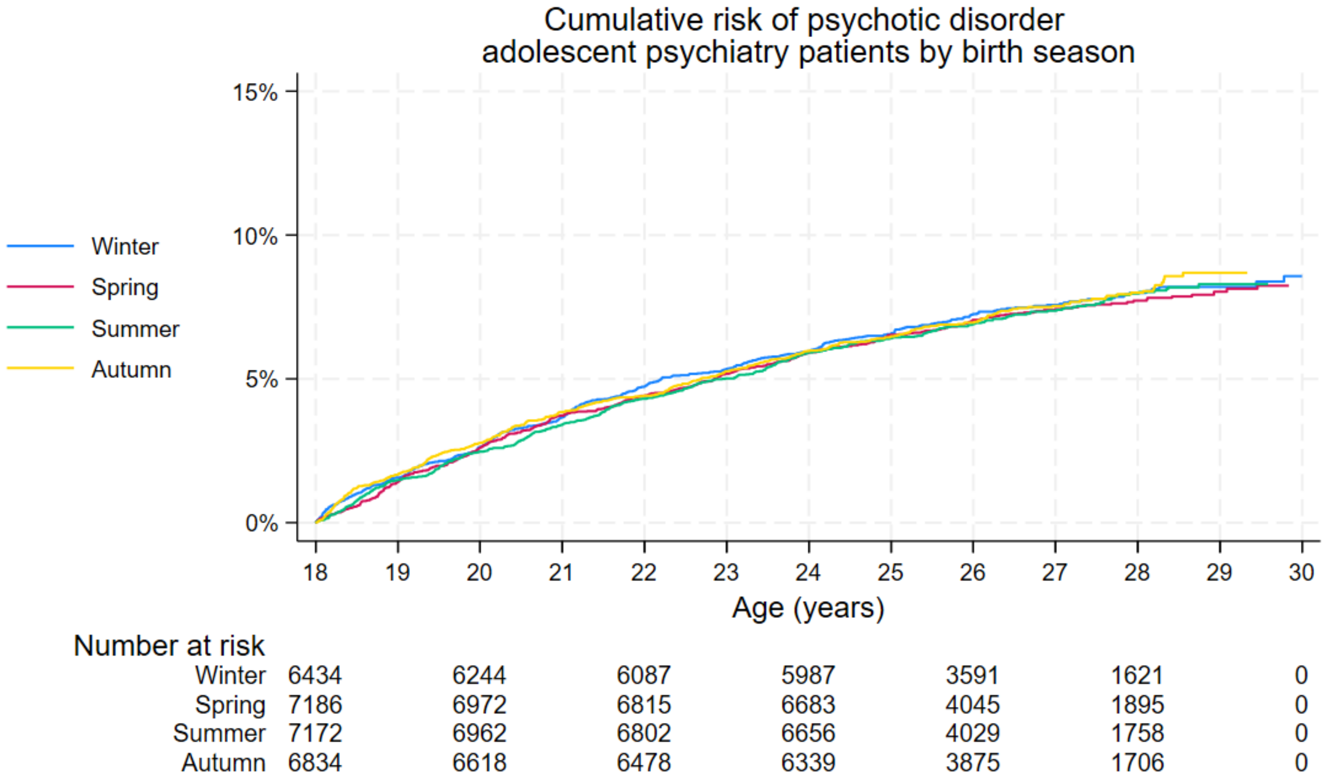

Figure S17. Cumulative risk of psychosis among adolescent psychiatry patients by birth season.

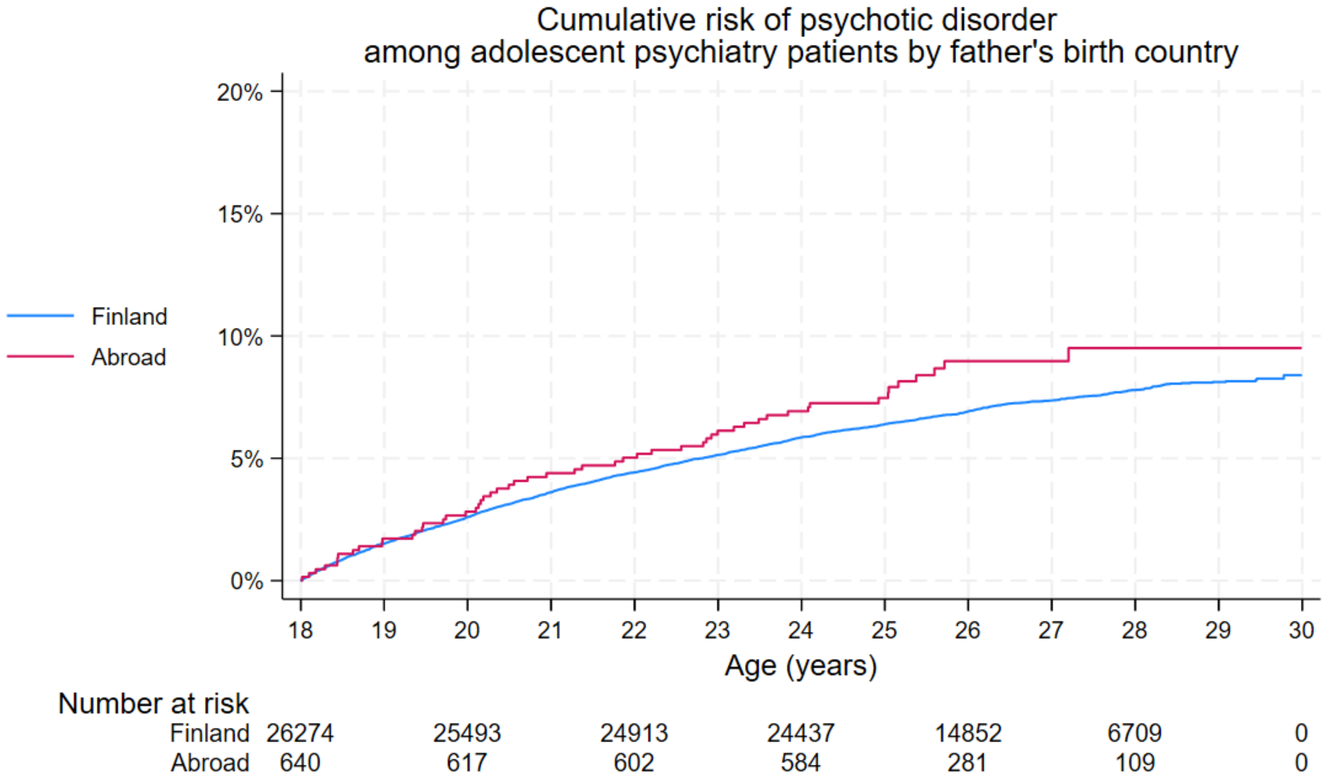

Figure S18. Cumulative risk of psychosis among adolescent psychiatry patients by father's birth country.

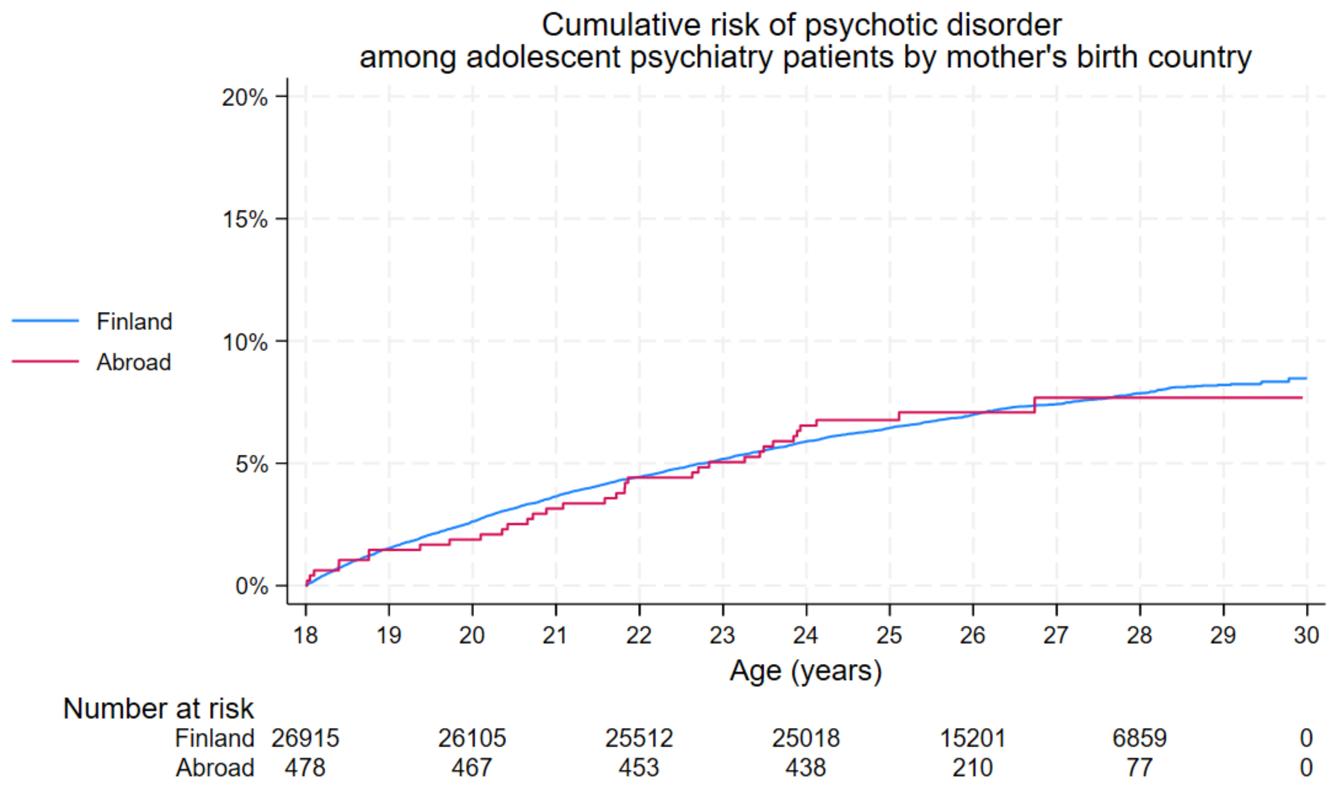

Figure S19. Cumulative risk of psychosis among adolescent psychiatry patients by mother's birth country.

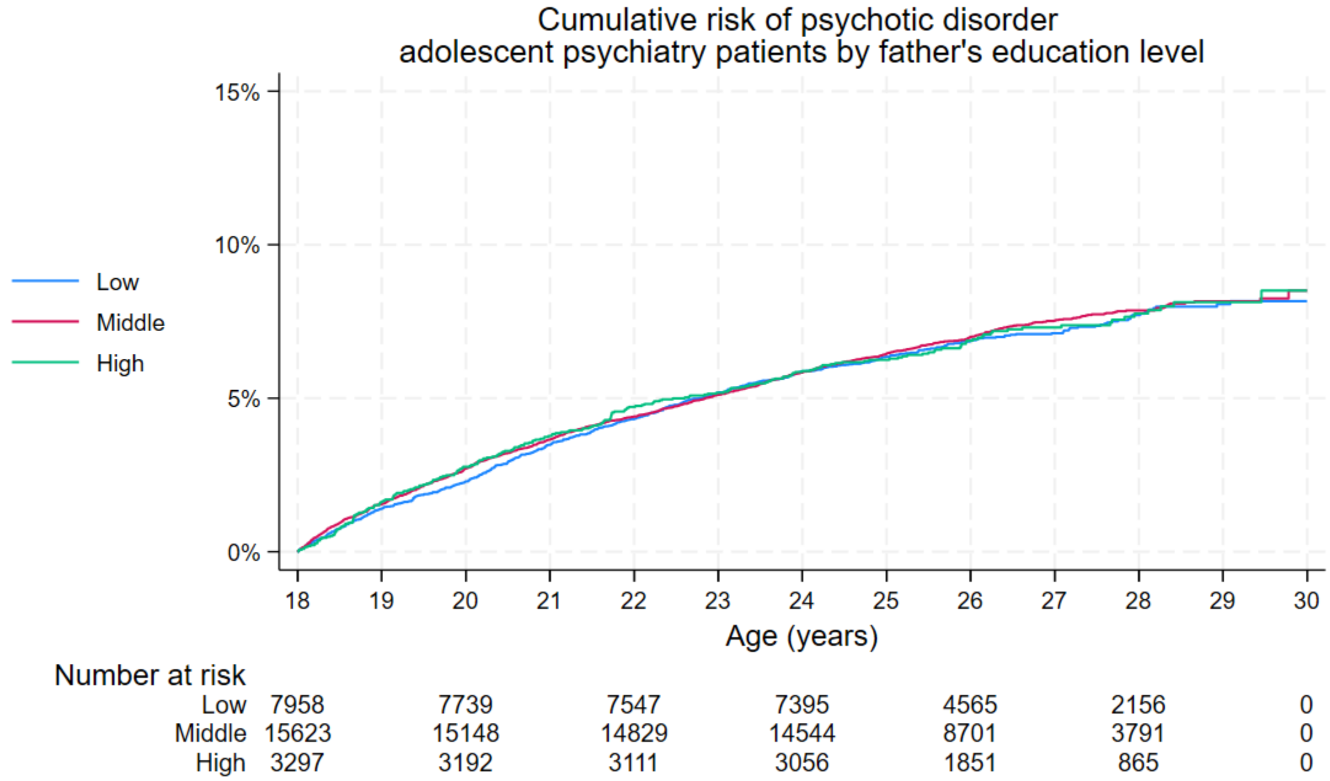

Figure S20. Cumulative risk of psychosis among adolescent psychiatry patients by father’s education level.

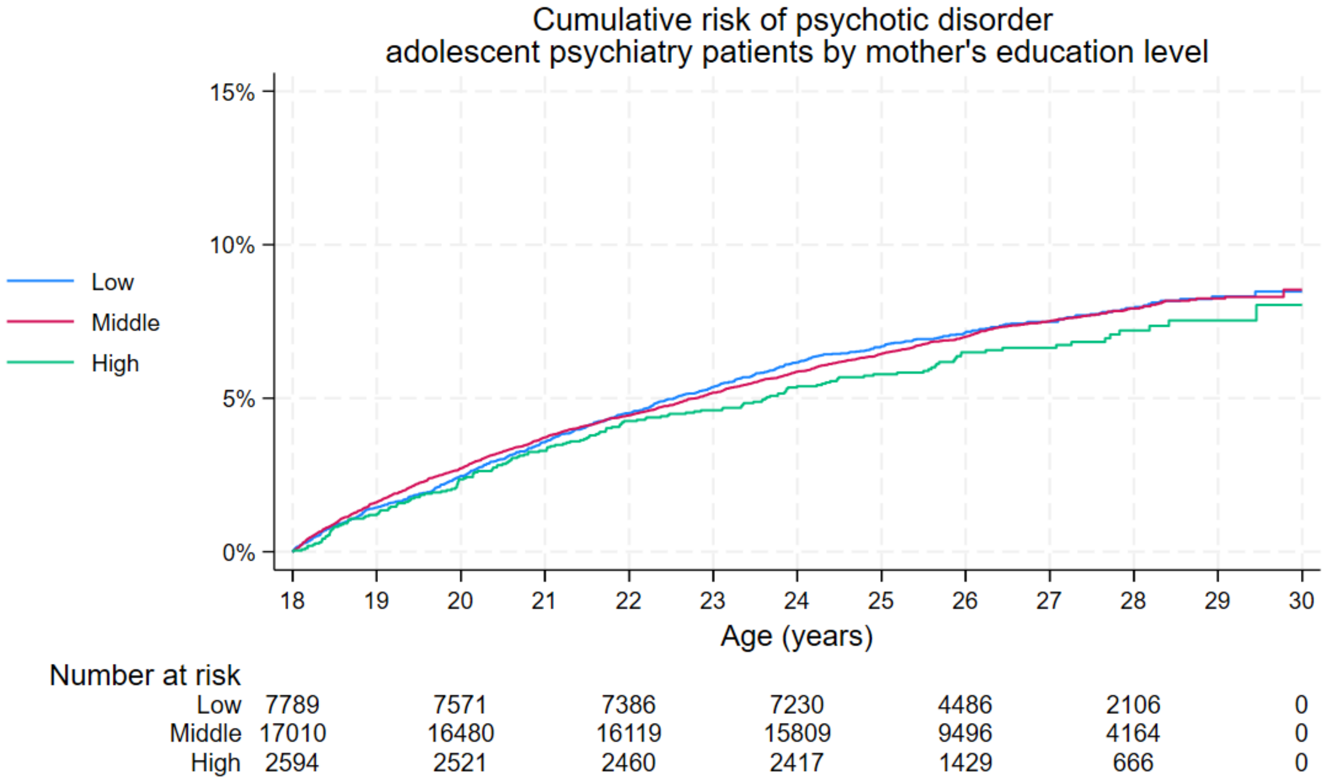

Figure S21. Cumulative risk of psychosis among adolescent psychiatry patients by mother's education level.

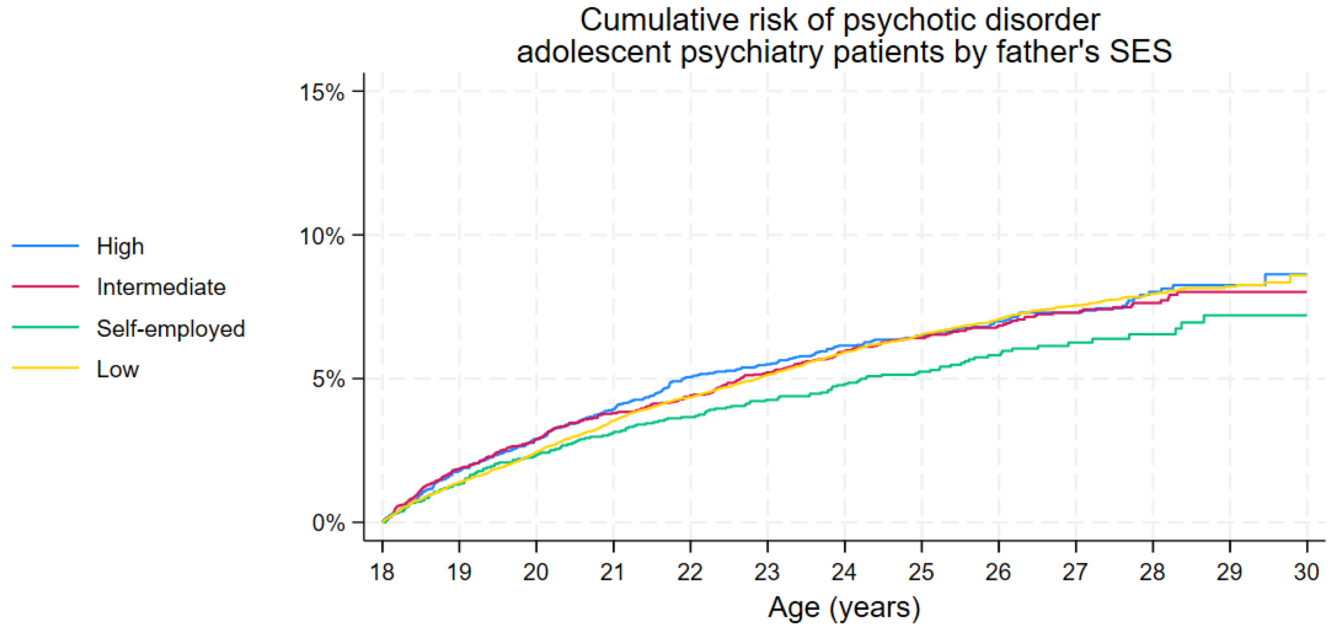

Number at risk

	18	20	22	24	26	28	30
High	3597	3479	3384	3326	1935	858	0
Intermediate	4195	4059	3978	3894	2332	1067	0
Self-employed	2356	2291	2255	2220	1243	526	0
Low	15130	14704	14358	14071	8642	3872	0

Figure S22. Cumulative risk of psychosis among adolescent psychiatry patients by father’s SES.

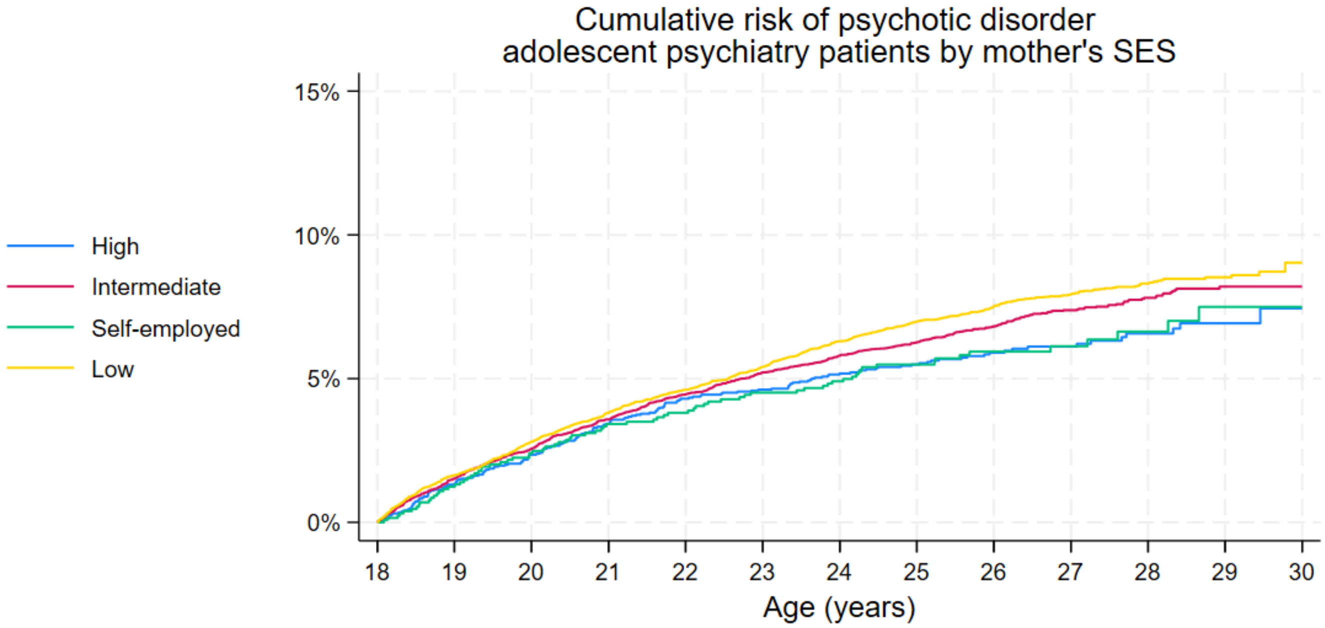

Number at risk

High	2891	2812	2743	2700	1511	644	0
Intermediate	10206	9911	9677	9489	5593	2611	0
Self-employed	1290	1252	1231	1212	699	288	0
Low	11300	10933	10689	10470	6554	2955	0

Figure S23. Cumulative risk of psychosis among adolescent psychiatry patients by mother's SES.

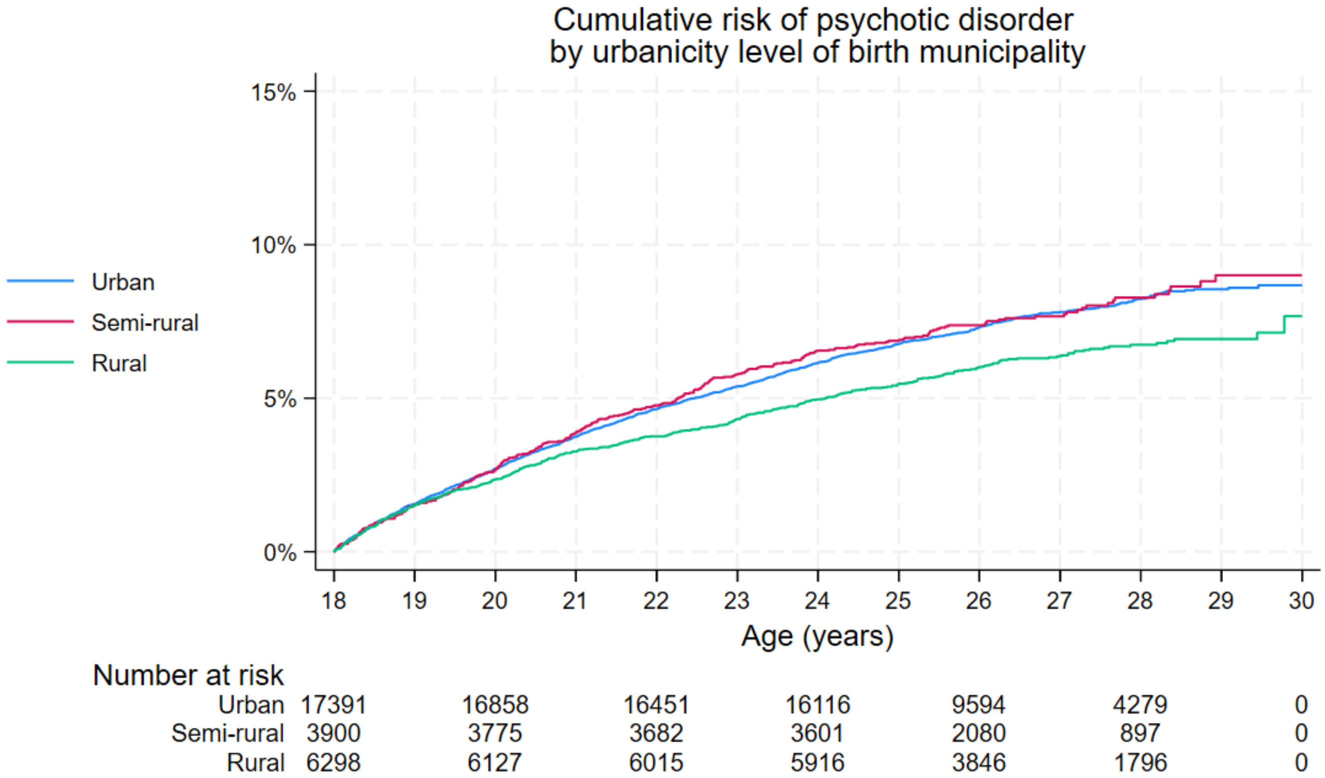

Figure S24. Cumulative risk of psychosis among adolescent psychiatry patients by urbanicity of the birth municipality.

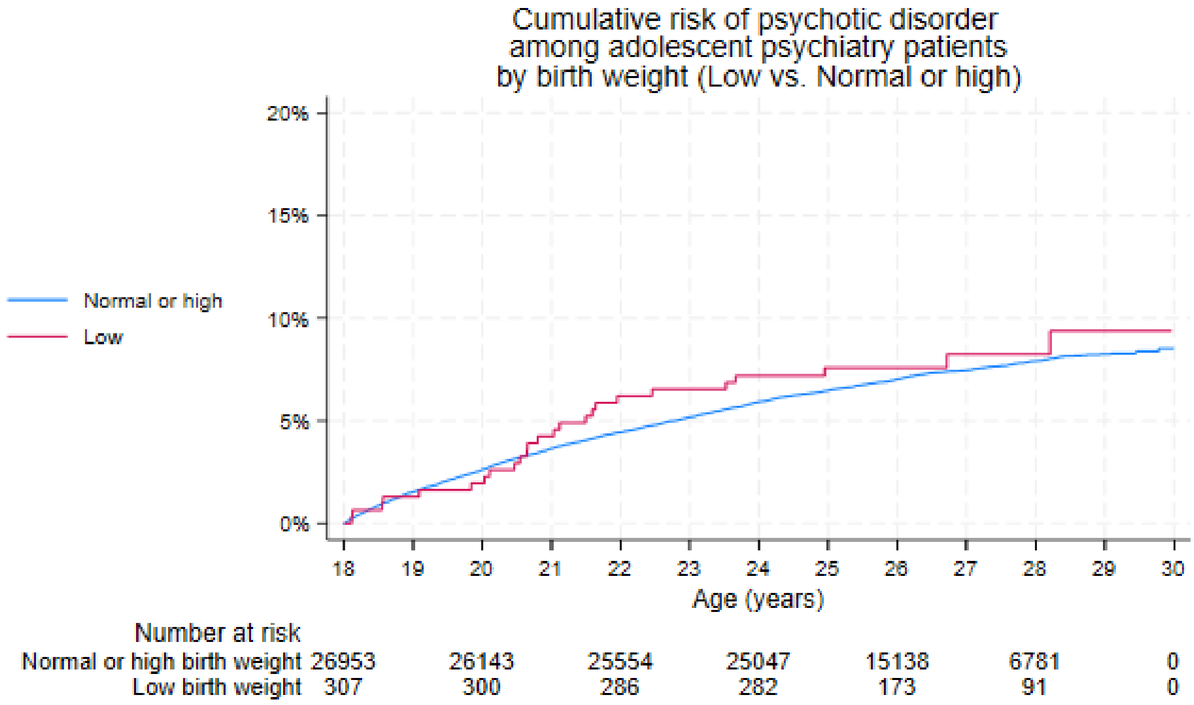

Figure S25. Cumulative risk of psychosis among adolescent psychiatry patients by birth weight (low vs. normal/high for gestational age).

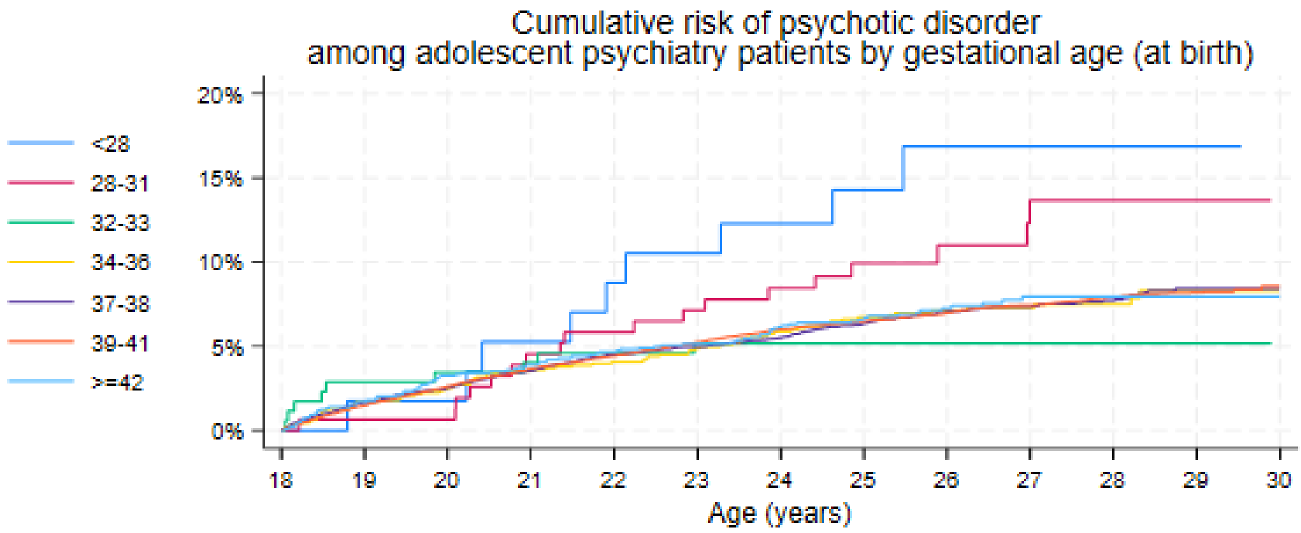

Number at risk

<28	58	56	52	50	27	14	0
28-31	154	153	145	140	78	41	0
32-33	174	168	166	165	88	33	0
34-36	1111	1078	1053	1029	616	279	0
37-38	4860	4711	4596	4533	2743	1247	0
39-41	19678	19093	18663	18272	11115	5005	0
>=42	1286	1243	1222	1194	682	271	0

Figure S26. Cumulative risk of psychosis among adolescent psychiatry patients by gestational age at birth (full weeks)

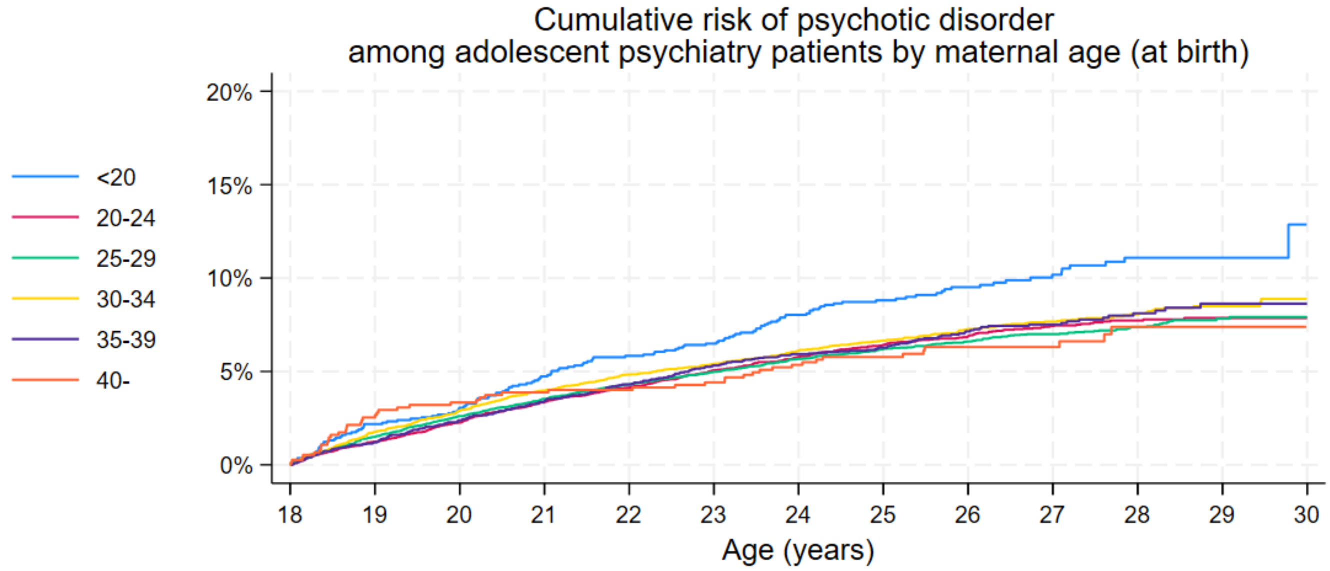

Number at risk

	18	20	22	24	26	28	30
<20	1376	1327	1287	1255	790	396	0
20-24	6316	6148	6011	5882	3660	1617	0
25-29	9472	9192	8987	8815	5265	2352	0
30-34	6729	6505	6353	6241	3733	1697	0
35-39	2984	2901	2830	2770	1653	714	0
40-	749	723	714	702	439	204	0

Figure S27. Cumulative risk of psychosis among adolescent psychiatry patients by maternal age (years, when the child was born).

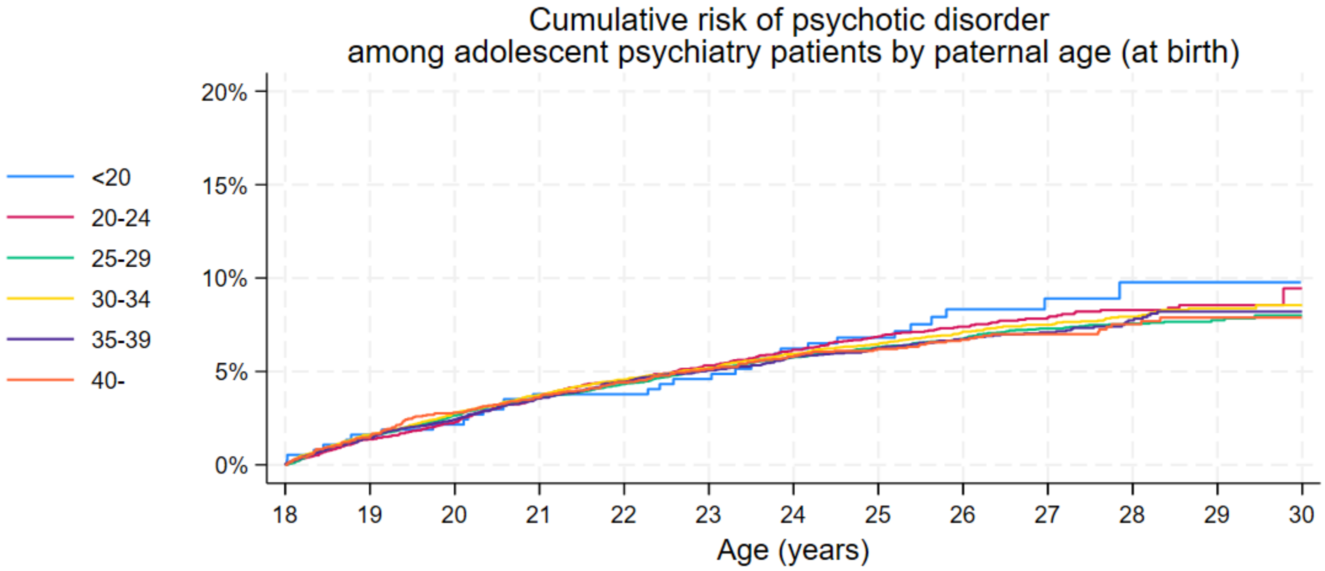

Number at risk

<20	372	361	355	346	214	101	0
20-24	3732	3637	3541	3464	2166	976	0
25-29	8362	8111	7939	7782	4762	2144	0
30-34	7908	7657	7487	7344	4351	1942	0
35-39	4147	4028	3925	3859	2302	1066	0
40-	2394	2317	2269	2227	1338	589	0

Figure S28. Cumulative risk of psychosis among adolescent psychiatry patients by paternal age (years, when the child was born)

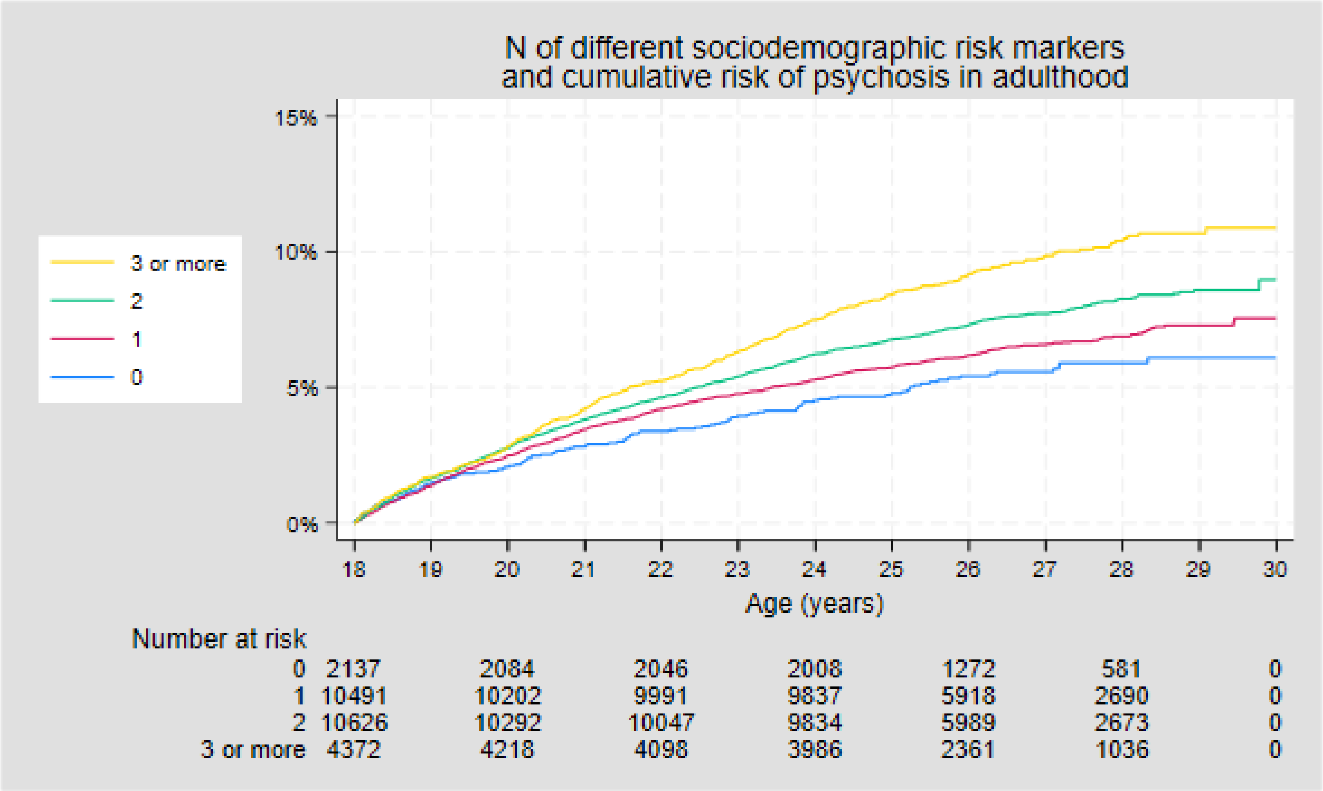

Figure S29. Total number of different sociodemographic prognostic factors and risk of psychosis in adulthood - prognostic factors included: male sex; mother's low SES; born in an urban or semi-rural area; mother aged <20 years; low birth weight; born very or extremely prematurely.

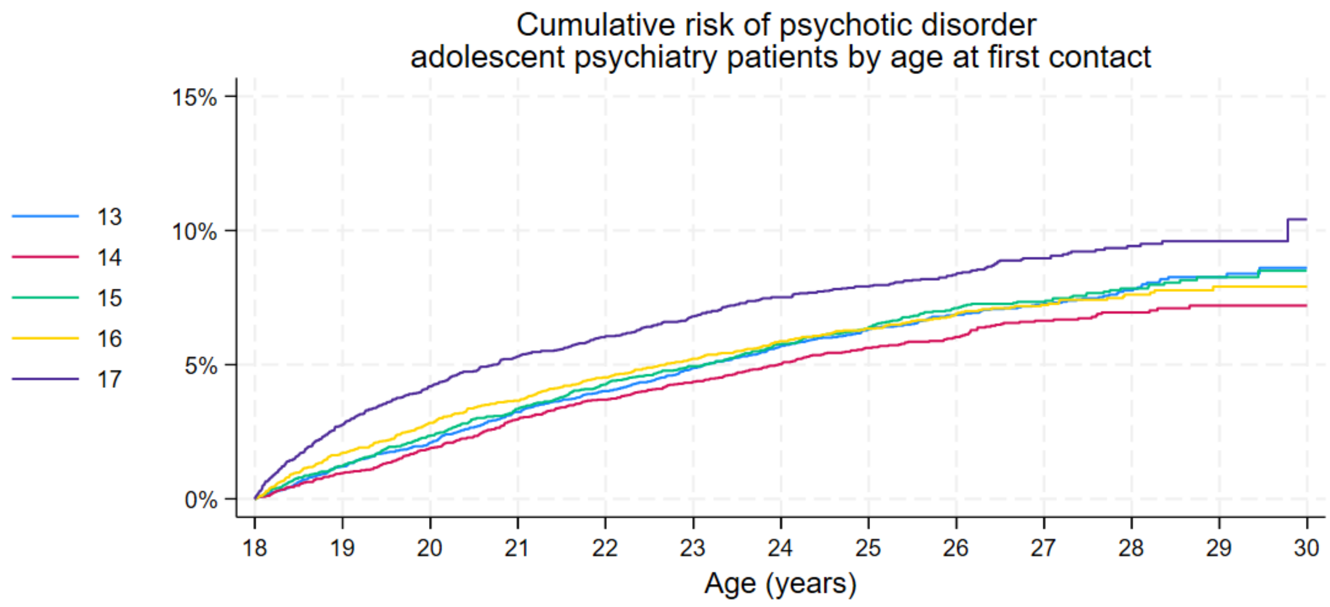

Number at risk

	18	20	22	24	26	28	30
13	6749	6589	6424	6288	3710	1663	0
14	5264	5151	5039	4945	3066	1401	0
15	5425	5275	5159	5055	3021	1442	0
16	5237	5063	4952	4864	2935	1301	0
17	4951	4718	4608	4513	2808	1173	0

Figure S30. Cumulative risk of psychosis among adolescent psychiatry patients by age at first adolescent psychiatry contact.

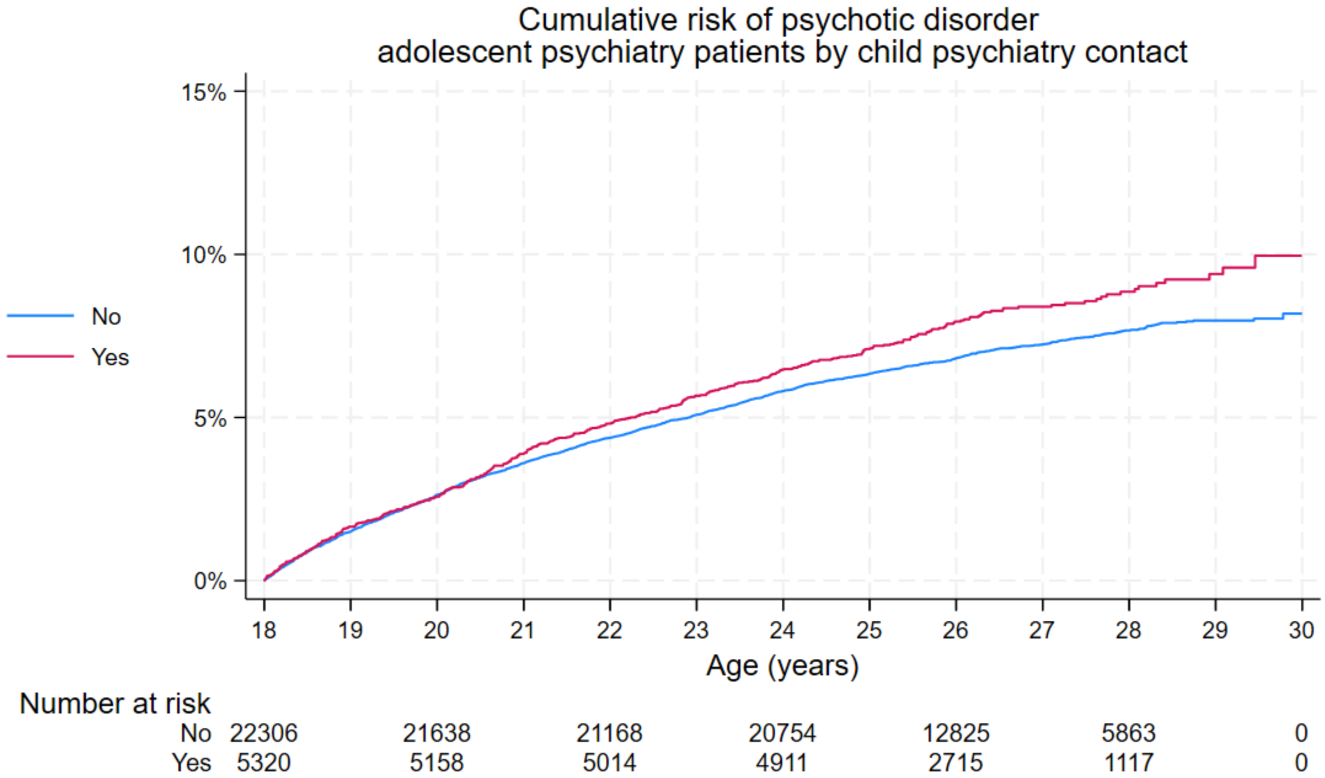

Figure S31. Cumulative risk of psychosis among adolescent psychiatry patients by child psychiatry contact.

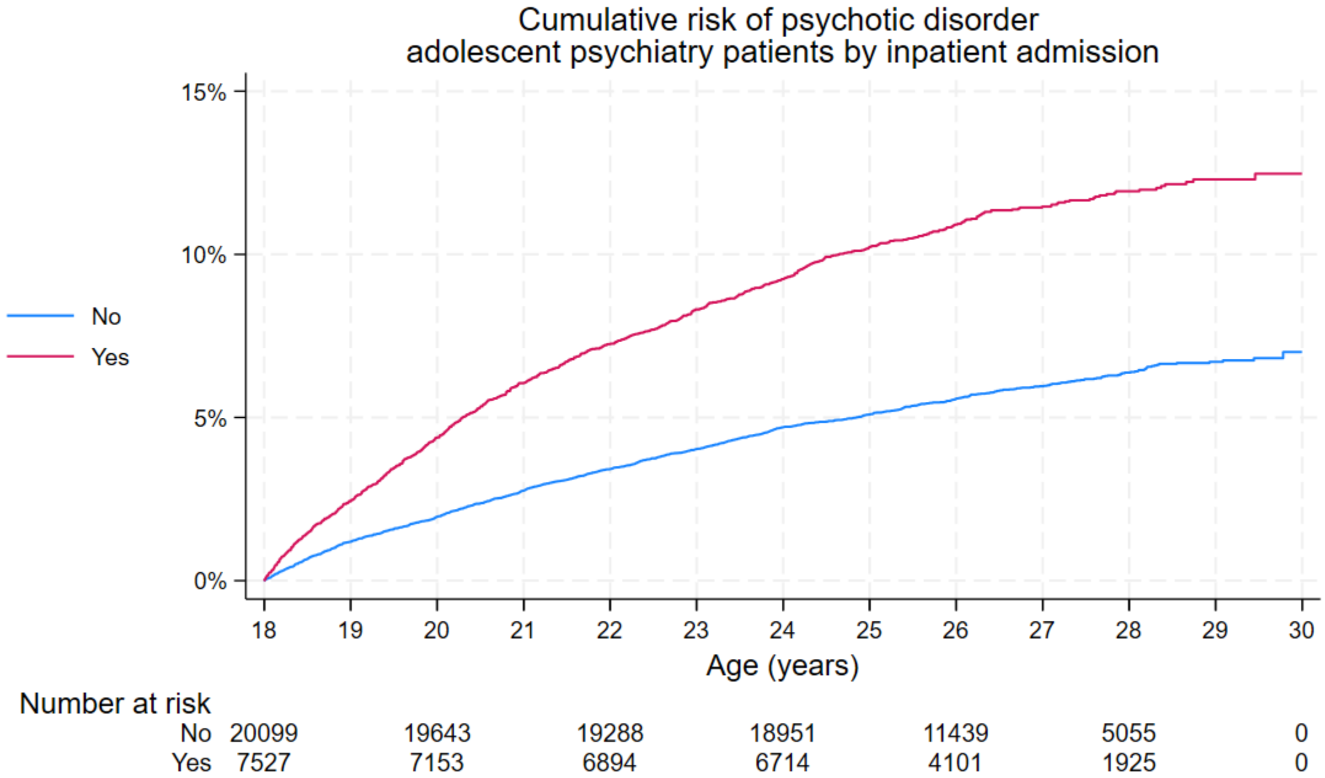

Figure S32. Cumulative risk of psychosis among adolescent psychiatry patients by having a psychiatric inpatient admission in adolescence.

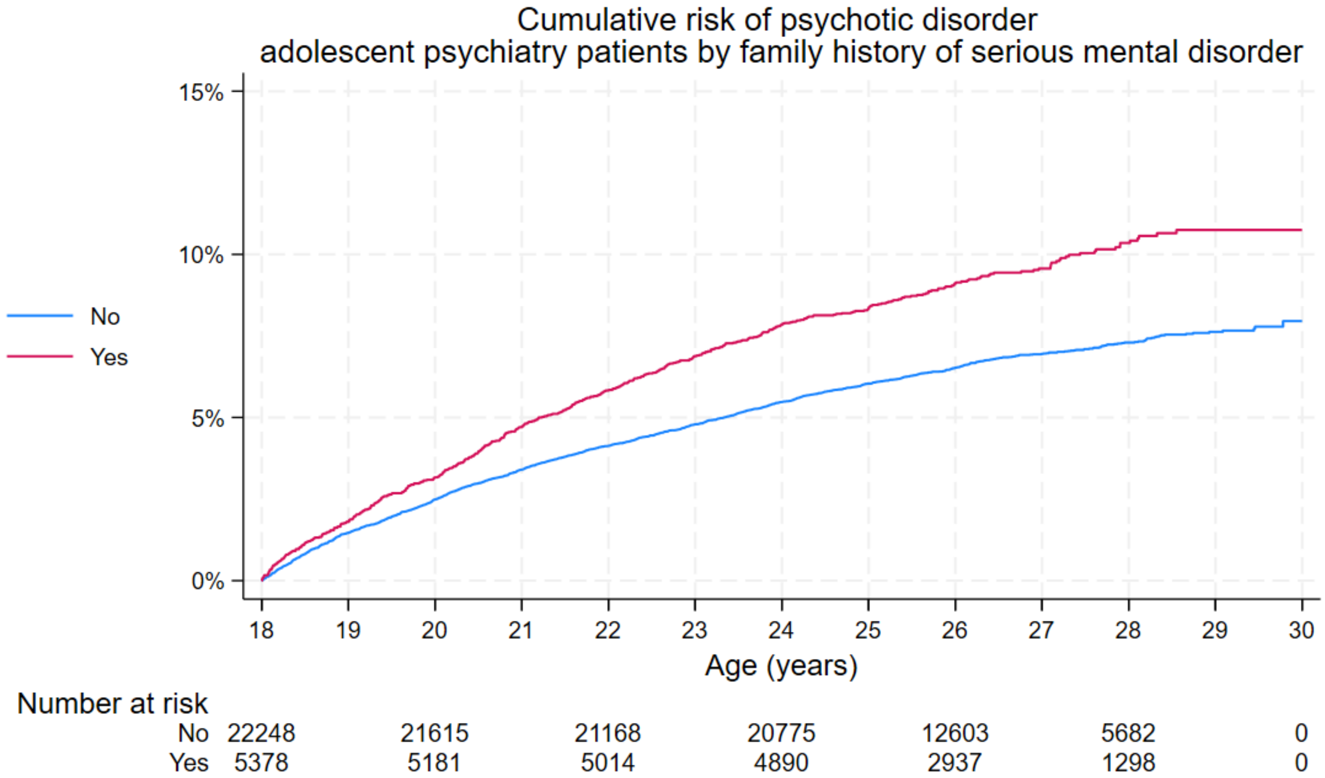

Figure S33. Cumulative risk of psychosis among adolescent psychiatry patients by parental history of serious mental disorder.

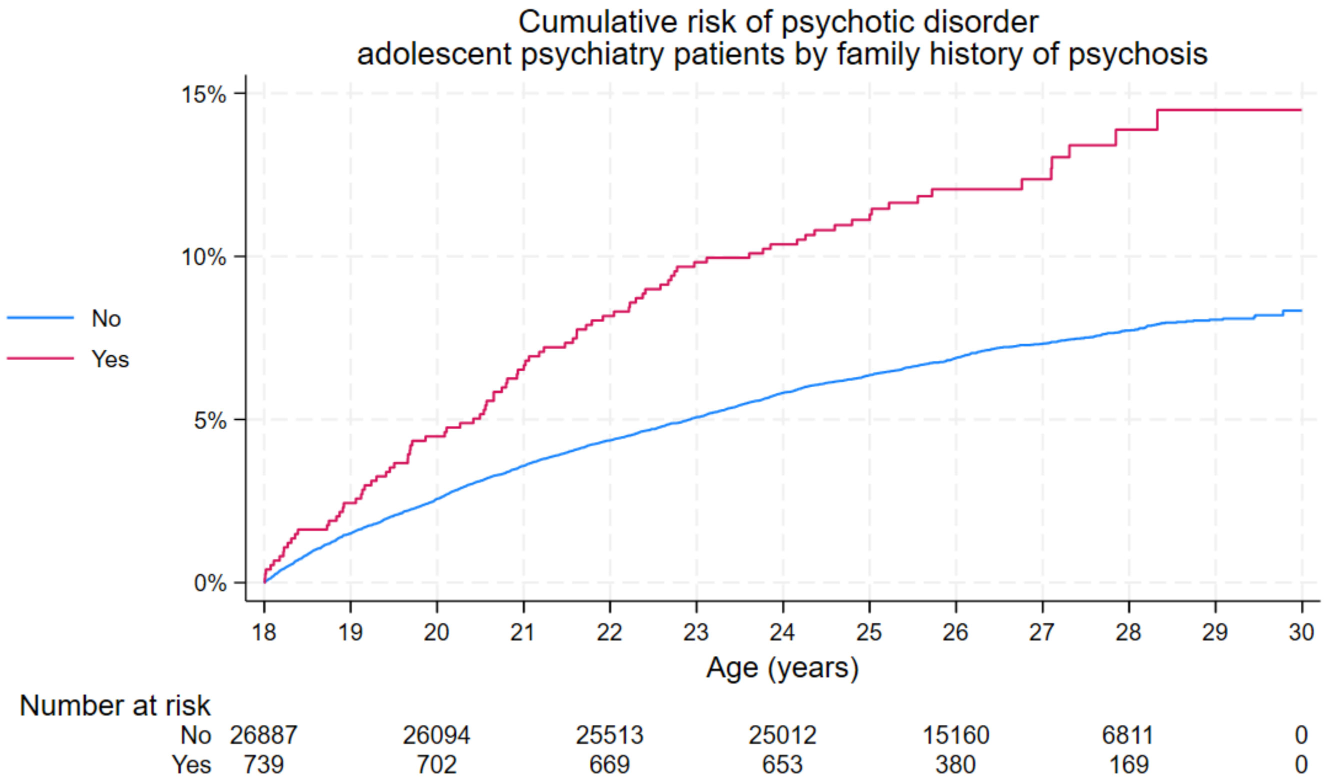

Figure S34. Cumulative risk of psychosis among adolescent psychiatry patients by parental history of psychotic disorder.

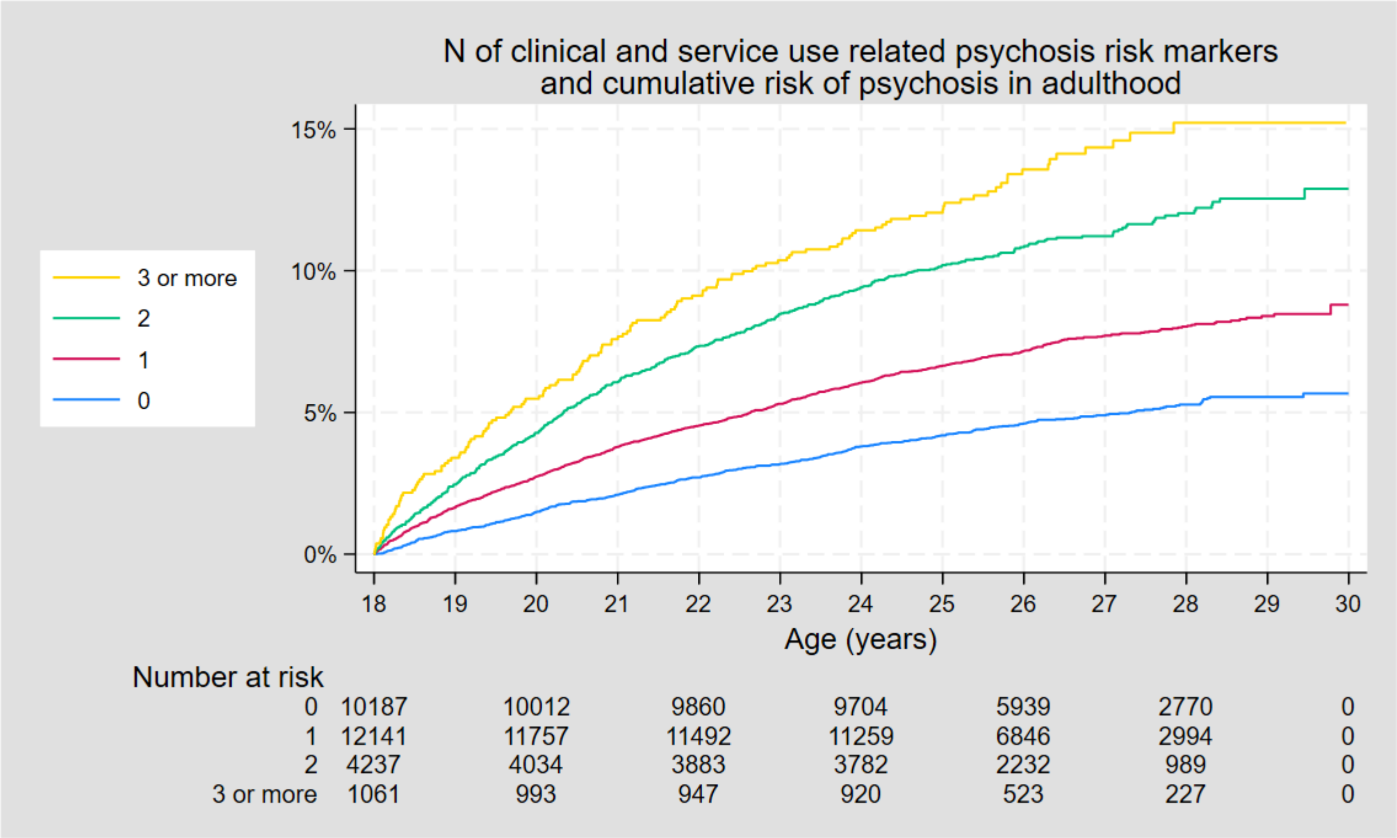

Figure S35. Total number of different clinical and service use related prognostic factors and risk of psychosis in adulthood - prognostic factors included: attended adolescent psychiatry services first time when aged 17 years; child psychiatry visit; psychiatric inpatient admission in adolescence; family history of serious mental illness; family history of psychosis.
